# Salamandamide Lipodipeptides Are Biosynthetic Intermediate Shunt Products of the Nonamodular Nonribosomal Peptide Assembly Lines of the Viscosin Family

**DOI:** 10.1021/acs.jnatprod.5c00084

**Published:** 2025-04-15

**Authors:** Keshab Bhattarai, Thomas Majer, Manuela Haussmann, Dieter Schollmeyer, Markus Kramer, Feyisara Eyiwumi Oni, Monica Höfte, Rabea Voget, Michael Gütschow, Natalia Ruetalo, Michael Schindler, Jan Straetener, Tatjana Wannenwetsch, Heike Brötz-Oesterhelt, Ryan Karongo, Benedikt Masberg, Michael Lämmerhofer, Rosanna Catherine Hennessy, Carly R. Muletz-Wolz, Harald Gross

**Affiliations:** † Pharmaceutical Institute, Department of Pharmaceutical Biology, University of Tübingen, 72076 Tübingen, Germany; ‡ Department of Chemistry, Johannes Gutenberg University Mainz, 55099 Mainz, Germany; § Institute of Organic Chemistry, University of Tübingen, 72076 Tübingen, Germany; ⊥ Department of Phytopathology, Rijk Zwaan Breeding B.V., 2678 ZG De Lier, The Netherlands; ∥ Lab. Phytopathology, Department of Plants and Crops, Faculty of Bioscience Engineering, Ghent University, 9000 Gent, Belgium; ▽ Pharmaceutical Institute, Pharmaceutical & Medicinal Chemistry, University of Bonn, 53121 Bonn, Germany; ○ Institute for Medical Virology and Epidemiology, Section Molecular Virology, University Hospital Tübingen, 72076 Tübingen, Germany; # Department of Microbial Bioactive Compounds, Interfaculty Institute of Microbiology and Infection Medicine, Tübingen (IMIT), University of Tübingen, 72076 Tübingen, Germany; ◆ German Center for Infection Research (DZIF), partner site Tübingen, 72076 Tübingen, Germany; ¶ Cluster of Excellence: EXC 2124: Controlling Microbes to Fight Infection, University of Tübingen, 72076 Tübingen, Germany; + Pharmaceutical Institute, Department of Pharmaceutical Analysis and Bioanalysis, University of Tübingen, 72076 Tübingen, Germany; ■ Department of Plant and Environmental Sciences, University of Copenhagen, 1870 Frederiksberg, Denmark; ● Center for Conservation Genomics, Smithsonian’s National Zoo and Conservation Biology Institute, Washington, D.C. 20008, United States

## Abstract

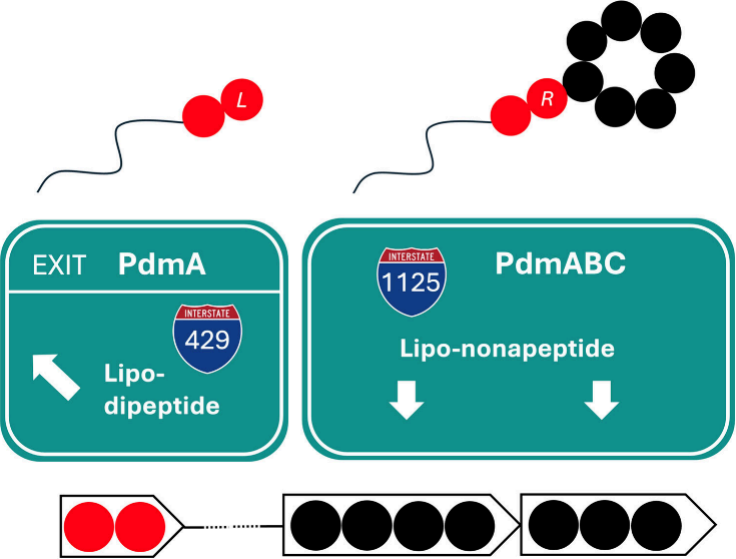

Chemical investigation of a salamander-mucus-associated *Pseudomonas tolaasii* strain led to the isolation and chemical characterization of salamandamide A, a new lipo-dipeptide, along with known lipopeptides of the pseudodesmin and tolaasin class. Genome mining revealed that no specific gene cluster codes for the biosynthesis of salamandamide A. Stereochemical analyses and mutagenesis experiments linked the biosynthesis of the lipo-dipeptide salamandamide A to the NRPS gene cluster of the lipo-nonapeptide pseudodesmin. Further chemical investigations showed that this finding appears to be a broader concept and that all nonamodular NRPS gene clusters of the viscosin family were capable to produce, beside the expected lipo-nonapeptide, the corresponding lipo-dipeptide as a shunt product which also led to the discovery of salamandamide B from *Pseudomonas lactis* SS101.

Soil-dwelling, plant- and animal-associated pseudomonads are prominent producers of lipopeptides.^1−4^ They show remarkable structural diversity and vary in length and composition of the lipid moiety as well as in the type, number, and absolute configuration of the amino acids in the peptide portion. The currently known *Pseudomonas* lipopeptides are classified in 14 chemical families.^5^ This chemical diversity represents a large chemical space which in turn can be translated into a broad range of bioactivities which are relevant in an ecological, agricultural or pharmaceutical context.^5−7^ The biosynthesis of *Pseudomonas* lipopeptides is governed by nonribosomal peptide synthetases (NRPSs). NRPSs are mega-enzymes organized in modules comprising three core domains: (i) the adenylation (A) domain which selects and activates a specific amino acid by adenylation; (ii) the thiolation (T) domain (*syn*. peptidyl carrier protein–PCP) which carries the growing peptidyl chain and (iii) the condensation (C) domain, which catalyzes the peptide bond formation.^8^ The resulting linear peptide chain is then released from the NRPS complex by the action of a thioesterase (TE) domain which is commonly present in the termination module.^9^ Notably, the *Pseudomonas* NRPS-systems coding for lipopeptides usually feature a tandem-TE-domain, i.e. two TE domains in a row with different functions.^10^ The first represents a type I TE, which cleaves the lipopeptide from the NRPS system and optionally cyclizes its substrate to a cyclic lipopeptide, while the second is a type II TE with editing function which removes acyl groups that have been misconjugated to T domains.^9,11^ A further peculiarity is the absence of standalone epimerization (E) domains, which invert the stereochemistry at the α-atom of an amino acid, thereby converting L-amino acids into D-amino acids during the biosynthesis process. In *Pseudomonas* lipopeptide gene clusters, this catalytic activity is hidden in combined condensation/epimerization (C/E) domains.^10,12,13^ The lipid chain is introduced by a special C-starter (C_S_) domain, which is located N-terminally in the first module and *N*-acylates the first amino acid.^10,13,14^ Commonly, biosynthetic gene clusters (BGCs) are clustered in prokaryotes, which also holds true for the majority of the lipopeptide NRPS BGCs. However, exemptions exist where the structural NRPS genes are physically directly linked but located at different loci in the corresponding *Pseudomonas* genome. This applies for the xantholysin- and entolysin-BGC^15,16^ as well for several members of the viscosin group.^17−20^ Intriguingly, these so-called split-NRPS or bipartite BGCs are functional and have always resulted so far solely in the production of the complete molecule. Here, we demonstrate that the *N*-terminal structural bimodular NRPS genes can work independently and produce linear lipo-dipeptides.

## Results and Discussion

### Identification of the Lipo-octodecapeptide Tolaasin C and Isolation of the Lipo-dipeptide Salamandamide (2) and Lipo-nonapeptides 3 and 4 from *Pseudomonas* sp. RSB 5.11

As part of our ongoing program to explore the chemical constituents of pseudomonads, we investigated the strain *Pseudomonas* sp. RSB 5.11, which was originally isolated from the skin microbiome of a *Plethodon* salamander and shown to exhibit antifungal activity.^21^ To gain insights into the metabolome of RSB 5.11 and for dereplication purposes, the strain was cultivated small-scale in liquid media, the crude extract evaluated by LC-HR-MS/MS, and the data processed through Global Natural Product Social Molecular Networking (GNPS).^22^ The molecular networking analysis of the crude extract of strain RSB 5.11 yielded 669 nodes in 75 compound clusters (Figure S1). Two molecular ion clusters were identified as lipopeptide clusters and were, therefore, of further interest for us. One mass cluster contained nodes at *m*/*z* 2004.2 and 1986.2 which were, based on its MS/MS fragmentation pattern, tentatively dereplicated as tolaasin C (**1**) and derivatives (Figure S2).^23^ A further mass cluster presented nodes at *m*/*z* 1110.7, 1124.7, and 429.3. The latter mass could not be dereplicated, while the other two masses could be identified as one of the isobaric epimeric decalipo-nonapeptide pairs pseudodesmin A/viscosinamide A and pseudodesmin B/viscosinamide B, respectively.^24^ In order to obtain sufficient amounts of the observed lipopeptides, upscaled cultures (14 L) were fermented, and the resultant crude extract was fractionated LC-MS-guided, leading to the purification of compound **2** (*m*/*z* 430.3 [M + H]^+^) and the lipononapeptides **3** (*m*/*z* 1125.7 [M + H]^+^) and **4** (*m*/*z* 1111.7 [M + H]^+^).

**Figure 1:**
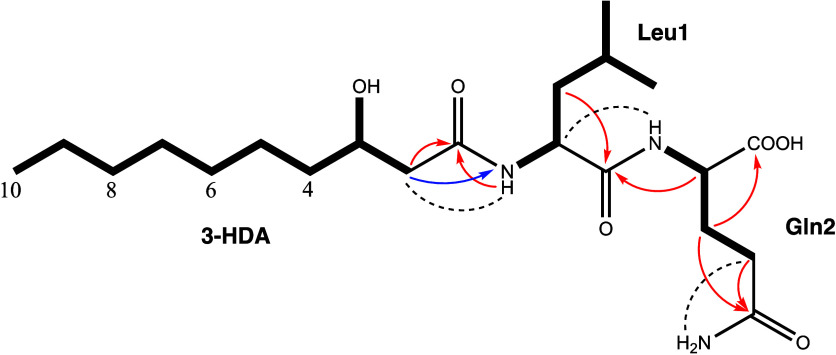
Key NMR correlations of **2**. Bold lines indicate spin systems, determined by the interpretation of COSY or HSQC-TOCSY spectra, while arrows represent either ^1^H–^13^C (red) or ^1^H–^15^N (blue) HMBC long-range key correlations, respectively. Dashed lines show key NOESY cross correlations.

Compound **2** was isolated as a yellow, amorphous solid. Its molecular formula was determined as C_21_H_39_N_3_O_6_ by HRESIMS, which indicates 4 degrees of unsaturation. The ^1^H NMR spectrum revealed amide proton signals (δ_H_ 6.76–8.13, Table 1), amino acid α-proton signals (δ_H_ 4.12–4.34) and an envelope of methylene protons at δ_H_ 1.24 in combination with a triplet resonance at δ_H_ 0.87, suggestive of its lipo-peptidic nature. This inference was further supported by the observation of amino acid Cα carbon signals (δ_C_ 50.6–51.5) and amide carbonyl carbon signals (δ_C_ 170.8–173.5) in the ^13^C NMR spectrum as well as absorption bands for amide carbonyls (3290, 1650, 1540 cm^–1^) in the IR spectrum. Analysis of the 2D NMR spectra, including HSQC, COSY, HMBC, and NOESY, allowed the identification of two proteinogenic amino acid residues Leu and Gln (Figures 1 and S7–S17). The presence of a Gln residue was also supported by the observation of two separate signals for its side chain amide in the ^1^H NMR spectrum (Table 1), which represent the typical *cis–trans* isomers aroused by the partial double-bond character of the carboxamide bond.^25^ Subtraction of the C, N, and O atoms, assigned already for the two identified amino acid residues, from the molecular formula of **2** showed that the remaining lipid portion had to consist of C_10_H_19_O_2_ which could be further dissected by analysis of multiplicity-edited HSQC NMR data into 1× CH_3_, 7× CH_2_, 1× CH–OH, 1× CO. Correlations in the COSY and the HSQC-TOCSY spectra delineated a continuous spin system for protons from H_2_-2 through H_3_-10, while the observed long-range ^1^H–^13^C coupling between H_2_-2 and C-1 subsequently identified a 3-fatty acid fragment as 3-hydroxy decanoic acid (3-HDA) (Figure 1).

**Table 1:** NMR Spectroscopic Data for Salamandamide A (2) and B (5) in *d*_6_-DMSO

Salamandamide A (**2**)^a^				Salamandamide B (**5**)^b^			
unit	position	δ_H_, mult. [*J*, (Hz)]	δ_C/N_, mult.	unit	position	δ_H_, mult. [*J*, (Hz)]	δ_C/N_, mult.
HDA	1		170.8, C	HDA	1		170.8, C
	2	2.20, m	43.5, CH_2_		2	2.19, dd	43.6, CH_2_
	3	3.76, brs	67.5, CH		3	3.77, m	67.5, CH
	4	1.34, m	36.9, CH_2_		4	1.34, m	36.8, CH_2_
	5	1.22, m	25.1, CH_2_		5	1.22, m	25.2, CH_2_
		1.34, m				1.35, m	
	6	1.24, m	29.1, CH_2_		6	1.24, m	29.1, CH_2_
	7	1.24, m	28.7, CH_2_		7	1.24, m	28.7, CH_2_
	8	1.24, m	31.32, CH_2_		8	1.23, m	31.1, CH_2_
	9	1.24, m	22.1, CH_2_		9	1.26, m	22.1, CH_2_
	10	0.86, t	14.0, CH_3_		10	0.86, t	14.0, CH_3_
	3-OH	4.57, d (3.2)			3-OH	4.36, brs	
Leu1	α	4.34, m	50.6, CH	Leu1	α	4.29, m	50.7, CH
	β	1.43, m	40.9, CH_2_		β	1.43, m	40.7, CH_2_
	γ	1.63, m	24.0, CH		γ	1.62, m	24.1, CH
	δ	0.84, m	21.6, CH_3_		δ	0.83, d (7.0)	21.6, CH_3_
	ε	0.88, m	23.1, CH_3_		ε	0.87, d (7.1)	23.1, CH_3_
	C=O		172.3, C		C=O		171.9, C
	NH	7.87, d (8.3)	123.8, NH		NH	7.94, d (7.2)	124.2, NH
Gln2	α	4.12, m	51.5, CH	Glu2	α	4.11, m	51.3,^c^ CH
	β	1.78, m	26.7, CH_2_		β	1.81, m	26.6,^c^ CH_2_
		1.93, m				1.87, m	
	γ	2.12, m	31.26, CH_2_		γ	2.24, m	30.4,^c^ CH_2_
	COOH	12.50, brs	173.2, C		COOH	12.50, brs	173.1, C
	NH	8.13, d (7.6)	117.3, NH		NH	7.94, d	117.8 NH
	C=O_γ_		173.5, C		C=O_γ_		174.0, C
	NH_2_	6.76, s (*cis*)	108.7, NH				
		7.24, s (*trans*)					

a NMR spectra were obtained at 400 (^1^H) and 100 (^13^C) MHz, respectively.

b NMR spectra were obtained at 700 (^1^H) and 175 (^13^C) MHz, respectively.

c Shift value was hardly observable in the ^13^C NMR experiment (S/N ratio 1:1) but extractable from the corresponding 2D NMR experiments.

The sequence of the single components was delineated by ^1^H–^13^C-HMBC cross correlations (Gln-Hα/Leu-CO and Leu-NH/3-HDA-CO) and corroborated by ^1^H–^15^N-HMBC (Leu-N/3-HDA-H_2_-2), ^1^H–^1^H-NOESY (Gln-NH/Leu-Hα and Leu-NH/3-HDA-H_2_-2) and MS/MS data. The assigned residues accounted for the required four double bond equivalents; thus, **2** had to be linear.

**Figure 2:**
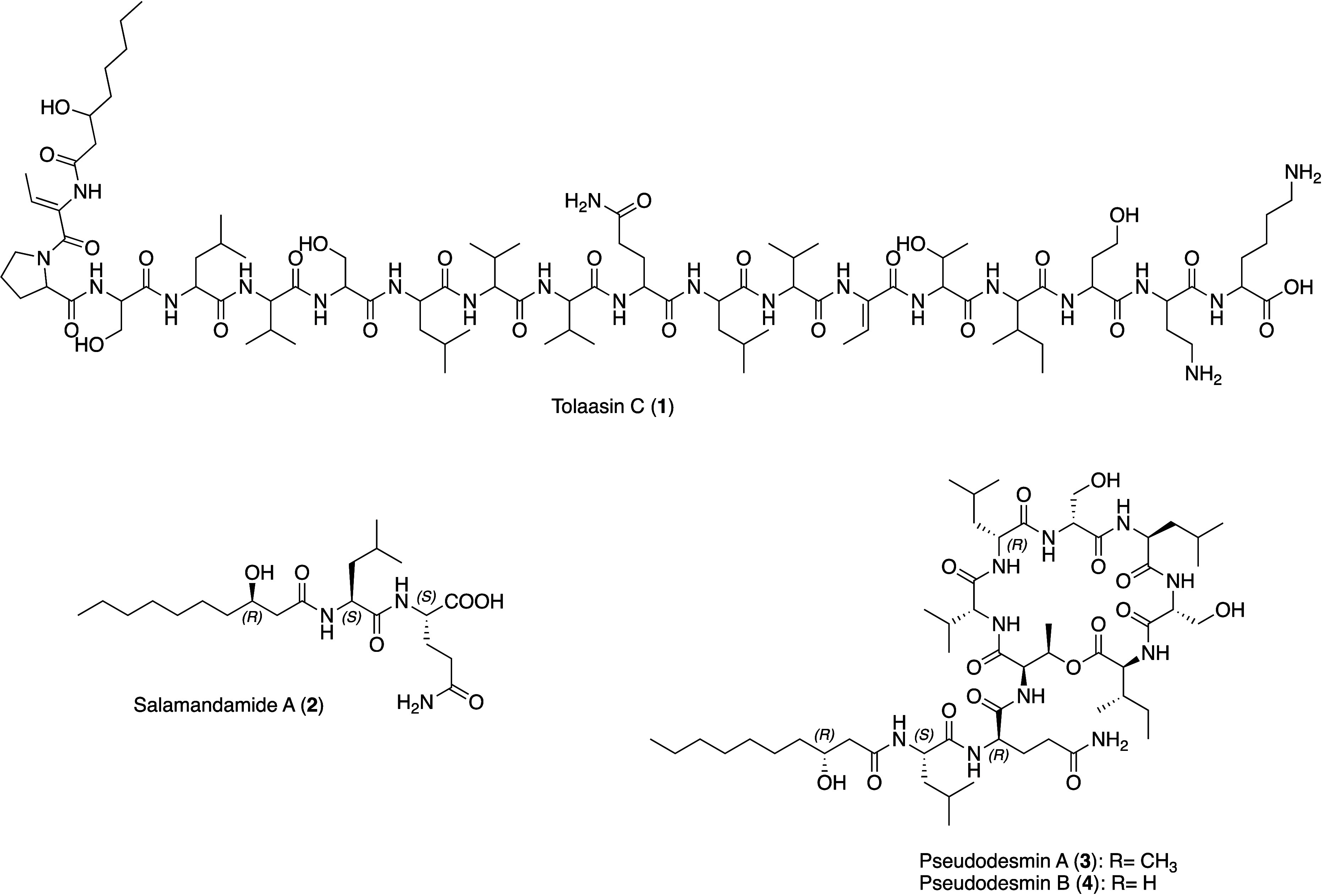
Chemical structures, detected and obtained during this study from *Pseudomonas tolaasii* RSB 5.11.

After the constitution of **2** was known, the absolute configurations of the amino acids and those at C-3 of the lipid side chain were determined. Analysis of the absolute configuration of the chiral 3-HDA residue of **2** was accomplished by enantioselective LC-QTOF-ESI-MS/MS with data-dependent acquisition using a Chiralpak IA-U column employing gradient elution with acetonitrile as published in detail elsewhere.^26^ Regarding the absolute configuration of the 3-hydroxy-decanoic acid, the *R*-configuration dominated, while the *S*-configuration was present at much lower concentration (*R*:*S*-enantiomeric ratio was 80:20; Figure S18). The amino acid composition and configurations were determined by LC-QTOF-ESI-MS/MS with data-dependent acquisition using a Chiralpak QN-AX column as previously described^27^ by comparison of the retention times and mass spectra of the 6-aminoquinolyl-*N*-hydroxysuccinimidyl carbamate (AQC) derivatized hydrolysate of the dipeptide sample **2** with those of authentic standards (Figure S18) and found to be 1 l-Leu and 1 l-Gln (detected as l-Glu as the side chain amide was also converted to a carboxylic group during peptide hydrolysis). The resultant complete 3D structure of **2** is shown in Figure 2. Compound **2** represents the smallest representative of *Pseudomonas*-lipopeptides for which the trivial name salamandamide A is suggested. Natural linear lipo-dipeptides are in general scarce in the field of natural products and to the best of our knowledge, only the linear lipo-dipeptides gageotetrin A (l-Leu-l-Glu-3-OH-11-Me-tridecanoic acid) from *Bacillus subtilis*,^28^ svalbamides (3-amino-2-pyrrolidinone-d-Val-3-OH-8-Me-decanoic acid) from *Paenibacillus* sp.^29^ and barnesins (vinylogous l-Arg-l-Tyr-*trans*-2-octenoic acid) from *Sulfurospirrilum barnesii*(30) have been reported. In the *Pseudomonas* field, lipopeptides are usually classified into families composed of lipopeptides having close chemical structures in terms of length and composition of the peptide moiety and, in the case of a cyclic version, the number of amino acids that form the lactone ring. Salamandamide A does not fall into any of the already established groups of *Pseudomonas*-derived lipopeptides^2^ and thus represents the founding member of a new class of linear lipo-dipeptides.

The identity of compounds **3** and **4** was validated by NMR and HR-MS/MS analyses and compared with literature data (Tables S1 and S5, Figures S20–S25 and S30–S33). Particular attention was hereby paid to the Leu5^1^H^α^ chemical shift since it can be used to dissect between the L- and D-subform of lipo-nonapeptides.^24c^ For viscosinamides A and B, in *d*_3_-MeCN a value of 3.66 and 3.67 ppm, respectively, can be observed for this α-proton, while in the same solvent, this value is shifted downfield to 3.97 ppm for pseudodesmins A and B.^24b,24c^ Since for both isolated lipo-nonapeptides the characteristic downfield shifted resonances for Leu5^1^H^α^ were detected (Tables S1 and S5), they were unambiguously identified as pseudodesmin A (**3**) and B (**4**), respectively. Concerning the lipid portion of **3**, a chiral analysis was initiated which confirmed that its 3-hydroxy-decanoic acid was, likewise in compound **2**, mainly *R*-configured (*R*:*S*-enantiomeric ratio was 95:5; Figure S26). In addition, slow evaporation of compound **3** in MeCN provided single crystals suitable for X-ray analysis (Figures S27–S29). The latter showed that the structure of **3** was in agreement with the known structure of peudodesmin A (CCDC 685601) and thus further corroborated the absolute configuration of **3**.

### Biological Activity

Natural lipo-dipeptides were so far reported to possess protease inhibitory, antimicrobial and chemopreventive activity,^28−30^ whereas synthetically prepared lipo-dipeptides showed emulsifying,^31^ elastin-production stimulating^32^ and antiviral (influenza virus H1N1 and murine CoV) properties.^33^ Consequently, we tested salamandamide A (**2**) in protease and antiviral assays and further evaluated its cytotoxic, antibacterial, and antifungal properties. However, in all applied assay systems, **2** was found to be inactive up to the highest concentration tested (see Tables S6, S7 and Figures S46, S47). Pseudodesmin A (**3**) is known to support swarming of *P. tolaasii* strains and contributes in this way to the mobility of the strain.^34^ Beside this physiological task, it has been reported to possess antiviral activities (HIV1 and VZV) and moderate antimicrobial activity while no cytotoxicity was observed.^20,24b,24c,35^ Concerning a possible antiviral effect, we tested pseudodesmin A (**3**) in an in vitro cell-based assay using a SARS-CoV-2-mNG reporter virus. Caco-2 cells were simultaneously infected and treated with increasing concentrations of **3** and remdesivir as a positive control. Pseudodesmin A was able to reduce the infection rate by 60% in concentrations ranging from 2.5 to 10 μM. Cell counts after infection displayed no toxicity signal up to the highest concentration tested (Figure S46). Notably, when Caco-2 cells were treated with different concentrations of pseudodesmin A (**3**) 2.5 h before infection, it reached 85–100% reduction of the infection rate when applied between 5 to 10 μM. Similarly, as before, no signs of toxicity were observed up to 10 μM (Figure S47). These data indicate that the compound might act on the level of virus attachment or entry. Using a 4-parameter nonlinear regression, EC_50_ values were calculated. An EC_50_ value of 3.3 μM and a CC_50_ value higher than 20 μM further supported a specific antiviral effect of compound **3** in the absence of cellular cytotoxicity in this system.

### Identification of a Single Gene Cluster Responsible for the Biosynthesis of Salamandamide A (2) and Pseudodesmin A (3)

**Figure 3:**
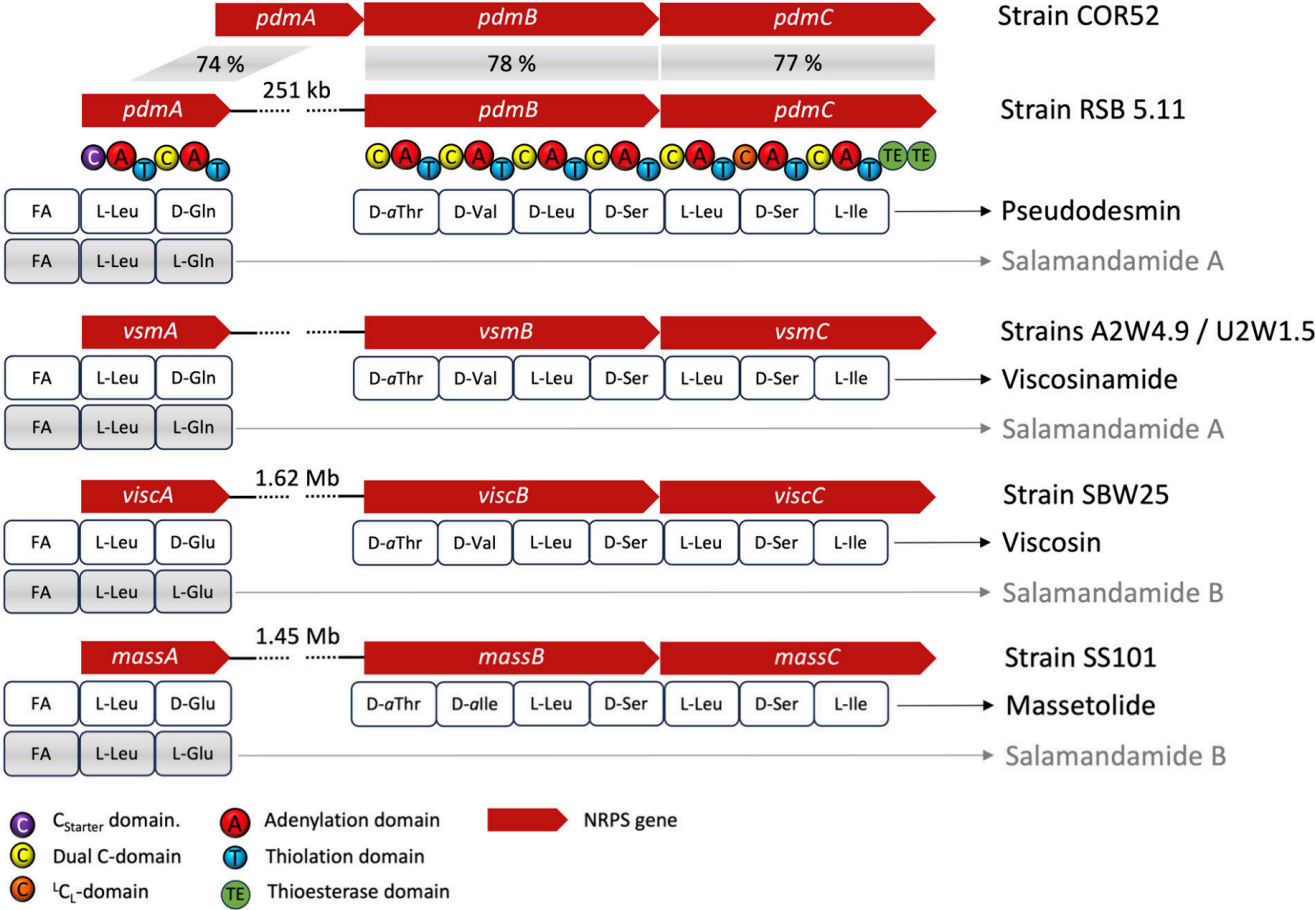
Organization of pseudodesmin gene clusters of *Pseudomonas* sp. COR52 and *P. tolaasii* RSB 5.11, the viscosinamide gene cluster of *Pseudomonas* sp. A2W4.9 and U2W1.5, the viscosin BGC of *P. fluorescens* SBW25 and the massetolide BGC of *P. lactis* SS101 are indicated by red arrows. Below the red structural genes is the predicted lipopeptide sequence depicted in rounded rectangles. Exemplary, underneath the *pdm* gene cluster, additionally the domain organization of the resultant nonribosomal peptide synthetases PdmA and PdmBC are shown.

Upon the chemical identification of **2** as a novel lipo-dipeptide, we aimed to correlate the compound with its biosynthetic gene cluster. We anticipated a bimodular NRPS coding for the domain sequence C_S_-A-T-^L^C_L_-A-T-TE. Such NRPSs were already known from the brabantamide and pyrrolizixenamide gene cluster; however, due to the presence of further accessory monooxygenases,^36^ the initially formed lipo-dipeptides were further transformed into the more complex bicyclic structures, and thus never led to linear lipo-dipeptides. Therefore, we sequenced the complete genome of strain RSB 5.11 using nanopore sequencing technology and utilized the data 2-fold: To clarify the taxonomic position of the strain, and to search for the biosynthetic gene cluster for salamandamide A. A genome-based taxonomic analysis employing the Type Strain Genome Server (TYGS),^37^ revealed that *Pseudomonas tolaasii* NCPPB 2192 represents the closest related type strain. In pairwise comparisons, independent of the applied Genome BLAST Distance Phylogeny formula, the digital DNA–DNA hybridization (dDDH) values *d*_0_, *d*_4_, and *d*_6_ ranged from 94.2 to 96.2% and were therefore well above the species threshold of 70%. With the production of tolaasin lipopeptides, which are typically produced by *P. tolaasii* strains, the bioinformatic finding was in good agreement also from a chemo-taxonomical perspective. Thus, the *Pseudomonas* sp. RSB 5.11 represents an *P. tolaasii* strain. Automated secondary metabolism analysis using antiSMASH v6.0.1^38^ predicted 13 biosynthetic gene clusters. Concerning NRPS-based BGCs, a fragin, a pyoverdin (split-BGC: trimodular NRPS for chromophore assembly and a decamodular NRPS for the peptide chain), the pseudodesmin (split-BGC: consisting of a bi- and a heptamodular NRPS) and the octadecamodular tolaasin BGC could be identified. It is noteworthy to mention that the pseudodesmin (*pdm*) gene cluster of strain *P. tolaasii* RSB 5.11 (Figure 3) represents a bipartite NRPS BGC, in contrast to the first reported pseudodesmin BGC from the *Pseudomonas* sp. COR52 (accession number MT577358).^20^

Alternative biosynthetic machineries, enabled to produce peptides, such as RiPPs,^39^ CDPS-^40^ or ATP-Grasp-based^41^ systems were furthermore considered but were either absent in the genome or did not provide a genetic blueprint that matched the structure of salamandamide A (**2**). Due to the absence of a bimodular NRPS BGC, solely devoted to salamandamide A production, we hypothesized that **2** can be either a degradation product of pseudodesmin A (**3**) or that **2** represents a biosynthetic intermediate and simply accumulated e.g. due to a less efficient work of PdmB, which is then possibly released by the editing function of TE type II of PdmC or alternatively, if *pdmB* is not coordinatively transcribed at the same time or the transfer of the growing peptide chain from PdmA to PdmB is inefficient.

The deduction of the absolute configuration of l-Leu1-l-Gln2 of the peptide portion of **2** proved to be pivotal. It contradicts **2** to be a degradation product, since in **3** a l-Leu1-d-Gln2 configuration is given (Figure 2). Moreover, it supports the *de novo* formation of **2** with the established l-Leu1-l-Gln2 configuration through the action of PdmA, without PdmB being involved, since the first C/E-domain of PdmB cannot epimerize l-Gln anymore. We further figured, in the case of a stalled or less efficient PdmB, if, besides lipo-dipeptides, further biosynthetic intermediates have been released along the NRPS assembly line leading to **3**, specifically from modules 3 through 8. However, the interrogation of the HR-MS-data showed that no linear lipo-tripeptides to lipo-octapeptides could be detected. In summary, it appears that only between PdmA and PdmB a particular biosynthetic breaking point is given. In summary, it appears that only between PdmA and PdmB a particular biosynthetic breaking point is given, that could possibly be caused by issues as e.g. coexpression, compatibility or colocalization of PdmB.

**Figure 4:**
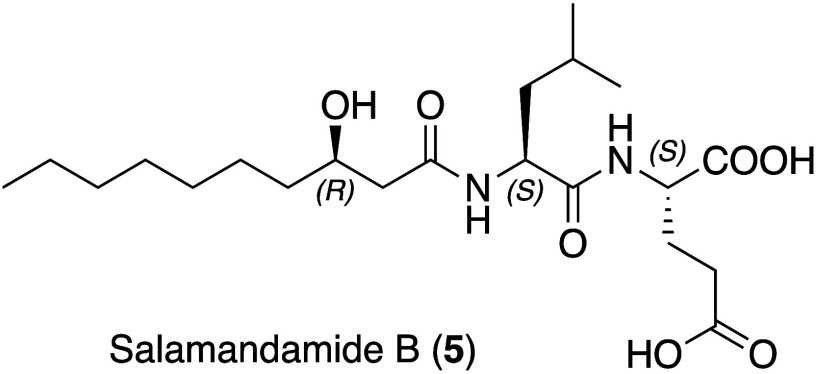
Chemical structure of salamandamide B, obtained from *Pseudomonas lactis* SS101.

This also raised the question if this observation represents an exception and is only given in strain RSB 5.11 or if this also applies (a) for the original nonbipartite pseudodesmin BGC of *Pseudomonas* sp. COR52 and (b) for further *Pseudomonas* species whose genome harbors a split gene cluster encoding lipo-nonapeptides, such as the viscosin, the massetolide, or the viscosinamide BGCs. We thus chemically investigated the corresponding producer strains for the production of lipo-dipeptides. Intriguingly, all tested strains produced beside the expected lipo-nonapeptide also the corresponding dilipopeptide, but in comparison to RSB 5.11 only in minute amounts. Using LC/MS/MS techniques, it could be demonstrated that the viscosinamide-producing strains *Pseudomonas* sp. U2W1.5 and A2W4.9,^20^ as well as the alternative pseudodesmin-producing strain *Pseudomonas* sp. COR52^20^ all biosynthesized salamandamide A (**2**), while the viscosin-producing strain *P. fluorescens* SBW25^17^ and the massetolide-producing strain *P. lactis* SS101^19^ produced, in full agreement with their BGCs, a salamandamide derivative with Glu instead of Gln residue (Figures S34 and S35), which was termed salamandamide B (**5**). Since *P. lactis* SS101 showed, regarding compound **5**, a higher production rate of **5** (0.039 mg/L) than *P. fluorescens* SBW25, a large-scale isolation with strain SS101 was attempted. These efforts led to the isolation of 0.99 mg of salamandamide B (**5**) at 85% purity which allowed a full NMR assignment (Table 1 and Figures S36–S44) and thus corroborated the MS-based results. For biogenetic reasons, we propose that both amino acids are L-configured and that its 3-hydroxy-decanoic acid was, likewise in compounds **2** and **3**, mainly *R*-configured (Figure 4).

Since this biosynthetic phenomenon applied also for the viscosin (*visc*) BGC and systematic knockout mutants were available in strain *P. fluorescens* SBW25,^17^ we revisited the biosynthetic questions that arose at the begin of the study. Chemical analysis of the Δ*viscA* mutant showed that the production of **5** was abolished, while mutant strains Δ*viscB* and Δ*viscC* were still able to synthesize salamandamide B (Figure S45). In any case, compound **5** represents a shunt product of the viscosin pathway. However, the assembled lipodipeptide bound to the T-domain of module 2 has to be released from ViscA, by a type I or type II thioesterase. In the case of the Δ*viscB* mutant, it can be envisioned that the tandem TE domain in ViscC, which consists of a TE-I and a TE-II, can still take over this task, whereas in the Δ*viscC* mutant, this must be mediated by a thioesterase that is encoded elsewhere in the genome.

In summary, we report the discovery of salamandamide A and B and demonstrate that salamandamides and lipo-nonapeptides are biosynthesized from a single biosynthetic gene cluster of the viscosin family. The production yield is strain specific, but the production itself is independent of the genetic architecture of the gene cluster, i.e. if it is a bipartite or a continuous BGC (*pdm* BGC of *P.* sp. COR52).

In rare cases, it has been reported that a single bacterial gene cluster can indeed code for the biosynthesis of two structurally different compounds. This “one BGC–many compounds” phenomenon can be explained by module- or domain-skipping, off-PKS enzymatic processing or simply by accumulation of shunt products in the biosynthesis assembly line.^42a−42h^ In the present case, salamandamides can be considered as incomplete products of the lipo-nonapeptide BGCs. It shares in this way some similarity with dentigerumycin biosynthesis,^42e^ in which the BGC encodes a macrocyclic PKS/NRPS product but also codes for a corresponding smaller linear product, which is due to NRPS module skipping shortened by one amino acid (not seven, like in the case of salamandamides). Whether production can be controlled by the bacterium, e.g. depending on environmental conditions or on the presence of trigger signals, like in the case of the combined bagremycin/ferroverdin BGC,^42f^ currently remains elusive and will be the subject of further studies.

## Experimental Section

### General Experimental Procedures

HPLC was performed with a Waters system, controlled by Waters Millenium Software 4.0 and consisting of a Waters 1525 pump with an integrated degasser, a Waters 2996 photodiode array detector, and a Rheodyne 7725i injector. For LC-MS analysis, an Agilent 1100 Series HPLC system was fitted with a G1312A binary pump, a G1329A autosampler, a G1315A diode array detector, and a Waters In-Line Degasser AF. The HPLC components were connected with an AB Sciex 3200 QTRAP LC/MS/MS mass spectrometer. Liquid Chromatography High-resolution Electron Spray Ionization Tandem Mass Spectrometry (LC-HR-ESI-MS/MS) measurements were performed on a Thermo Scientific UltiMate 3000 HPLC system coupled with a Bruker MaXis-4G mass spectrometer. 1D and 2D NMR spectra were acquired on either a 400 MHz Bruker AVANCE III HD (400, 100, and 40.56 MHz for ^1^H, ^13^C and ^15^N isotopes, respectively) or on a 700 MHz Avance III HDX (700, 175, and 70.97 MHz for ^1^H, ^13^C and ^15^N isotopes, respectively) NMR spectrometer, equipped with a 5 mm broadband SMART or a Prodigy TCI cryo probe head, respectively. All spectra were recorded at 298 or 303 K in either *d*_6_-DMSO (δ_H_/δ_C_ 2.50/39.50) or *d*_3_-MeCN (δ_H_/δ_C_ 1.94/1.32), respectively, and referenced to the residual hydrogenated solvent signals or the internal offset for ^15^N assigned by the instrument manufacturer. NMR spectra were processed and analyzed using MestReNova 14.3.3. Optical rotation values were measured on a Jasco P-2000 polarimeter, using a 3.5 mm × 10 mm cylindrical quartz cell. Infrared spectra were obtained employing a Jasco FT/IR 4200 spectrometer, interfaced with a MIRacle ATR device (ZnSe crystal). UV spectra were measured on a PerkinElmer Lambda 25 UV/vis spectrometer. All solvents were purchased as HPLC or LC-MS grade, respectively.

### Bacterial Strains

*Pseudomonas tolaasii* RSB 5.11 was collected and isolated by one of the authors (C.M.-W.) from the skin swab of a *Plethodon cinereus* salamander obtained from the Shenandoah National Park, USA, VA.^21^*Pseudomonas fluorescens* SBW25 (Sugar Beet Wytham, isolate 25) was originally isolated from the phyllosphere of a sugar beet crop grown at the University Farm, Wytham, Oxford.^43^ The corresponding SBW25 mutant strains Δ*viscA*, Δ*viscB* and Δ*viscC* were kindly provided by Rosanna C. Hennessy who originally obtained them from J. M. Raaijmakers, NIOO-KNAW, Wageningen, The Netherlands.^17^*Pseudomonas* sp. COR52, *Pseudomonas* sp. A2W4.9, *Pseudomonas* sp. U2W1.5 were all isolated from roots of tissue-culture derived cocoyam (*Xanthosoma sagittifolium* L.) plantlets obtained from Cameroon (COR52) or Nigeria (A2W4.9, U2W1.5).^20^*Pseudomonas lactis* SS101 (formerly termed *P. fluorescens* SS101) is a biocontrol strain isolated from wheat rhizosphere in The Netherlands.^44^

### Cultivation and Extraction

The two strains **(***Pseudomonas tolaasii* RSB 5.11 and *Pseudomonas lactis* SS101) were separately precultured in 20 mL of 1% tryptone broth in 50 mL Falcon tubes and incubated for 48 h at 30 °C and shaking at 220 rpm. Compounds **1**-**4** were produced using the following procedure: 3 L Erlenmeyer flasks, each containing 1 L of DMBgly medium (10.6 g Davis Minimal Broth without dextrose, supplemented with 20 mM glycerol), were inoculated with 2 mL of preculture of strain RSB 5.11 and incubated for 72–96 h at 28 °C in an INFORS HT Multitron Pro orbital incubator shaker with shaking at 140 rpm. In several batches, a total volume of 14 L cultivation broth was generated. Subsequently, the whole fermentation broth was twice extracted with BuOH (1:1) using separation funnels to yield 11.6 g crude extract. To produce salamandamide B (**5**), 5 L Erlenmeyer flasks, each containing 1.5 L of DMBgly medium, were inoculated with 3 mL of preculture of strain SS101 and incubated for 72–96 h at 28 °C in an INFORS HT Multitron Pro orbital incubator shaker with shaking at 120 rpm. In several batches, a total volume of 25.5 L cultivation broth was generated. Subsequently, the whole culture was twice extracted with BuOH (1:1) and concentrated *in vacuo* to furnish 4.05 g of the crude extract.

### Purification of Compounds 2–5

The dry extract of strain RSB 5.11 was fractionated by using vacuum liquid chromatography (VLC). The reversed phase column (Macherey-Nagel Polygoprep 60–50 C18) was eluted stepwise under vacuum with solvents of increasing elution strength, ranging from a mixture of 10:90 MeOH-H_2_O to pure methanol to give 6 fractions. LC-MS profiling of these fractions for the target masses for **2** and **3** indicated the fractions, eluting at 40% MeOH in H_2_O and at 100% MeOH, to be of further interest. The 40% fraction was subjected to semipreparative RP-HPLC (gradient 0–5 min 10–35% MeCN; 5–10 min 35–40% MeCN, 10–16 min 40–47% MeCN, 16–17 min 47–100% MeCN, followed by isocratic elution at 100% MeCN for an additional 2 min, Waters Symmetry column, 4.6 mm × 250 mm, 1 mL/min and UV detection at 210 nm) using HPLC-grade MeCN (Honeywell/Riedel-de Haen) and Milli-Q H_2_O (0.1% TFA). For the 100% MeOH fraction, the following conditions were applied: gradient 0–5 min 20–30% MeCN, 5–10 min 30–70% MeCN, 10–15 min 70–90% MeCN, 15–20 min 90–100% MeCN, followed by isocratic elution at 100% MeCN for an additional 1 min, using HPLC-grade MeCN and Milli-Q H_2_O (0.1% TFA), a Phenomenex Kinetex EVO C18, 5 μm column, 4.6 × 250 mm, operated at 1 mL/min and UV detection at 210 nm. This afforded compound **2** (7.0 mg) from the 40% fraction and compound **3** (120.1 mg) and **4** (7.6 mg) from the 100% fraction, respectively. In order to obtain compound **5**, the crude extract of strain SS101 was loaded on a C_18_ VLC column (Macherey-Nagel Polygoprep 60–50 C18) and fractionated sequentially with 500 mL aliquots of 10%, 30%, 40%, 50%, 60%, 70% and 100% MeOH in H_2_O to yield 6 fractions. Salamandamide B (**5**) was detected in the 40% MeOH-H_2_O fraction. The latter fraction was subjected to RP-HPLC (Phenomenex Luna C18(2), 5 μm column, 10 × 250 mm, operated at 2.5 mL/min and UV detection at 210 nm) under gradient solvent conditions (0–2 min 10% MeCN, 2–28 min 10–100% MeCN, followed by isocratic elution at 100% MeCN for additional 3 min, using HPLC-grade MeCN and Milli-Q H_2_O (0.05% TFA)) and yielded 6 subfractions. The last thereof was further purified using the same RP material but a different column diameter (Phenomenex Luna C18(2), 5 μm column, 4.6 × 250 mm, operated at 0.9 mL/min and UV detection at 210 nm) and an optimized gradient solvent system (0–2 min 25% MeCN, 2–25 min 25–80% MeCN, 25–28 min 100% MeCN) which resulted in 8 subfractions. The last thereof was rechromatographed using the same column and gradient solvent system mentioned above to obtain 0.99 mg of **5** at 85% purity.

### Determination of the Absolute Configurations of the Chiral Constituents of 1

LC-ESI-QTOF-MS measurements were performed with an Agilent 1290 Infinity UHPLC system (Agilent Technologies, Waldbronn, Germany) consisting of a binary pump, thermostated column compartment, and CTC-PAL HTS autosampler (CTC Analytics, Zwingen, Switzerland) hyphenated with a TripleTOF 5600+ MS instrument from Sciex (Ontario, Canada) with Duospray Ion Source operated in electrospray ionization mode. Data were acquired by information-dependent acquisition (IDA).

For the 3-HDA, the negative ion mode with the following instrument settings was used: curtain gas (CUR) 30 psi, ion source gas (nebulizing gas; GS1) 50 psi, heater gas (drying gas; GS2) 40 psi, ion spray voltage floating (ISVF) 4500 V, source temperature (TEM) 450 °C. Each MS cycle consisted of a TOF-MS full scan experiment scan in the range of *m*/*z* 100–2000 with a collision energy (CE) of −10 V, declustering potential −100 V and an accumulation time of 250 ms. For the subsequent IDA MS/MS experiments with dynamic background subtraction, the top 5 abundant ions were selected for fragmentation using a CE of −30 V, a DP of −100 V, CAD 6.0, and an accumulation time of 100 ms were used. 3-OH-FA separation (Figure S18A) was performed on a CHIRALPAK IA-U column (100 × 3.0 mm, 1.6 μm). The mobile phases comprised water (MP-A) and acetonitrile (MP-B), both containing 0.1% (v/v) acetic acid. The following gradient was employed: 0–2 min 10% MP-B, 2–20 min 10–100% MP-B, 20–22 min 100% MP-B, 22–22.1 min 100–10% MP-B, and 22.1–25 min 10% MP-B. The flow rate was set to 300 μL/min. For the analysis of the hydrolysate of compound **2**, the temperature was set to 30 °C and the injection volume was set to 3 μL, while for the hydrolysate of compound **3**, the temperature was set to 40 °C and the injection volume was set to 10 μL.

The racemic (±)-3-hydroxydecanoic acid standard was purchased from Sigma-Aldrich (Merck, Taufkirchen, Germany). Rhamnolipid (R-95), dirhamnolipid dominant (Rha), was also obtained from Sigma-Aldrich (Merck). The rhamnolipid (R-95) was hydrolyzed to yield (*R*)-3-hydroxydecanoic acid (along with (*R*)-3-hydroxyoctanoic, -dodecanoic and -tetradecanoic acids), which was used as standard to assign the absolute configuration. For rhamnolipid hydrolysis, 5 mg of rhamnolipid (R-95) were dissolved in 0.5 mL MeOH. 50 μL of this solution were mixed with 50 μL of a methanolic solution of 2N NaOH and the reaction mixture diluted with 900 μL of a solution of THF/MeOH (9:1, v/v). The reaction mixture was stirred for 2 h at room temperature (≈ 25 °C). The solvents were then removed under vacuum, and the residue was diluted with 200 μL of water and acidified with 0.1 M HCl to pH 2–3. Subsequently, the solution was extracted three times with 200 μL of ethyl acetate. The combined organic layers were evaporated to dryness and reconstituted with 100 μL MeOH/H_2_O (3:7, v/v). Prior to injection, the solution was diluted 10-fold (H_2_O) for LC-MS analysis.

For the AQC-derivatized amino acid the positive ion mode with the following instrument settings was used: curtain gas (CUR) 40 psi, ion source gas (nebulizing gas; GS1) 60 psi, heater gas (drying gas; GS2) 60 psi, ion spray voltage floating (ISVF) 5500 V, source temperature (TEM) 450 °C. Each MS cycle consisted of a TOF-MS full scan experiment scan in the range of *m*/*z* 30–2000 with a collision energy (CE) of 10 V, declustering potential 100 V and an accumulation time of 250 ms. For the subsequent IDA MS/MS experiments with dynamic background subtraction, the top 10 abundant ions were selected for fragmentation using a CE of 45 V, a CES 15 V, a DP of 100 V, CAD 6.0, and an accumulation time of 100 ms were used. Amino acid separation (Figure S18B) was performed on a CHIRALPAK QN-AX column (150 × 4.6 mm, 5 μm), employing the following gradient: 0–12 min 0–100% MP-B, 12–45 min 100% MP-B, 41–45.1 min 100–0% MP-B, 45.1–60 min 0% MP-B. The flow rate was set to 135 μL/min (300 μL/min for re-equilibration), the temperature to 30 °C and the injection volume to 1 μL. Further chiral amino acid separations (Figure S18C) were conducted on a CHIRALPAK QN-AX column with a smaller diameter (150 × 2.1 mm, 5 μm). The following gradient was applied: 0–4 min 0–100% MP-B, 4–15 min 100% MP-B, 15–16 min 100–0% MP-B, 16–25 min 0% MP-B. The flow rate was set to 650 μL/min, the temperature to 30 °C and the injection volume to 3 μL. In both cases mobile phases were MP-A: 50 mM NH_4_FA (pH5) and MP-B: 100 mM NH_4_FA (pH6).

### X-ray Crystal Structure Analysis of Pseudodesmin A (3)

A colorless crystal (0.140 × 0.250 × 0.320 mm^3^) was analyzed on a Stadi VARI diffractometer equipped with microfocus sealed Cu Kα X-ray tube radiation and a Dectris Eiger2 CdTe 1M-detector. The sample was extracted under inert oil from a microscope slide and then mounted on a LithoLoops before being flash cooled to 120 K using an Oxford Cryosystems Cryostream 700 open-flow N2 cooling device. Unit cell measurement, data collection and data reduction were performed using the software X-AREA (STOE & CIE GmbH Darmstadt, Germany). The raw data were scaled using LANA (STOE) The structure was solved using SHELXT and refined using SHELXL.

### Salamandamide A (2)

Amorphous, white powder; [α]_D_^23^ −23.2 (c 1.035, MeOH); UV (MeOH) λ_max_ (log ε): 197 (end absorption) nm; FT-IR (ATR) ν_max_ 3290, 2960, 2930, 2860, 1650, 1540, 1200 cm^–1^; ^1^H NMR and ^13^C NMR data, see Table 1; positive HRESIMS *m*/*z* 430.2915 [M + H]^+^ (calcd for C_21_H_40_N_3_O_6_, 430.2922, Δ = −1.6 ppm).

### Pseudodesmin A (3)

White powder; UV (MeOH) λ_max_ (log ε): 197 (end absorption) nm; FT-IR (ATR) ν_max_ 3320, 2960, 2930, 2870, 1750, 1650, 1520, 1460, 1280, 1070 cm^–1^; ^1^H NMR and ^13^C NMR data, see Table S1; positive HRESIMS *m*/*z* 1125.71290 [M + H]^+^ (calcd for C_54_H_97_N_10_O_15_, 1125.71294, Δ = −0.03 ppm).

### Pseudodesmin B (4)

White powder; UV (MeOH) λ_max_ (log ε): 197 (end absorption) nm; ^1^H NMR and ^13^C NMR data, see Table S2; positive HRESIMS *m*/*z* 1111.6993 [M + H]^+^ (calcd for C_53_H_95_N_10_O_15_, 1111.6973, Δ = +1.8 ppm).

### Salamandamide B (5)

White powder; ^1^H NMR and ^13^C NMR data, see Table 1; positive HRESIMS *m*/*z* 431.2759 [M + H]^+^ (calcd for C_21_H_39_N_2_O_7_, 431.2763, Δ = −0.9 ppm).

### Genome Sequencing of *Pseudomonas tolaasii* RSB 5.11

The strain was grown on an LB agar plate from a single bacterial colony. Genomic DNA (gDNA) extraction incorporated cell lysis by enzymatic digestion using lysozyme followed by proteinase K digestion. To reduce DNA shear and maximize throughput, a magnetic bead-based extraction was subsequently performed. Finally, the gained gDNA was dissolved in Tris buffer and checked using an Agilent 4200 TapeStation System and Qubit 3.0 Fluorometer. A yield of 11900 ng was obtained, which exhibited a DNA Integrity Number (DIN) of 8. The library was constructed using a Ligation Sequencing (SQK-LSK109) and a Barcoding (EXP-NBD104) Kit. Sequencing was performed on one Oxford Nanopore GridION flowcell FLO-MIN106. The obtained data were base called using Guppy version 5.0.16 with the “super accurate” basecalling mode, and adapters were trimmed. Sequencing yielded 280260 reads with a median read length of 12607 nt (N_50_: 18021 nt). Contigs were assembled *de novo* and corrected using Flye 2.9^45^ and polished based on ONT reads using Medaka 1.4.3 (https://github.com/nanoporetech/medaka). Overall, the reads were assembled into a 6.37 Mbp nucleotide draft genome at a 555-fold coverage. The resulting sequence consists of solely one contig with a G+C content of 61%. Gene functional annotation using PGAP 6.6 identified 5541 coding genes.

### Protease Inhibition Assays

The main protease of SARS-CoV-2 (M^pro^) and human cathepsin L, both cysteine proteases, as well as human leukocyte elastase, a serine protease, were assayed with fluorogenic and chromogenic peptide substrates, respectively, as previously described.^46^ The formation of 7-amino-4-methylcoumarin (AMC), and *para*-nitroaniline (pNA) respectively, was followed over 10 min (M^pro^, leukocyte elastase) or 60 min (cathepsin L).

### Antiviral Assay

Human colorectal adenocarcinoma cells (Caco-2, ATCC HTB-37, ATCC Manassas, VA, USA) were cultured in Dulbecco′s modified Eagle′s medium (DMEM + GlutaMAX-I, Gibco, Thermo Fisher Scientific, Dreieich, Germany) supplemented with 10% Fetal Bovine Serum (FBS, Gibco), 1% nonessential amino acids (NEAAs, Gibco), and 1% penicillin/streptomycin (Sigma-Aldrich) and were maintained at 37 °C and 5% CO_2_. All experiments associated with SARS-CoV-2 were conducted in a Biosafety Level 3 laboratory. The recombinant infectious SARS-CoV-2 clone expressing mNeonGreen (icSARS-CoV-2-mNG)^47^ was obtained from the World Reference Center for Emerging Viruses and Arboviruses at the University of Texas Medical Branch, Galveston, TX, USA. To test the lipopeptide′s antiviral activity a total of 1 × 10^4^ Caco-2 cells were seeded in 96-well plates the day before infection in media containing 5% FBS. Caco-2 cells were infected with icSARS-CoV-2-mNG at a multiplicity of infection (MOI) of 0.26 or mock-infected. Simultaneously, cells were incubated with salamandamide A (**2**), pseudodesmin A (**3**), or remdesivir (RDV) as a positive control at a concentration ranging from 0.31 to 20 μM. 48 h postinfection, the cells were fixed with 2% paraformaldehyde (PFA) and stained with Hoechst dye at a final concentration of 1 μg/mL. Alternatively, 1 × 10^4^ Caco-2 cells seeded in 96-well plates were pretreated with the lipopeptides or RDV at the mentioned concentrations. 2:30 h post-treatment, the cells were infected with icSARS-CoV-2-mNG at a multiplicity of infection (MOI) of 0.26 or mock-infected for 48 h. Fixation and staining were performed identically. Images were taken with Cytation3 (BioTek, Bad Friedrichshall, Germany). Hoechst and mNeonGreen cells were automatically counted by the Gen5 Software (BioTek). Infection rates were calculated as the ratio of infected cells (mNeonGreen-positive) over the total number of cells (Hoechst-positive). GraphPad Prism ver. 9.1.2 was used for statistical analysis. For Figure S45, data come from one biological replicate. For Figure S37, data come from three biological replicates, and the data are plotted with ± SEM. EC_50_ values (μM) were calculated using 4-parameter nonlinear regression (log(inhibitor) vs response-variable slope).

### Antifungal Assay

The minimal inhibitory concentration (MIC) of pure compounds against different *Candida* clinical isolates was determined by broth microdilution using the direct colony suspension method with an inoculum of 0.5–2.5 × 10^5^ CFU/mL, according to the recommendations of the European Committee on Antimicrobial Susceptibility Testing (EUCAST).^48^ Caspofungin was used as a reference antifungal agent. MIC testing was performed in sterile 96-well microdilution plates using MOPS-buffered RPMI 1640 medium supplemented with glucose to a final concentration of 2%, pH 7.0. MICs were read after incubation of the microplates at 37 °C for 48 h in a Tecan M200 microplate reader at 530 nm.

### Antibacterial Assay

The minimal inhibitory concentration (MIC) was determined by broth microdilution in cation-adjusted Mueller-Hinton medium that contains casein, beef extract and starch according to the standards and guidelines of the Clinical and Laboratory Standards Institute (CLSI).^49^ In brief, a 2-fold serial dilution of the test compound was prepared in microtiter plates and seeded using a final test concentration of bacteria of 5 × 10^5^ colony-forming units per mL. After overnight incubation at 37 °C, the MIC was determined as the lowest compound concentration preventing visible bacterial growth. For *Neisseria*, the medium was supplemented with 2.5% fetal bovine serum. The strain panel included representative species of nosocomial pathogens, which are known as “ESKAPE” bacteria. Specifically, the following strains were used: model strain *Bacillus subtilis* 168, reference strains *Staphylococcus aureus* ATCC 29213, *Enterococcus faecalis* ATCC 29212, *Escherichia coli* ATCC 25922, *Escherichia coli* C600 Tn10 Δ*lpxC* Δ*tolC*, *Klebsiella pneumoniae* ATCC 12657, *Enterobacter aerogenes* ATCC 13048, *Pseudomonas aeruginosa* ATCC 27853, *Neisseria gonorrhoeae* ATCC 19424, clinical isolates *Enterococcus faecium* BM 4147 (vancomycin-resistant), *Enterococcus faecium* BM 4147–1 (cured of the vanA plasmid), *Enterococcus faecium* 6011 (vancomycin resistant), *Enterococcus faecium* U200 (vancomycin-resistant), *Enterococcus faecium* 115.2, *Enterococcus faecium* 209.5, *Acinetobacter baumannii* 09987, *Neisseria gonorrhoeae* S1441.

### Cytotoxicity Assay

The cytotoxicity test against the HeLa human cervical carcinoma cell line was performed in RPMI cell culture medium supplemented with 10% fetal bovine serum using the 7-hydroxy-3H-phenoxazin-3-one-10-oxide (resazurin) assay. A 2-fold serial dilution of the test compounds was prepared in duplicates in a microtiter plate and seeded with trypsinized HeLa cells to a final cell concentration of 1 × 10^4^ cells per well. After 24 h of incubation at 37 °C, 5% CO_2_, 95% relative humidity, resazurin was added at a final concentration of 200 μM, and cells were again incubated overnight. Cell viability was assessed by determining the reduction of resazurin to the fluorescent resorufin. Fluorescence was measured in a TECAN M200 reader at an excitation wavelength of 560 nm and an emission wavelength of 600 nm in relation to an untreated control.

## Data Availability

The genome of *Pseudomonas tolaasii* RSB 5.11 has been deposited in GenBank with the bioproject accession number PRJNA1083108. NMR spectra for molecules **2**, **3**, **4** and **5** have been deposited to the NP-MRD database (NP0333622, NP0333713, NP0333718 and NP0350902, respectively). Crystallographic data for the structure of compound **3** reported in this paper have been deposited with the Cambridge Crystallographic Data Centre (CCDC 2380284). Copies of the data can be obtained, free of charge, on application to the Director, CCDC, 12 Union Road, Cambridge CB2 1EZ, UK (fax: + 44-(0)1223–336033 or e-mail: deposit@ccdc.cam.ac.uk).

## Supporting Information Available

Crystallographic information (CIF)
Spectroscopic, crystallographic and biological assay data (PDF)

## Supplementary Material

np5c00084_si_001.cif

## References

[ref1] Raaijmakers J. M.; de Bruijn I.; de Kock M. J. D. Cyclic lipopeptide production by plant-associated *Pseudomonas* spp.: diversity, activity, biosynthesis, and regulation. Mol. Plant-Microbe Interact. 2006, 19, 699–710. 10.1094/MPMI-19-0699. 16838783

[ref2] Götze S.; Stallforth P. Structure, properties, and biological functions of nonribosomal lipopeptides from pseudomonads. Nat. Prod. Rep. 2020, 37, 29 10.1039/C9NP00022D. 31436775

[ref3] Girard L.; Höfte M.; De Mot R. Lipopeptide families at the interface between pathogenic and beneficial *Pseudomonas*-plant interactions. Crit. Rev. Microbiol. 2020, 46, 397–419. 10.1080/1040841X.2020.1794790. 32885723

[ref4] Cesa-Luna C.; Geudens N.; Girard L.; De Roo L.; Maklad H. R.; Martins J. C.; Höfte M.; De Mot R. Charting the Lipopeptidome of Nonpathogenic *Pseudomonas*. mSystems 2023, 8, e00988–22. 10.1128/msystems.00988-22. PMC9948697 36719227

[ref5] Geudens N.; Martins J. C. Cyclic Lipodepsipeptides From *Pseudomonas* spp.–Biological Swiss-Army Knives. Front. Microbiol. 2018, 9, 1867 10.3389/fmicb.2018.01867. 30158910 PMC6104475

[ref6] Raaijmakers J. M.; de Bruijn I.; Nybroe O.; Ongena M. Natural functions of lipopeptides from *Bacillus* and *Pseudomonas*: more than surfactants and antibiotics. FEMS Microbiol. Rev. 2010, 34, 1037–1062. 10.1111/j.1574-6976.2010.00221.x. 20412310

[ref7] Oni F. E.; Esmaeel Q.; Onyeka T.; Adeleke R.; Jacquard C.; Clement C.; Gross H.; Barka E. A.; Höfte M. *Pseudomonas* Lipopeptide-Mediated Biocontrol: Chemotaxonomy and Biological Activity. Molecules 2022, 27, 372 10.3390/molecules27020372. 35056688 PMC8777863

[ref8] Süssmuth R. D.; Mainz A. Nonribosomal Peptide Synthesis-Principles and Prospects. Angew. Chem., Int. Ed. 2017, 56, 3770–3821. 10.1002/anie.201609079. 28323366

[ref9] Du L.; Lou L. PKS and NRPS release mechanisms. Nat. Prod. Rep. 2010, 27, 255–278. 10.1039/B912037H. 20111804

[ref10] Roongsawang N.; Washio K.; Morikawa M. Diversity of nonribosomal peptide synthetases involved in the biosynthesis of lipopeptide biosurfactants. Int. J. Mol. Sci. 2011, 12, 141–172. 10.3390/ijms12010141. PMC3039948 21339982

[ref11] aSchwarzer D.; Mootz H. D.; Linne U.; Marahiel M. A. Regeneration of misprimed nonribosomal peptide synthetases by type II thioesterases. Proc. Natl. Acad. Sci. U.S.A. 2002, 99, 14083–14088. 10.1073/pnas.212382199. 12384573 PMC137840

[ref12] Balibar C. J.; Vaillancourt F. H.; Walsh C. T. Generation of D amino acid residues in assembly of arthrofactin by dual condensation/epimerization domains. Chem. Biol. 2005, 12, 1189–1200. 10.1016/j.chembiol.2005.08.010. 16298298

[ref13] Dekimpe S.; Masschelein J. Beyond peptide bond formation: the versatile role of condensation domains in natural product biosynthesis. Nat. Prod. Rep. 2021, 38, 1910–1937. 10.1039/D0NP00098A. 34676836

[ref14] aKraas F. I.; Helmetag V.; Wittmann M.; Strieker M.; Marahiel M. A. Functional dissection of surfactin synthetase initiation module reveals insights into the mechanism of lipoinitiation. Chem. Biol. 2010, 17, 872–880. 10.1016/j.chembiol.2010.06.015. 20797616

[ref15] Li W.; Rokni-Zadeh H.; De Vleeschouwer M.; Ghequire M. G. K.; Sinnaeve D.; Xie G.-L.; Rozenski J.; Madder A.; Martins J. C.; De Mot R. The antimicrobial compound xantholysin defines a new group of *Pseudomonas* cyclic lipopeptides. PLoS One 2013, 8, e62946 10.1371/journal.pone.0062946. 23690965 PMC3656897

[ref16] Vallet-Gely I.; Novikov A.; Augusto L.; Liehl P.; Bolbach G.; Péchy-Tarr M.; Cosson P.; Keel C.; Caroff M.; Lemaitre B. Association of hemolytic activity of *Pseudomonas entomophila*, a versatile soil bacterium, with cyclic lipopeptide production. Appl. Environ. Microbiol. 2010, 76, 910–921. 10.1128/AEM.02112-09. 20023108 PMC2812987

[ref17] de Bruijn I.; de Kock M. J. D.; Yang M.; de Waard P.; van Beek T. A.; Raaijmakers J. M. Genome-based discovery, structure prediction and functional analysis of cyclic lipopeptide antibiotics in *Pseudomonas* species. Mol. Microbiol. 2007, 63, 417–428. 10.1111/j.1365-2958.2006.05525.x. 17241198

[ref18] Rokni-Zadeh H.; Li W.; Sanchez-Rodriguez A.; Sinnaeve D.; Rozenski J.; Martins J. C.; De Mot R. Genetic and functional characterization of cyclic lipopeptide white-line-inducing principle (WLIP) production by rice rhizosphere isolate *Pseudomonas putida* RW10S2. Appl. Environ. Microbiol. 2012, 78, 4826–4834. 10.1128/AEM.00335-12. 22544260 PMC3416372

[ref19] de Bruijn I.; de Kock M. J. D.; de Waard P.; van Beek T. A.; Raaijmakers J. M. Massetolide A biosynthesis in *Pseudomonas fluorescens*. J. Bacteriol. 2008, 190, 2777–2789. 10.1128/JB.01563-07. 17993540 PMC2293227

[ref20] Oni F. E.; Geudens N.; Adiobo A.; Omoboye O. O.; Enow E. A.; Onyeka J. T.; Salami A. E.; De Mot R.; Martins J. C.; Höfte M. Biosynthesis and Antimicrobial Activity of Pseudodesmin and Viscosinamide Cyclic Lipopeptides Produced by Pseudomonads Associated with the Cocoyam Rhizosphere. Microorganisms 2020, 8, 1079 10.3390/microorganisms8071079. 32698413 PMC7409209

[ref21] Muletz-Wolz C. R.; DiRenzo G. V.; Yarwood S. A.; Campell Grant E. H.; Fleischer R. C.; Lips K. R. Antifungal Bacteria on Woodland Salamander Skin Exhibit High Taxonomic Diversity and Geographic Variability. Appl. Environ. Microbiol. 2017, 83, e00186–17. 10.1128/AEM.00186-17. 28213545 PMC5394319

[ref22] aYang J. Y.; Sanchez L. M.; Rath C. M.; Liu X.; Boudreau P. D.; Bruns N.; Glukhov E.; Wodtke A.; de Felicio R.; Fenner A.; Wong W. R.; Linington R. G.; Zhang L.; Debonsi H. M.; Gerwick W. H.; Dorrestein P. C. Molecular networking as a dereplication strategy. J. Nat. Prod. 2013, 76, 1686–1699. 10.1021/np400413s. 24025162 PMC3936340

[ref23] aNutkins J. C.; Mortishire-Smith R. J.; Packman L. C.; Brodey C. L.; Rainey P. B.; Johnstone K.; Williams D. H. Structure determination of tolaasin, an extracellular lipodepsipeptide produced by the mushroom pathogen, *Pseudomonas tolaasii* Paine. J. Am. Chem. Soc. 1991, 113, 2621–2627. 10.1021/ja00007a040.

[ref24] aNielsen T. H.; Christophersen C.; Anthoni U.; So̷rensen J. Viscosinamide, a new cyclic depsipeptide with surfactant and antifungal properties produced by *Pseudomonas fluorescens* DR54. J. Appl. Microbiol. 1999, 87, 80–90. 10.1046/j.1365-2672.1999.00798.x. 10432590

[ref25] Zanacchi R. M.; Moore W. J. Temperature and pH dependence of proton N.M.R. of glutamine in peptides. Aust. J. Chem. 1980, 33, 1505–1510. 10.1071/CH9801505.

[ref26] Karongo R.; Jiao J.; Gross H.; Lämmerhofer M. Direct enantioselective gradient reversed-phase ultra-high performance liquid chromatography tandem mass spectrometry method for 3-hydroxy alkanoic acids in lipopeptides on an immobilized 1.6 μm amylose-based chiral stationary phase. J. Sep. Sci. 2021, 44, 1875–1883. 10.1002/jssc.202100104. 33666325

[ref27] Li F.; Karongo R.; Mavridou D.; Horak J.; Sievers-Engler A.; Lämmerhofer M. Automated sample preparation with 6-Aminoquinolyl-N-hydroxysuccinimidyl carbamate and iodoacetamide derivatization reagents for enantioselective liquid chromatography tandem mass spectrometry amino acid analysis. J. Chromatogr. A 2023, 1708, 464349 10.1016/j.chroma.2023.464349. 37696129

[ref28] Tareq F. S.; Lee M. A.; Lee H.-S.; Lee Y.-J.; Lee J. S.; Hasan C. M.; Islam Md. T.; Shin H. J. Gageotetrins A-C, noncytotoxic antimicrobial linear lipopeptides from a marine bacterium *Bacillus subtilis*. Org. Lett. 2014, 16, 928–931. 10.1021/ol403657r. 24502521

[ref29] Du Y. E.; Bae E. S.; Lim Y.; Cho J.-C.; Nam S.-J.; Shin J.; Lee S. K.; Nam S.-I.; Oh D.-C. Svalbamides A and B, Pyrrolidinone-Bearing Lipodipeptides from Arctic *Paenibacillus* sp. Mar. Drugs 2021, 19, 229 10.3390/md19040229. 33920625 PMC8073366

[ref30] aRischer M.; Raguz L.; Guo H.; Keiff F.; Diekert G.; Goris T.; Beemelmanns C. Biosynthesis, Synthesis, and Activities of Barnesin A, a NRPS-PKS Hybrid Produced by an Anaerobic Epsilonproteobacterium. ACS Chem. Biol. 2018, 13, 1990–1995. 10.1021/acschembio.8b00445. 29901979

[ref31] Lv W.; Hu T.; Taha A.; Wang Z.; Xu X.; Pan S.; Hu H. Lipo-Dipeptide as an Emulsifier: Performance and Possible Mechanism. J. Agric. Food Chem. 2019, 67, 6377–6386. 10.1021/acs.jafc.9b01721. 31117499

[ref32] Oshimura E.; Sakamoto K. Chapter 19, Amino Acids, Peptides, and Proteins. In Cosmetic Science and Technology: Theoretical Principles and Applications; Sakamoto K.; Lochhead R. Y.; Maibach H. I.; Yamashita Y., Eds.; Elsevier: Amsterdam, 2017; pp 285–304.

[ref33] Sardar A.; Lahiri A.; Kamble M.; Mallick A. I.; Tarafdar P. K. Translation of Mycobacterium Survival Strategy to Develop a Lipo-peptide based Fusion Inhibitor. Angew. Chem., Int. Ed. 2021, 60, 6101–6106. 10.1002/anie.202013848. PMC7753697 33241871

[ref34] Hermenau R.; Kugel S.; Komor A. J.; Hertweck C. Helper bacteria halt and disarm mushroom pathogens by linearizing structurally diverse cyclolipopeptides. Proc. Natl. Acad. Sci. U.S.A. 2020, 117, 23802–23806. 10.1073/pnas.2006109117. 32868430 PMC7519232

[ref35] De Vleeschouwer M.; Van Kersavond T.; Verleysen Y.; Sinnaeve D.; Coenye T.; Martins J. C.; Madder A. Identification of the Molecular Determinants Involved in Antimicrobial Activity of Pseudodesmin A, a Cyclic Lipopeptide From the Viscosin Group. Front. Microbiol. 2020, 11, 646 10.3389/fmicb.2020.00646. 32373092 PMC7187754

[ref36] aSchmidt Y.; van der Voort M.; Crüsemann M.; Piel J.; Josten M.; Sahl H.-G.; Miess H.; Raaijmakers J. M.; Gross H. Biosynthetic origin of the antibiotic cyclocarbamate brabantamide A (SB-253514) in plant-associated *Pseudomonas*. ChemBioChem. 2014, 15, 259–266. 10.1002/cbic.201300527. 24436210

[ref37] Meier-Kolthoff J. P.; Göker M. TYGS is an automated high-throughput platform for state-of-the-art genome-based taxonomy. Nat. Commun. 2019, 10, 2182 10.1038/s41467-019-10210-3. 31097708 PMC6522516

[ref38] Blin K.; Shaw S.; Kloosterman A. M.; Charlop-Powers Z.; van Wezel G. P.; Medema M. H.; Weber T. antiSMASH 6.0: improving cluster detection and comparison capabilities. Nucleic Acids Res. 2021, 49, W29–W35. 10.1093/nar/gkab335. 33978755 PMC8262755

[ref39] aWiebach V.; Mainz A.; Siegert M.-A. J.; Jungmann N. A.; Lesquame G.; Tirat S.; Dreux-Zigha A.; Aszodi J.; Le Beller D.; Süssmuth R. D. The anti-staphylococcal lipolanthines are ribosomally synthesized lipopeptides. Nat. Chem. Biol. 2018, 14, 652–654. 10.1038/s41589-018-0068-6. 29915235

[ref40] Gondry M.; Jacques I. B.; Thai R.; Babin M.; Canu N.; Seguin J.; Belin P.; Pernodet J.-L.; Moutiez M. A Comprehensive Overview of the Cyclodipeptide Synthase Family Enriched with the Characterization of 32 New Enzymes. Front. Microbiol. 2018, 9, 46 10.3389/fmicb.2018.00046. 29483897 PMC5816076

[ref41] Ogasawara Y.; Dairi T. Biosynthesis of Oligopeptides Using ATP-Grasp Enzymes. Chem.—Eur. J. 2017, 23, 10714–10724. 10.1002/chem.201700674. 28488371

[ref42] aFischbach M. A.; Clardy J. One pathway, many products. Nature Chem. Biol. 2007, 3, 353–355. 10.1038/nchembio0707-353. 17576415

[ref43] aThompson I. P.; Lilley A. K.; Ellis R. J.; Bramwell P. A.; Bailey M. J. Survival, Colonization and Dispersal of Genetically Modified *Pseudomonas fluorescens* SBW25 in the Phytosphere of Field Grown Sugar Beet. Biotechnol. 1995, 13, 1493–1497. 10.1038/nbt1295-1493.

[ref44] De Souza J. T.; de Boer M.; de Waard P.; van Beek T. A.; Raaijmakers J. M. Biochemical, genetic, and zoosporicidal properties of cyclic lipopeptide surfactants produced by *Pseudomonas fluorescens*. Appl. Environ. Microbiol. 2003, 69, 7161–7172. 10.1128/AEM.69.12.7161-7172.2003. 14660362 PMC309978

[ref45] Kolmogorov M.; Yuan J.; Lin Y.; Pevzner P. A. Assembly of long, error-prone reads using repeat graphs. Nat. Biotechnol. 2019, 37, 540–546. 10.1038/s41587-019-0072-8. 30936562

[ref46] aBreidenbach J.; Lemke C.; Pillaiyar T.; Schäkel L.; Al Hamwi G.; Diett M.; Gedschold R.; Geiger N.; Lopez V.; Mirza S.; Namasivayam V.; Schiedel A. C.; Sylvester K.; Thimm D.; Vielmuth C.; Vu L. P.; Zyulina M.; Bodem J.; Gütschow M.; Müller C. E. Targeting the Main Protease of SARS-CoV-2: From the Establishment of High Throughput Screening to the Design of Tailored Inhibitors. Angew. Chem., Int. Ed. 2021, 60, 10423–10429. 10.1002/anie.202016961. PMC8014119 33655614

[ref47] Xie X.; Muruato A.; Lokugamage K. G.; Narayanan K.; Zhang X.; Zou J.; Liu J.; Schindewolf C.; Bopp N. E.; Aguilar P. V.; Plante K. S.; Weaver S. C.; Makino S.; LeDuc J. W.; Menachery V. D.; Shi P. Y. An Infectious cDNA Clone of SARS-CoV-2. Cell Host Microbe 2020, 27, 841–848. 10.1016/j.chom.2020.04.004. 32289263 PMC7153529

[ref48] Arendrup M. C.; Meletiadis J.; Mouton J. W.; Lagrou K.; Hamal P.; Guinea J.; The Subcommittee on Antifungal Susceptibility Testing (AFST) of the ESCMID European Committee for Antimicrobial Susceptibility Testing (EUCAST). Method for the determination of broth dilution minimum inhibitory concentrations of antifungal agents for yeasts. EUCAST DEFINITIVE DOCUMENT E.DEF 7.3.2. April 2020. Accessed under https://www.eucast.org/astoffungi/methodsinantifungalsusceptibilitytesting/susceptibility_testing_of_yeasts/.

[ref49] Patel J. B.; Cockerill F. R.; Bradford P. A.; Eliopoulos G. M.; Hindler J. A.; Jenkins S. G.; Lewis J. S.; Limbago B.; Miller L. A.; Nicolau D. P.; Powell M.; Swenson J. M.; Turnidge J. D.; Weinstein M. P.; Zimmer B. L. Methods for Dilution Antimicrobial Susceptibility Tests for Bacteria that Grow Aerobically. Approved Standard, Vol. 35, 10th ed., Clinical and Laboratory Standards Institute, USA, 2015.

