# Supplementary material for: Salamandamide Lipodipeptides Are Biosynthetic Intermediate Shunt Products of the Nonamodular Nonribosomal Peptide Assembly Lines of the Viscosin Family
Source: J Nat Prod. 2025 Apr 15;88(4):1012–22. doi: 10.1021/acs.jnatprod.5c00084 (PMC12038845; doi:10.1021/acs.jnatprod.5c00084)
Supplement: Supplementary file 2 — np5c00084_si_002.pdf [file np5c00084_si_002.pdf]

■ SUPPORTING INFORMATION

Salamandamide lipodipeptides are biosynthetic intermediate shunt products of the nonamodular nonribosomal peptide assembly lines of the viscosin family

Keshab Bhattarai,[†] Thomas Majer,[†] Manuela Haussmann,[†] Dieter Schollmeyer,[‡] Markus Kramer,[§] Feyisara Eyiwumi Oni,[⊥] Monica Höfte,[ǀ] Rabea Voget,[▽] Michael Gütschow,[▽] Natalia Ruetalo,[○] Michael Schindler,[○] Jan Straetener,[±] Tatjana Wannenwetsch,[±] Heike Brötz-Oesterhelt,[±,^,#] Ryan Karongo,[∞] Benedikt Masberg,[∞] Michael Lämmerhofer,[∞] Rosanna Catherine Hennessy,[&] Carly R. Muletz-Wolz,[%] and Harald Gross[†,^,#,*]

[†]Pharmaceutical Institute, Department of Pharmaceutical Biology, University of Tübingen, 72076 Tübingen, Germany

[‡]Department of Chemistry, Johannes Gutenberg University Mainz, 55099 Mainz, Germany

[§]Institute of Organic Chemistry, University of Tübingen, 72076 Tübingen, Germany

[⊥]Department of Phytopathology, Rijk Zwaan Breeding B.V., 2678 ZG De Lier, The Netherlands

[ǀ]Lab. Phytopathology, Department of Plants and Crops, Faculty of Bioscience Engineering, Ghent University, 9000 Gent, Belgium

[▽]Pharmaceutical Institute, Pharmaceutical & Medicinal Chemistry, University of Bonn, 53121 Bonn, Germany

[○]Institute for Medical Virology and Epidemiology, Section Molecular Virology, University Hospital Tübingen, 72076 Tübingen, Germany

[±]Department of Microbial Bioactive Compounds, Interfaculty Institute of Microbiology and Infection Medicine, Tübingen (IMIT), University of Tübingen, 72076 Tübingen, Germany

[^]German Center for Infection Research (DZIF)

[#]Cluster of Excellence: EXC 2124: Controlling Microbes to Fight Infection, University of Tübingen, Tübingen, Germany

[∞]Pharmaceutical Institute, Department of Pharmaceutical Analysis and Bioanalysis, University of Tübingen, 72076 Tübingen, Germany

[&]Department of Plant and Environmental Sciences, University of Copenhagen, Frederiksberg, Denmark

[%]Center for Conservation Genomics, Smithsonian's National Zoo and Conservation Biology Institute, 20008 Washington, DC, United States of America

* E-mail: harald.gross@uni-tuebingen.de

Table of Contents

MS Molecular Networking Analysis of the Extract of *P. tolaasii* RSB 5.11

Figure S1 Complete MS molecular network.. S-4
Figure S2 MS molecular networking analysis focused on tolaasins................................ S-5

Spectral Data for Salamandamide A (2)

Figure S3 HR-ESI-MS spectrum.. S-6
Figure S4 HR-ESI-TOF-MS/MS spectrum.. S-6
Figure S5 UV spectrum.. S-7
Figure S6 FT-IR spectrum.. S-7
Figure S7 400 MHz ^{1}H NMR spectrum in d_6-DMSO.. S-8
Figure S8 100 MHz ^{13}C NMR spectrum in d_6-DMSO.. S-8
Figure S9 400 MHz DEPT135 NMR spectrum in d_6-DMSO................................ S-9
Figure S10 400 MHz multiplicity edited ^{1}H-^{13}C-HSQC NMR spectrum in d_6-DMSO..... S-9
Figure S11 400 MHz ^{1}H-^{1}H-COSY NMR spectrum in d_6-DMSO............................ S-10
Figure S12 100 MHz ^{1}H-^{1}H-NOESY NMR spectrum in d_6-DMSO......................... S-10
Figure S13 400 MHz ^{1}H-^{13}C-HSQC-TOCSY spectrum in d_6-DMSO........................ S-11
Figure S14 400 MHz ^{1}H-^{13}C-HMBC NMR spectrum in d_6-DMSO.......................... S-12
Figure S15 400 MHz band-selective ^{1}H-^{13}C-HMBC NMR spectrum S-12
Figure S16 400 MHz ^{1}H-^{15}N-HSQC NMR spectrum in d_6-DMSO........................... S-13
Figure S17 400 MHz ^{1}H-^{15}N-HMBC NMR spectrum in d_6-DMSO.......................... S-13
Figure S18 Chiral LC-MS analysis of the lipid- and the peptide-portion of **2**................ S-14

Spectral Data for Pseudodesmin A (3)

Figure S19 HR-ESI-MS spectrum.. S-15
Figure S20 HR-ESI-TOF-MS/MS spectrum... S-15
Figure S21 FT-IR spectrum.. S-16
Table S1 NMR spectroscopic data of **3** in comparison with published data................. S-17
Figure S22 400 MHz ^{1}H NMR spectrum in d_3-CH_3CN... S-18
Figure S23 100 MHz ^{13}C NMR spectrum in d_3-CH_3CN.. S-18
Figure S24 400 MHz ^{1}H-^{1}H-TOCSY NMR spectrum in d_3-CH_3CN........................ S-19
Figure S25 400 MHz ^{1}H-^{13}C-HSQC-TOCSY spectrum in d_3-CH_3CN....................... S-20
Figure S26 Chiral LC-MS analysis of the lipid-portion of **3**... S-20
Figure S27 Picture of crystalline pseudodesmin A (**3**).. S-21
Figure S28 Molecular crystal packing of **3**.. S-21
Figure S29 The asymmetric unit of **3**.. S-22
Table S2 Crystal data and structure refinement for **3**.. S-23
Table S3 Atomic coordinates and equiv. isotropic displacement parameters for **3**......... S-24
Table S4 Anisotropic displacement parameters for **3**.. S-26

Spectral Data for Pseudodesmin B (4)

Figure S30 HR-ESI-MS spectrum.. S-27
Table S5 NMR spectroscopic data of **4** in comparison with published data.................. S-28
Figure S31 400 MHz ^{1}H NMR spectrum in d_3-CH_3CN... S-29
Figure S32 100 MHz ^{13}C NMR spectrum in d_3-CH_3CN.. S-29
Figure S33 400 MHz multiplicity edited ^{1}H-^{13}C-HSQC NMR spectrum in d_3-CH_3CN..... S-30

Spectral Data for Salamandamide B (5)

Figure S34 HR-ESI-MS spectrum....S-31
Figure S35 HR-ESI-TOF-MS/MS spectrum....S-31
Figure S36 700 MHz ^{1}H NMR spectrum in d_6-DMSO....S-32
Figure S37 175 MHz ^{13}C NMR spectrum in d_6-DMSO....S-32
Figure S38 700 MHz multiplicity edited ^{1}H-^{13}C-HSQC NMR spectrum in d_6-DMSO....S-33
Figure S39 700 MHz ^{1}H-^{1}H-COSY NMR spectrum in d_6-DMSO....S-33
Figure S40 700 MHz ^{1}H-^{13}C-HSQC-TOCSY spectrum in d_6-DMSO....S-34
Figure S41 700 MHz selective TOCSY spectra in d_6-DMSO....S-35
Figure S42 700 MHz ^{1}H-^{13}C-HMBC NMR spectrum in d_6-DMSO....S-36
Figure S43 700 MHz ^{1}H-^{1}H-NOESY NMR spectrum in d_6-DMSO....S-36
Figure S44 700 MHz ^{1}H-^{15}N-HSQC NMR spectrum in d_6-DMSO....S-37

Biosynthetic Data

Figure S45 Comparative LC/MS analysis of the *visc* mutants....S-38

Biological Assays

Table S6 Results of the protease inhibition assays....S-39
Figure S46 Effect of **2** and **3** on SARS-CoV-2 infection....S-40
Figure S47 Pre-treatment of Caco-2 cells with **2** and **3** reduces viral infection....S-41
Table S7 Results of the antimicrobial and cytotoxicity assays....S-42

Figure S1. Complete MS molecular networking analysis of the extract of *P. tolaasii* RSB 5.11. Each node represents a molecular ion, connected by edges. The size of the node represents intensity the of respective feature. The thickness of the edge corelates the cosine score (similarity extent) between the connected nodes. The red border shows network created by tolaasin derivatives, while the blue circle the one generated by pseudodesmins and salamandamide A. The blue rectangular insert shows the latter in an enlarged form.

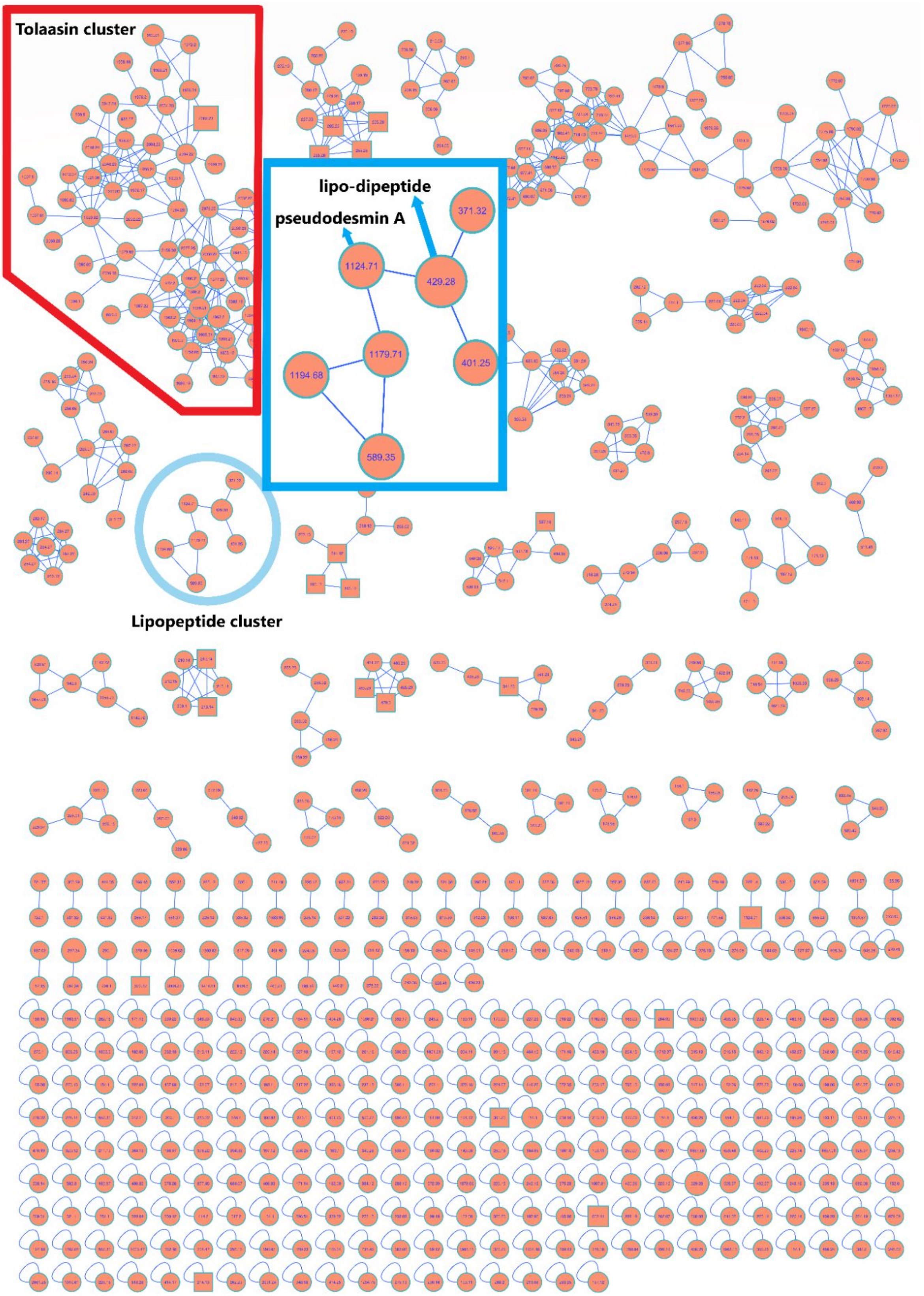

Figure S2. MS molecular networking analysis focused on the tolaasin MS-cluster.

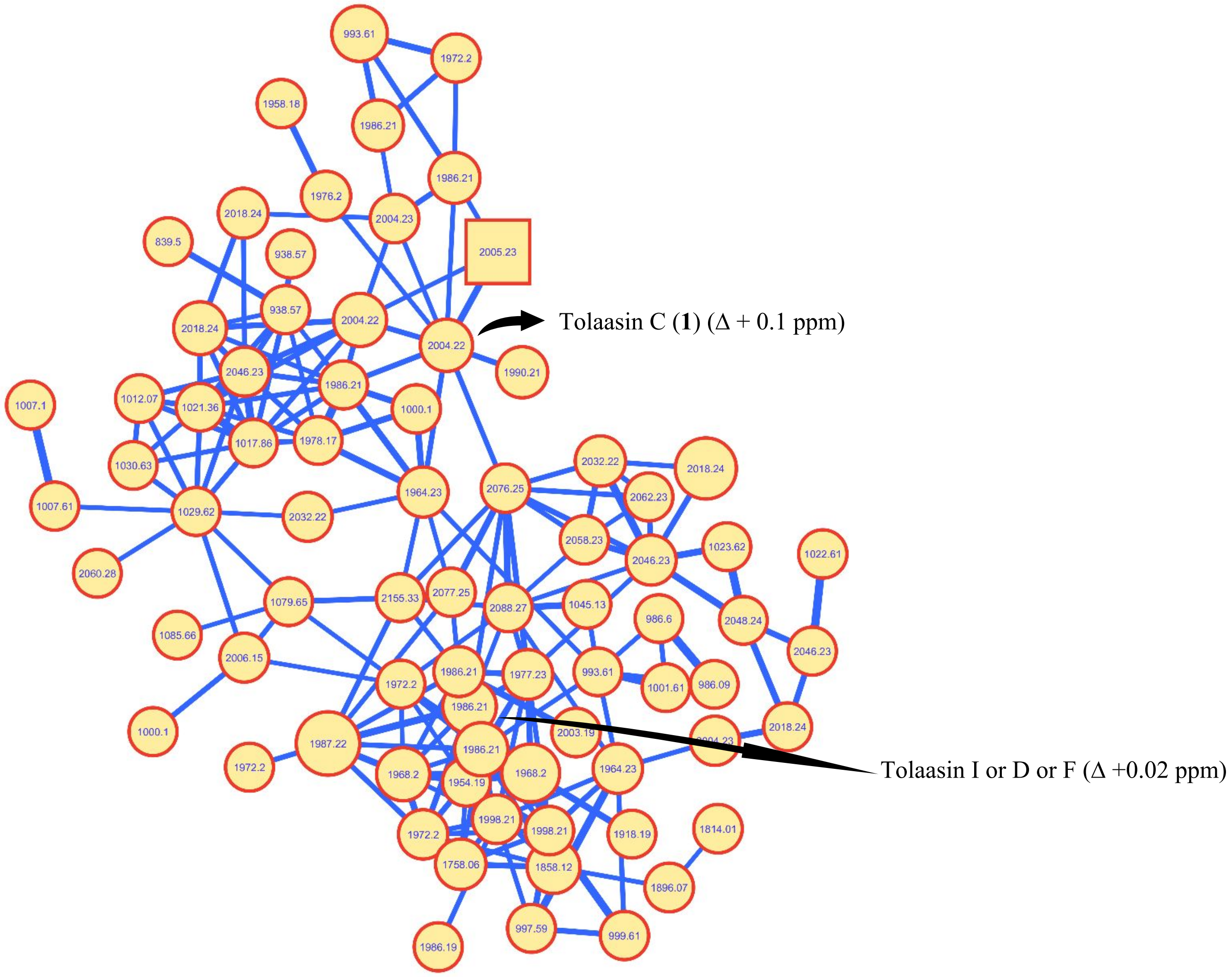

Spectral Data for Salamandamide A (2)

Figure S3. HR-ESI-MS spectrum of **2** (positive mode).

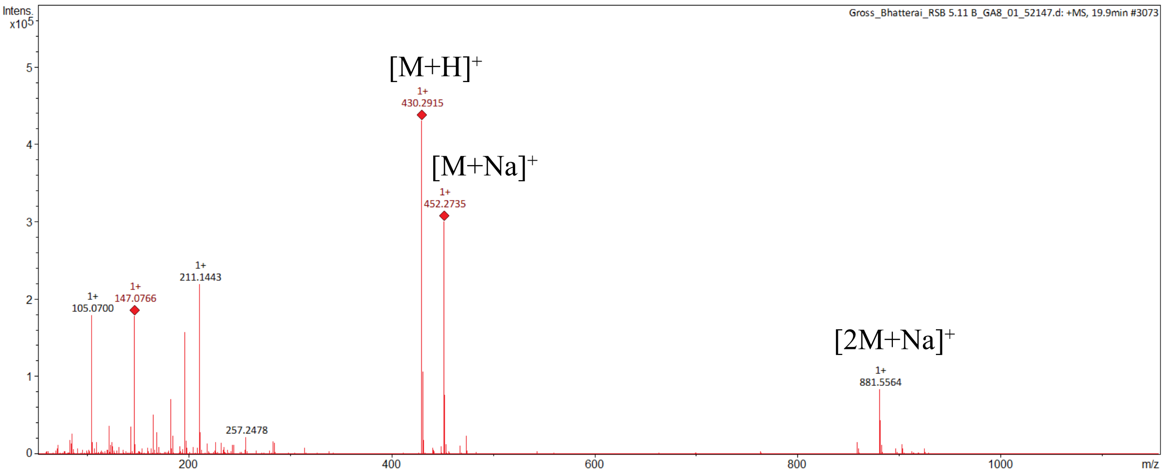

Figure S4. HR-ESI-TOF-MS/MS spectrum of **2**.

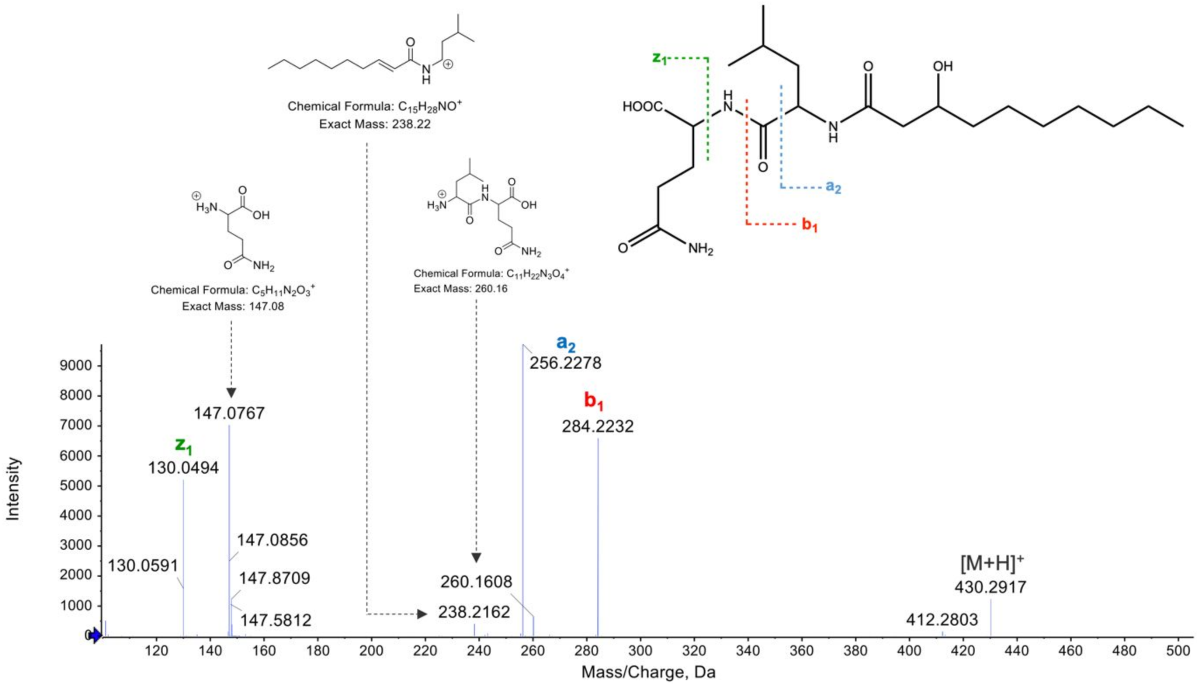

Figure S5. UV spectrum of **2** in MeOH.

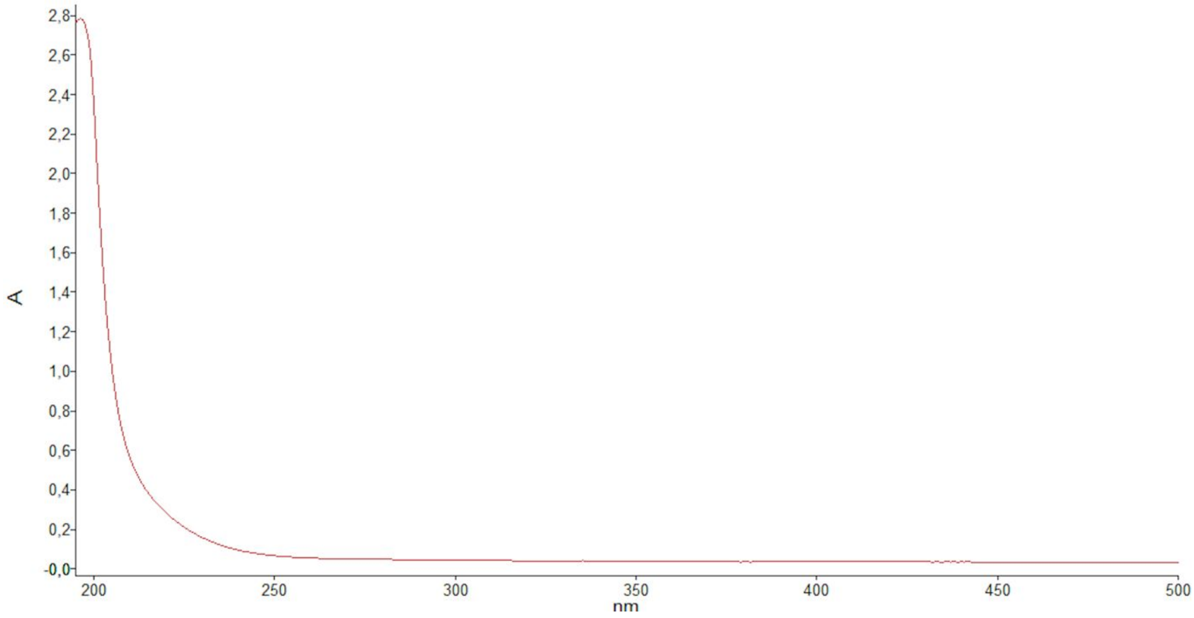

Figure S6. FT-IR spectrum of **2**.

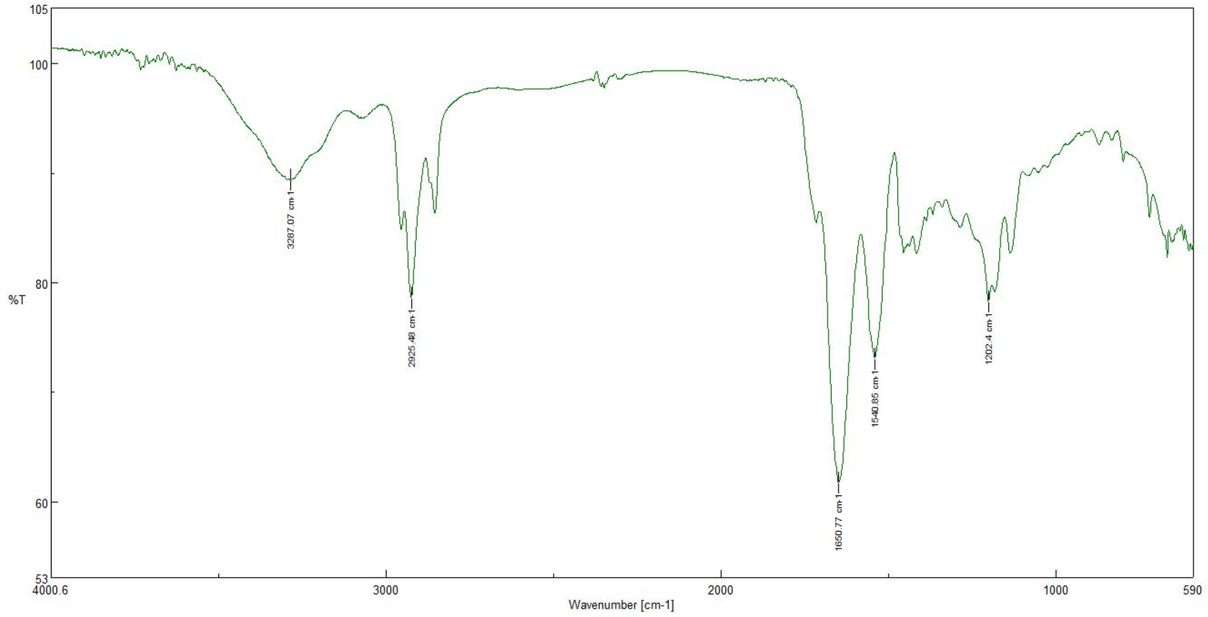

Figure S7. 400 MHz ^{1}H NMR spectrum of **2** in d_6-DMSO.

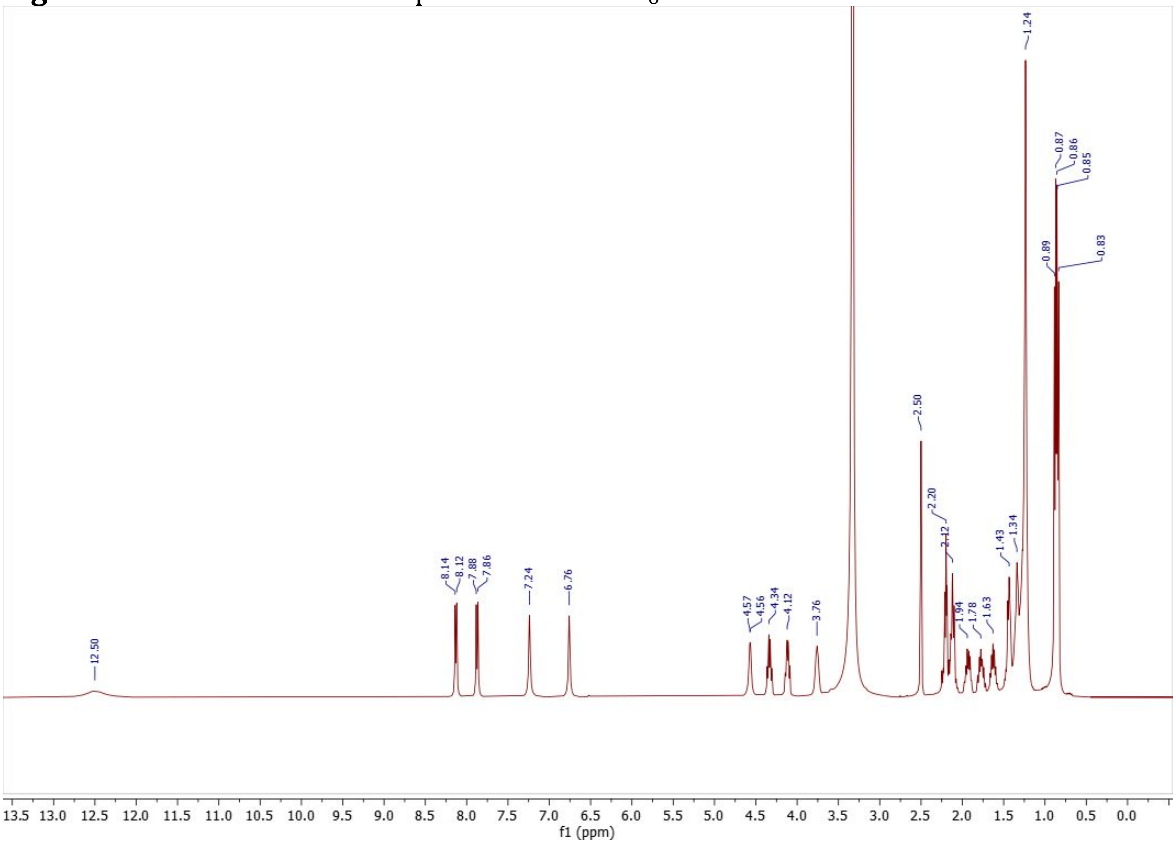

Figure S8. 100 MHz ^{13}C NMR spectrum of **2** in d_6-DMSO.

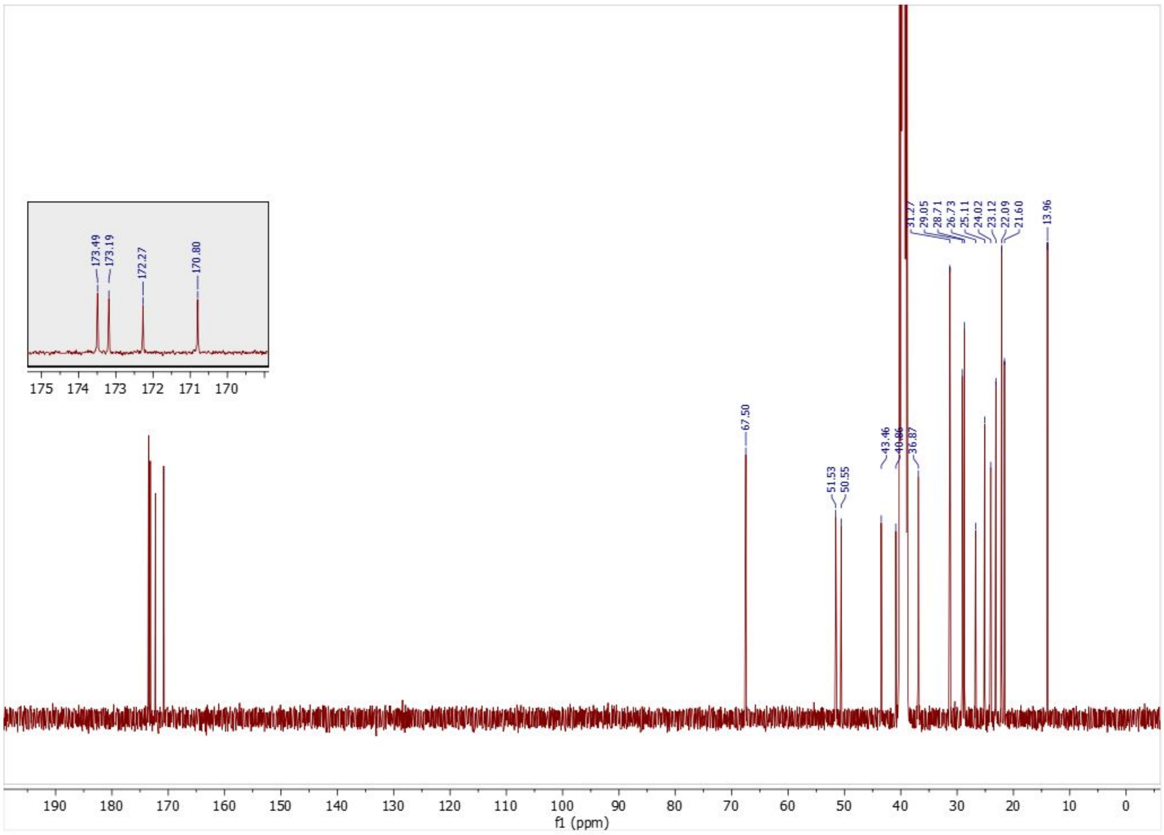

Figure S9. 400 MHz DEPT135 NMR spectrum of **2** in d_6-DMSO, superimposed with a 100 MHz ^{13}C NMR spectrum of **2**.

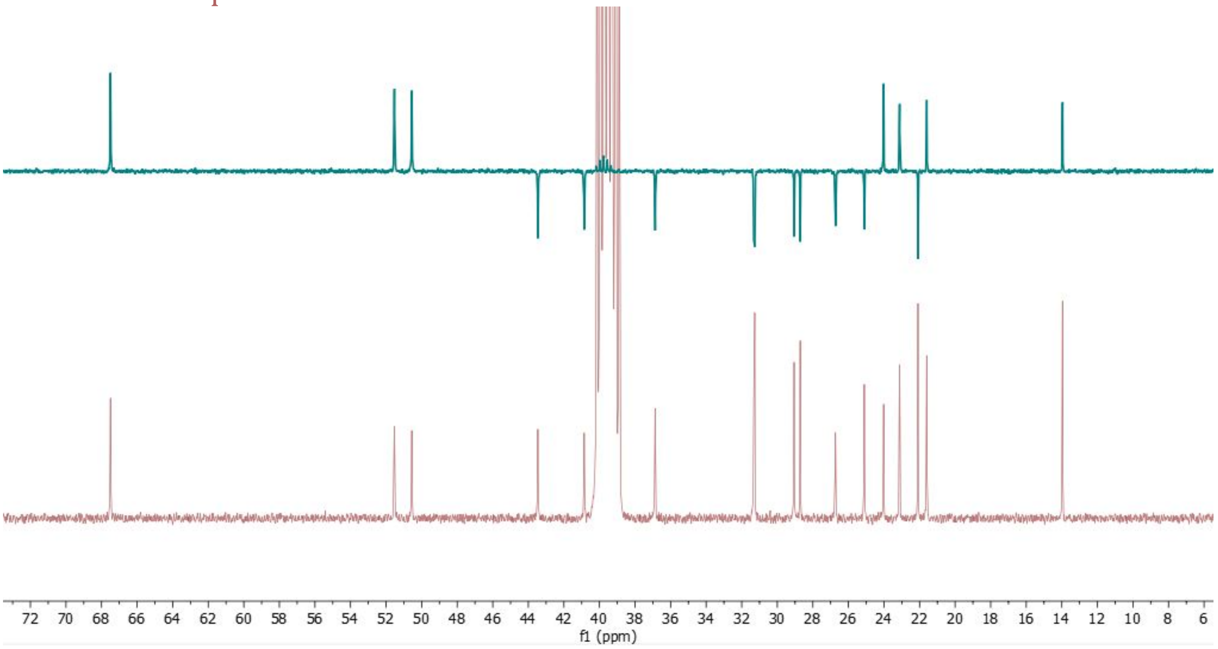

Figure S10. 400 MHz multiplicity edited ^{1}H-^{13}C-HSQC NMR spectrum of **2** in d_6-DMSO. CH_2-groups are indicated by blue crosspeaks, while CH and CH_3 groups occur as red crosspeaks.

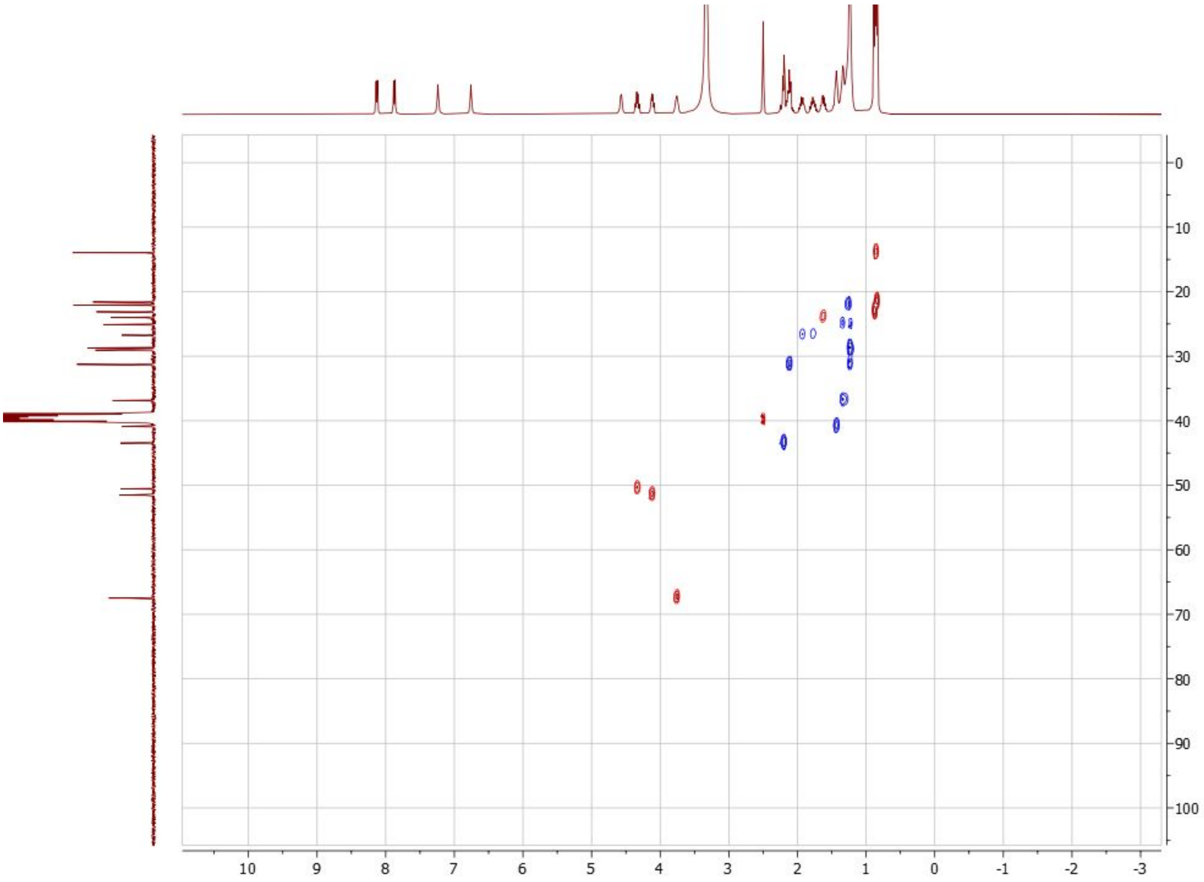

Figure S11. 400 MHz ^{1}H-^{1}H-COSY (cosygpppqf) NMR spectrum of **2** in d_6-DMSO. Bold lines in the insert visualize the observed correlations.

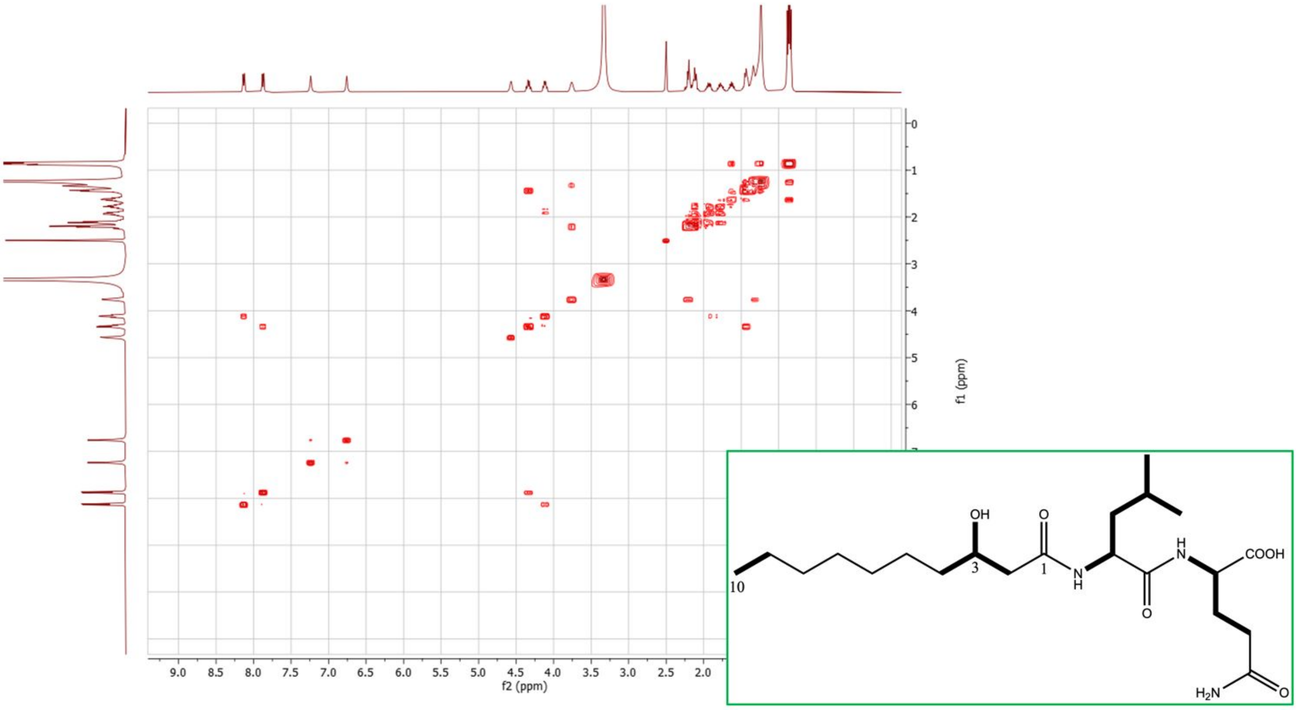

Figure S12. 400 MHz ^{1}H-^{1}H-NOESY NMR spectrum of **2** in d_6-DMSO.

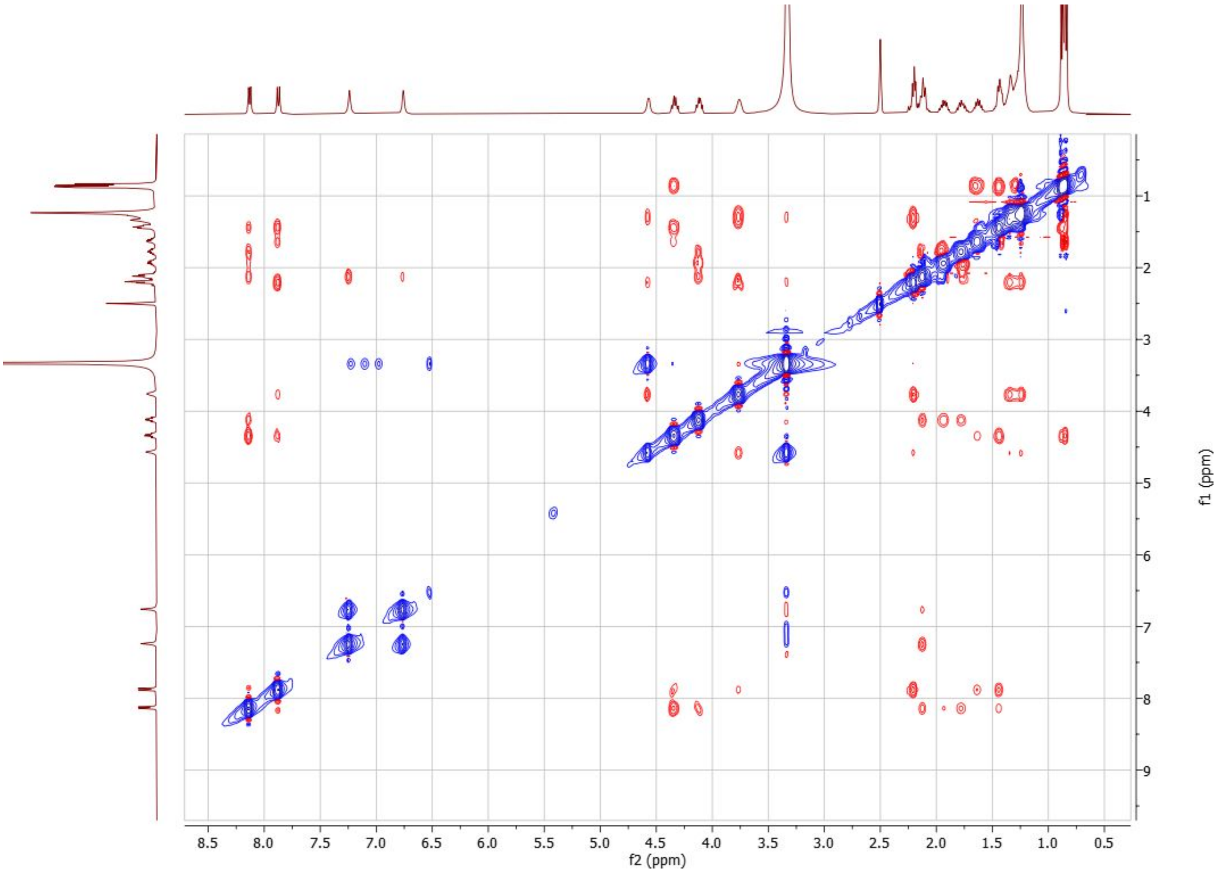

Figure S13. 400 MHz ^{1}H-^{13}C-HSQC-TOCSY spectrum of **2** in d_6-DMSO. Vertical lines are showing, color-code-assisted the three spin systems given in **2**.

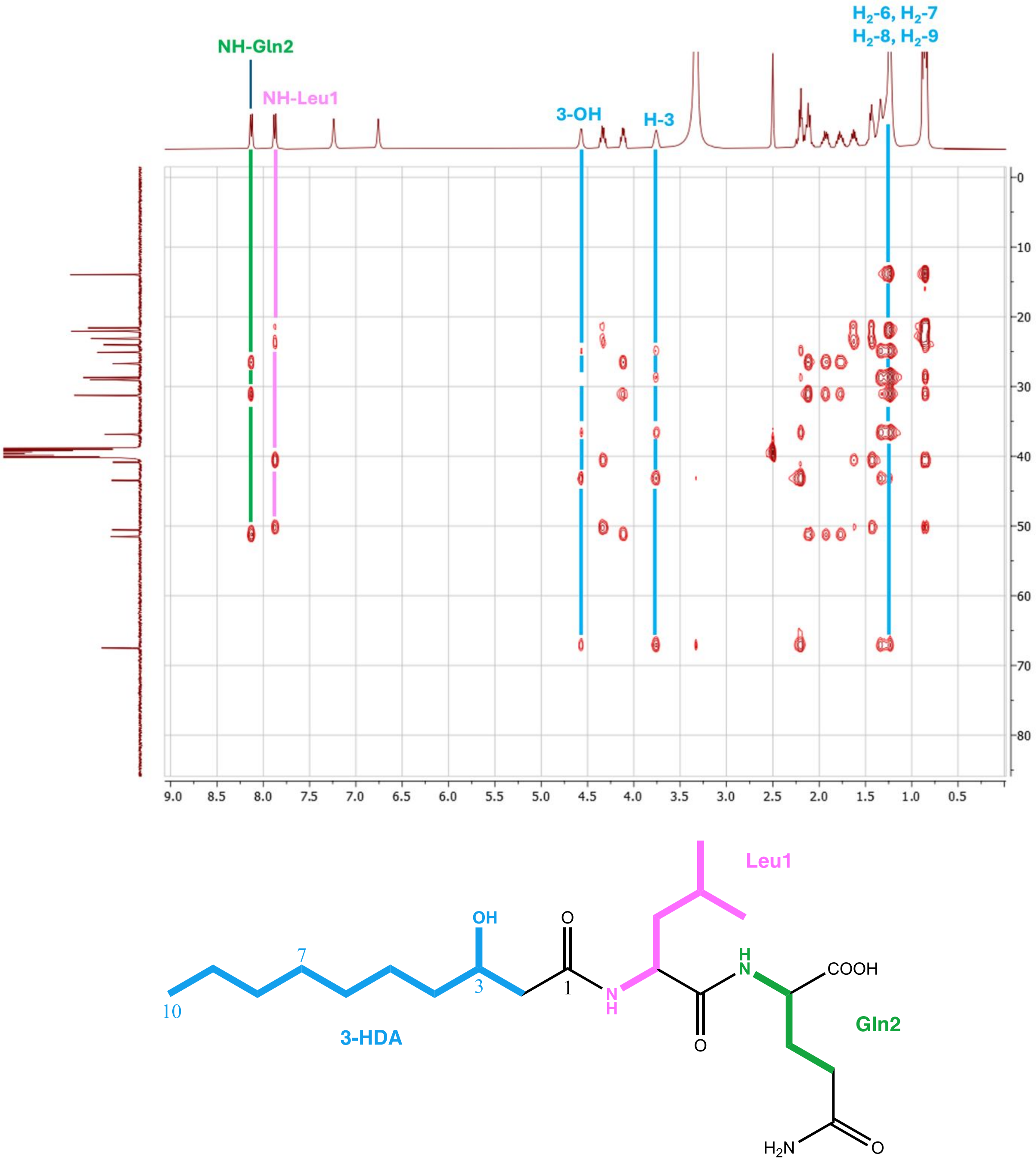

Figure S14. 400 MHz ^{1}H-^{13}C-HMBC NMR spectrum of **2** in d_6-DMSO.

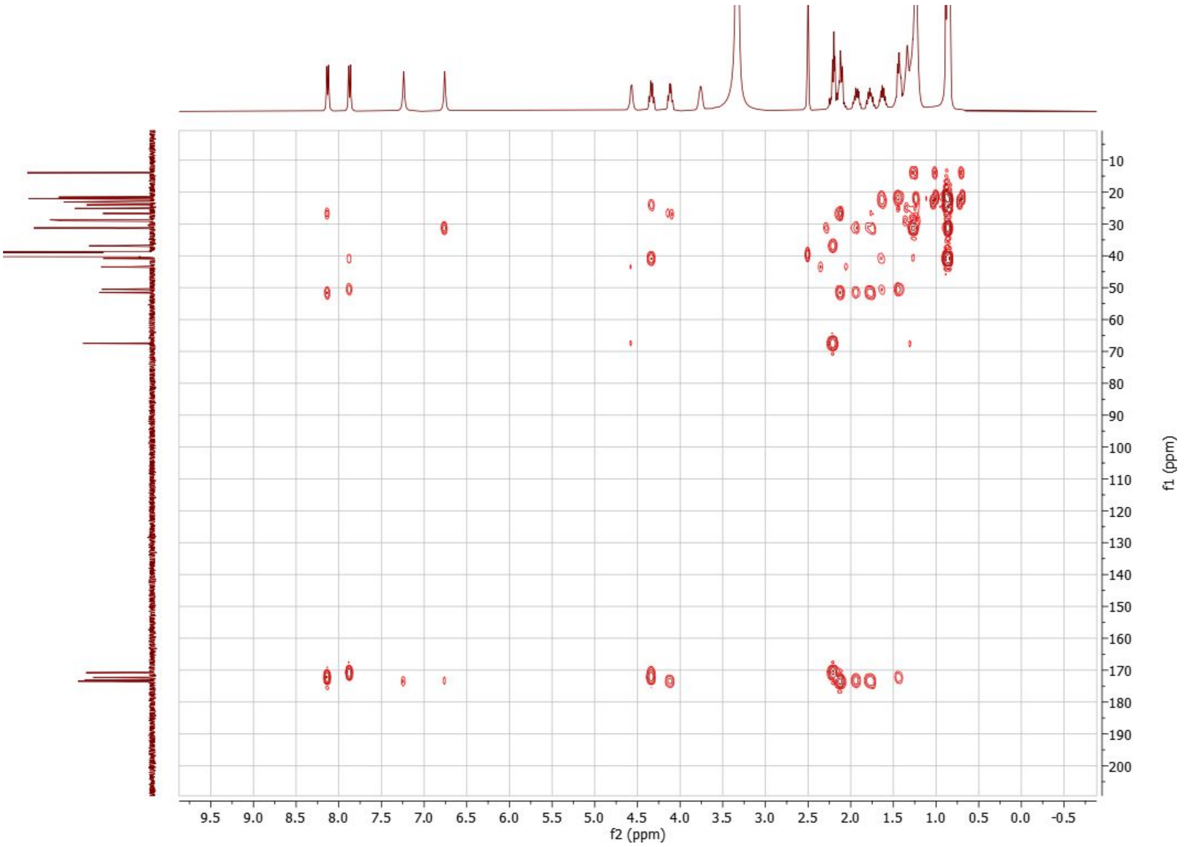

Figure S15. 400 MHz band-selective ^{1}H-^{13}C-HMBC NMR spectrum of **2** (δ_C 164.5-179.5).

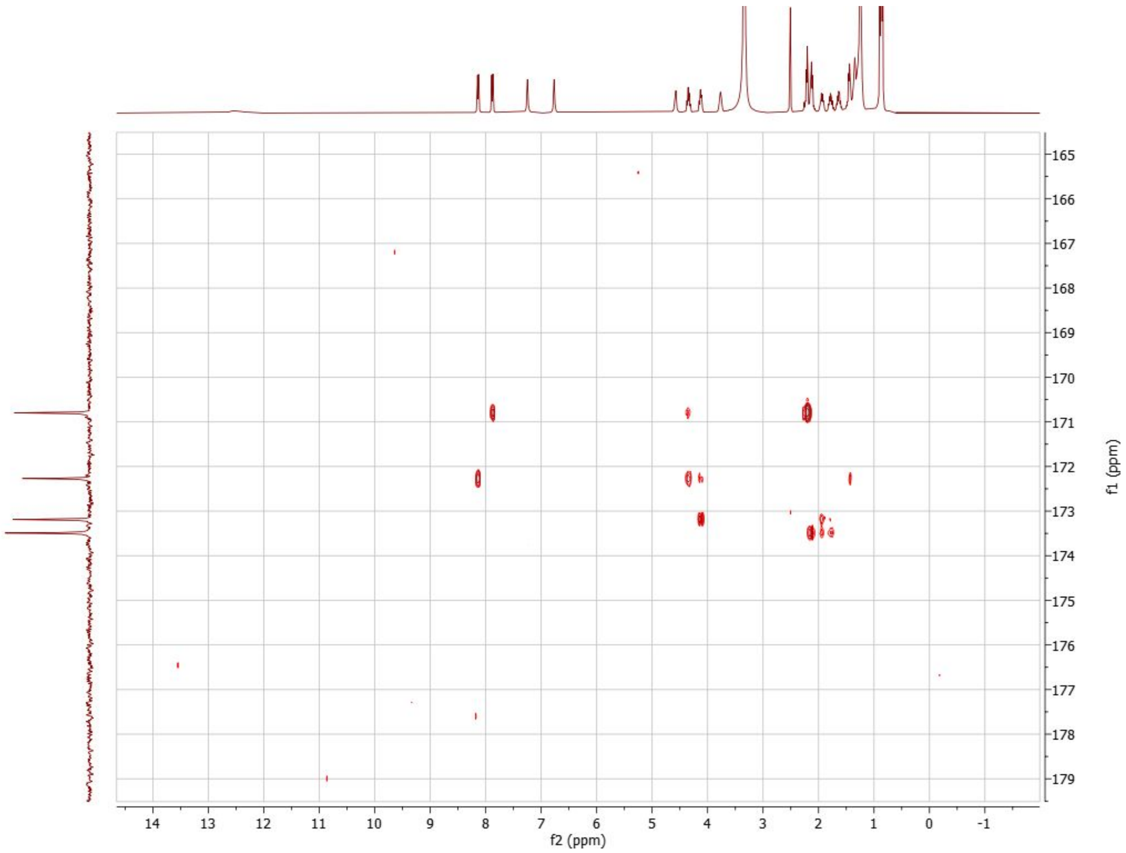

Figure S16. 400 MHz ^{1}H-^{15}N-HSQC NMR spectrum of **2** in d_6-DMSO.

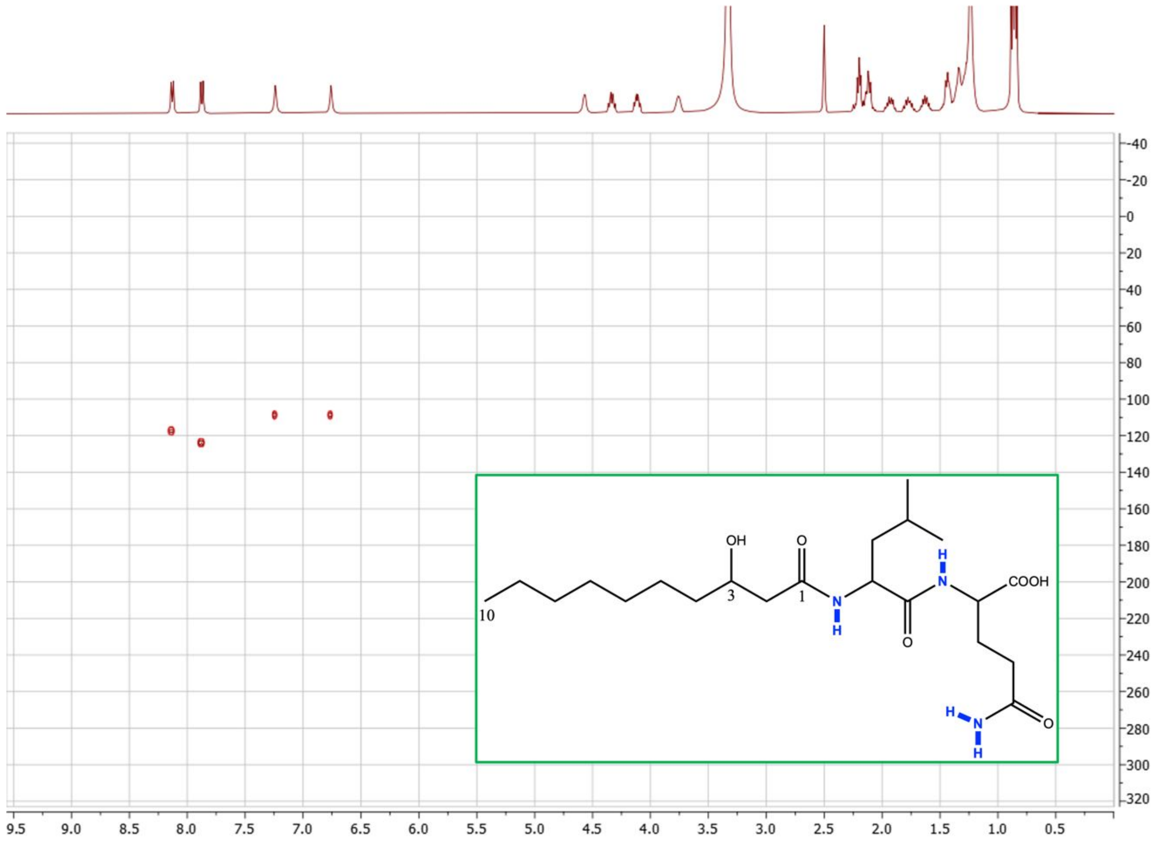

Figure S17. 400 MHz ^{1}H-^{15}N-HMBC NMR spectrum of **2** in d_6-DMSO.

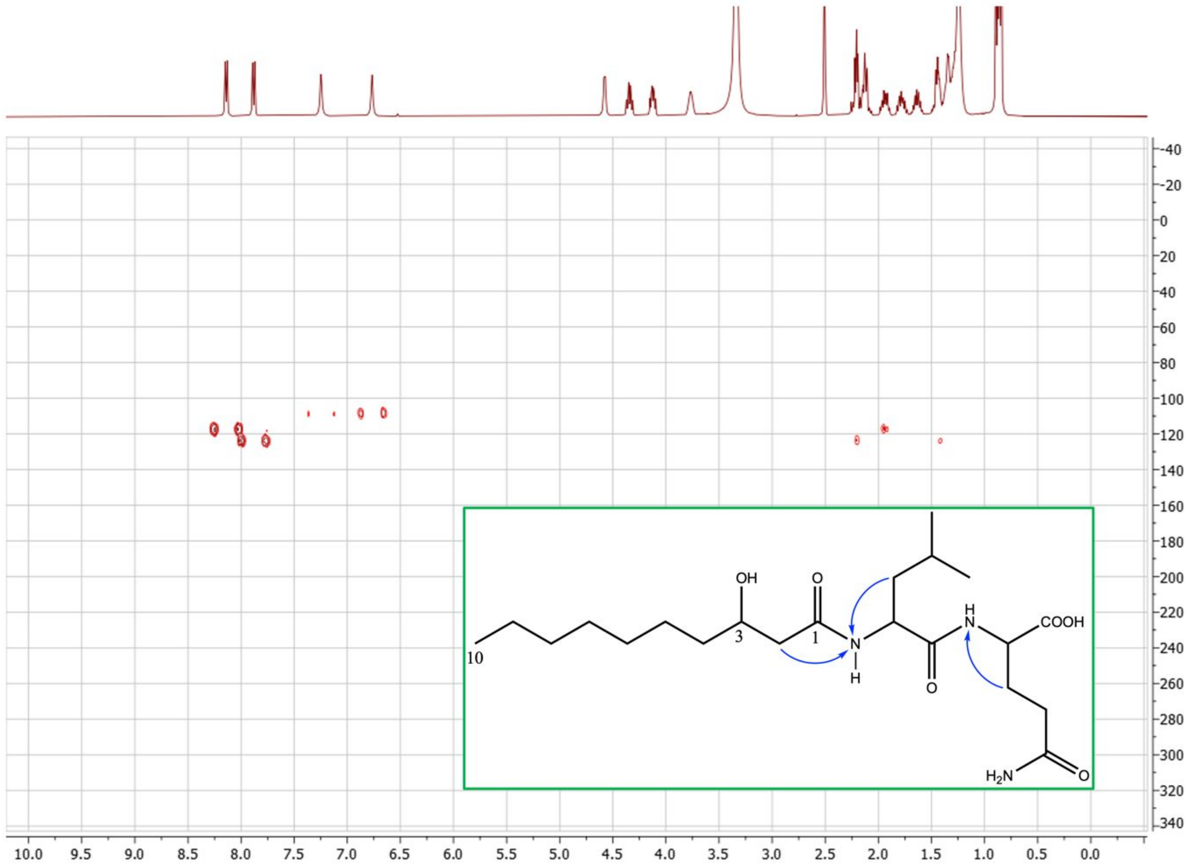

Figure S18. Chiral LC-MS analysis of the lipid- (**A**) and the peptide-(**B,C**) portion of **2.** Note regarding S18-B: D-Leu, D-Ile and the corresponding D-*allo* versions elute before 8 min. (**D**) 3-Hydroxydecanoic acid standards. Black, racemic standard, purchased from Sigma Aldrich (Merck). Red, 3-hydroxydecanoic acid with *R*-configuration from rhamnolipid R-95 (Sigma Aldrich) by alkaline hydrolysis.

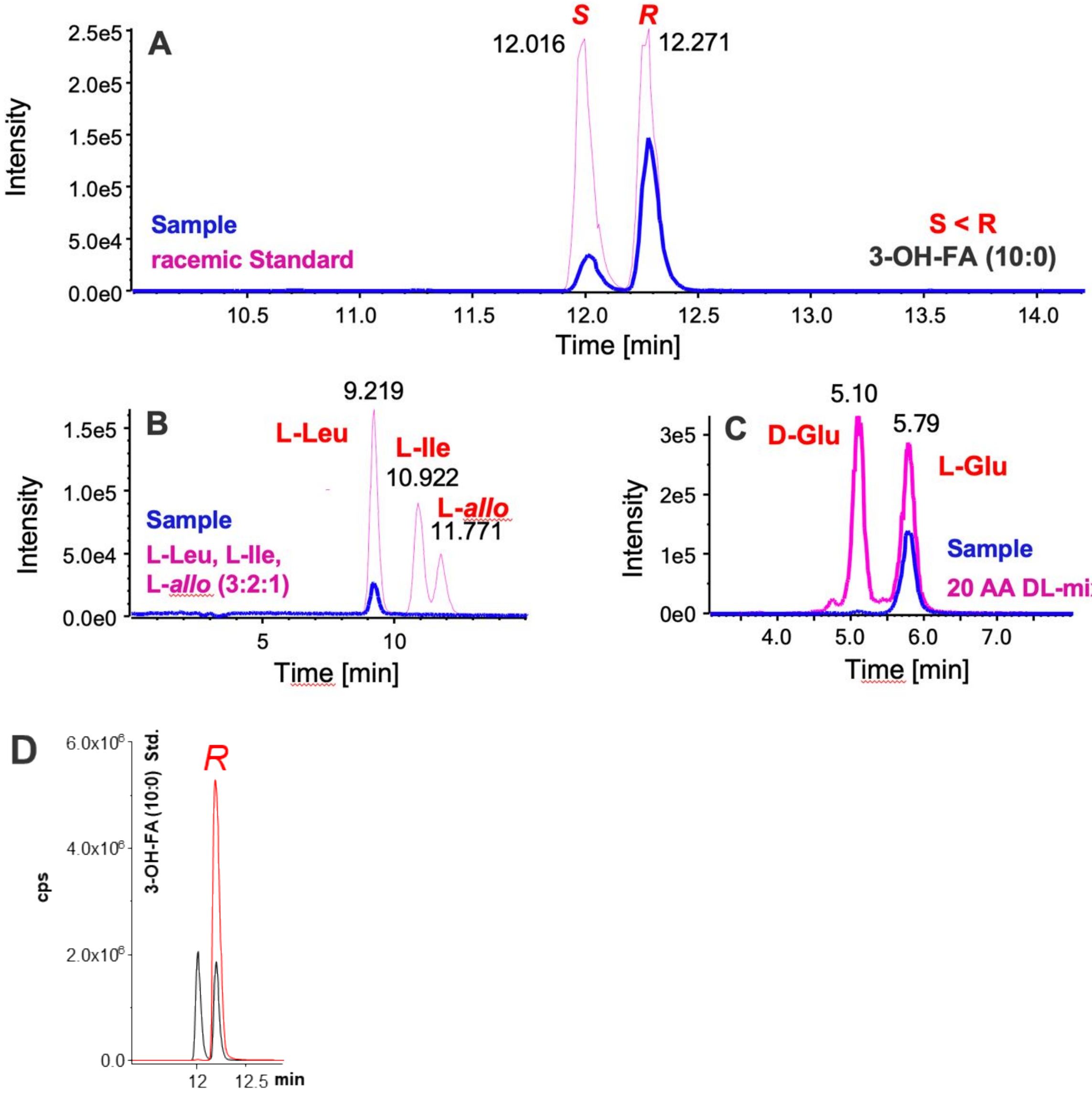

Spectral Data for Pseudodesmin A (3)

Figure S19. HR-ESI-MS spectrum of **3** (positive mode).

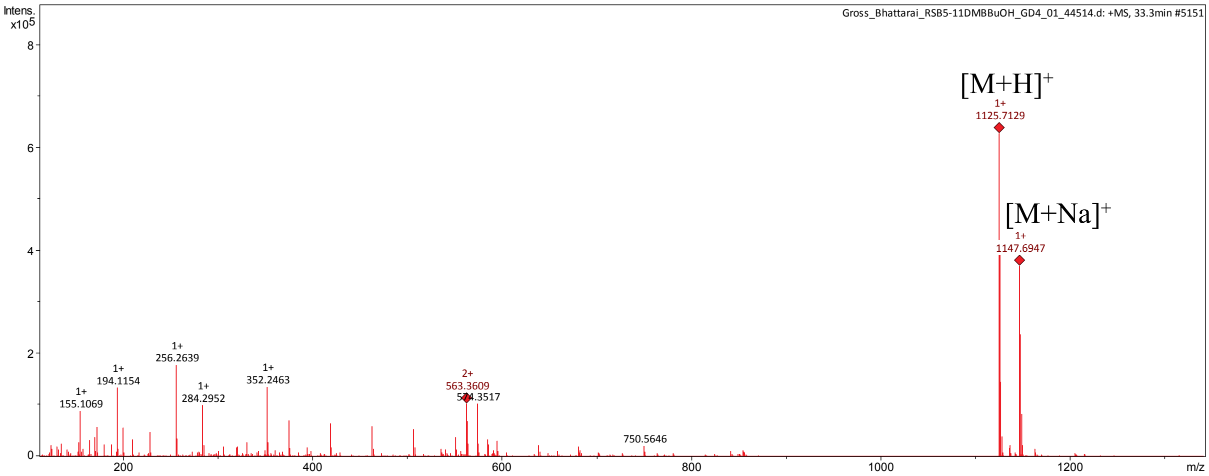

Figure S20. Top: HR-ESI-TOF-MS/MS spectrum of **3**. **Bottom:** Under MS/MS conditions, the ester-bond is cleaved and subsequent water elimination from threonine leads to the corresponding vinylic derivative. The structural diagram visualizes the b-ion series observed in the top MS/MS spectrum.

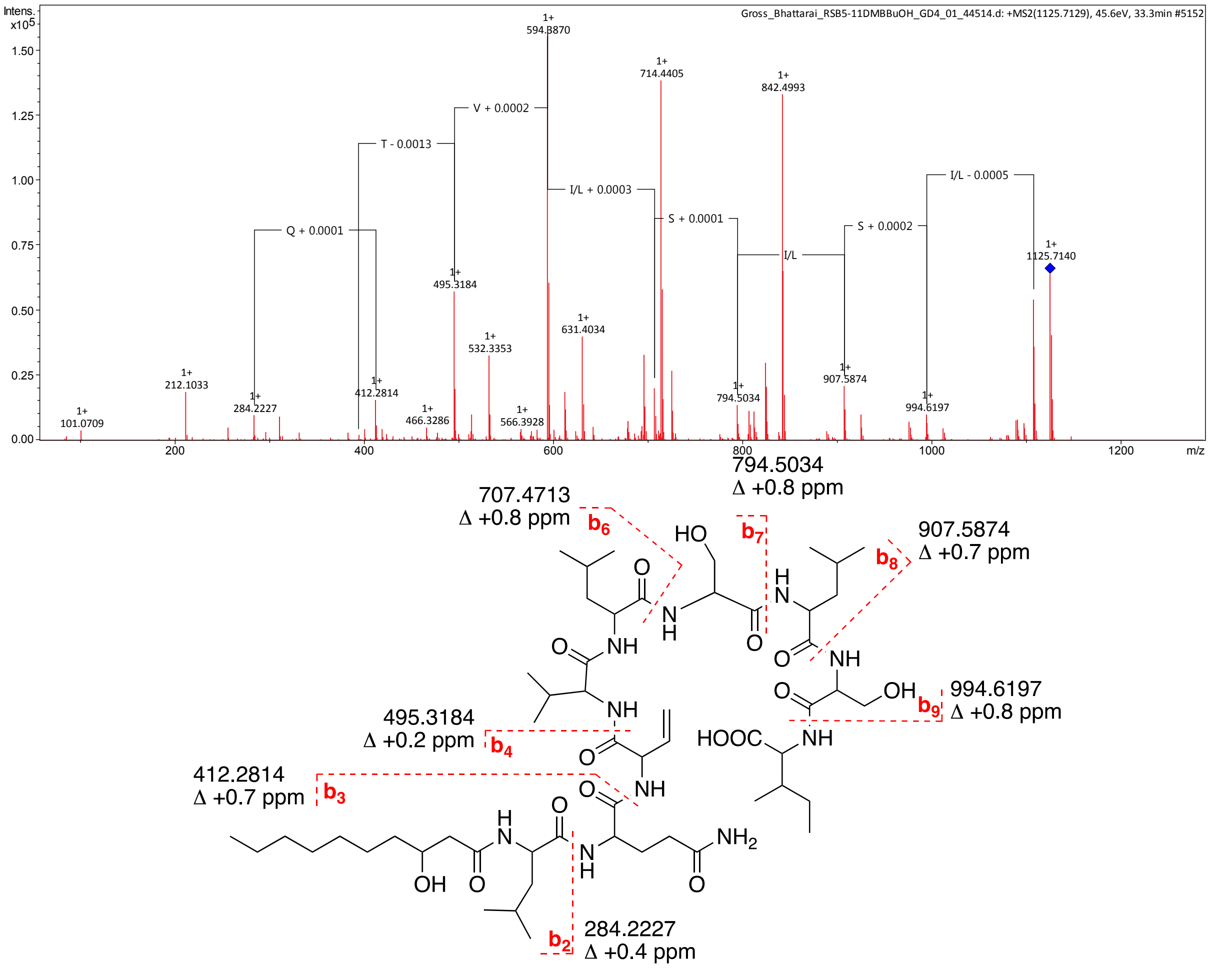

Figure S21. FT-IR spectrum of **3**.

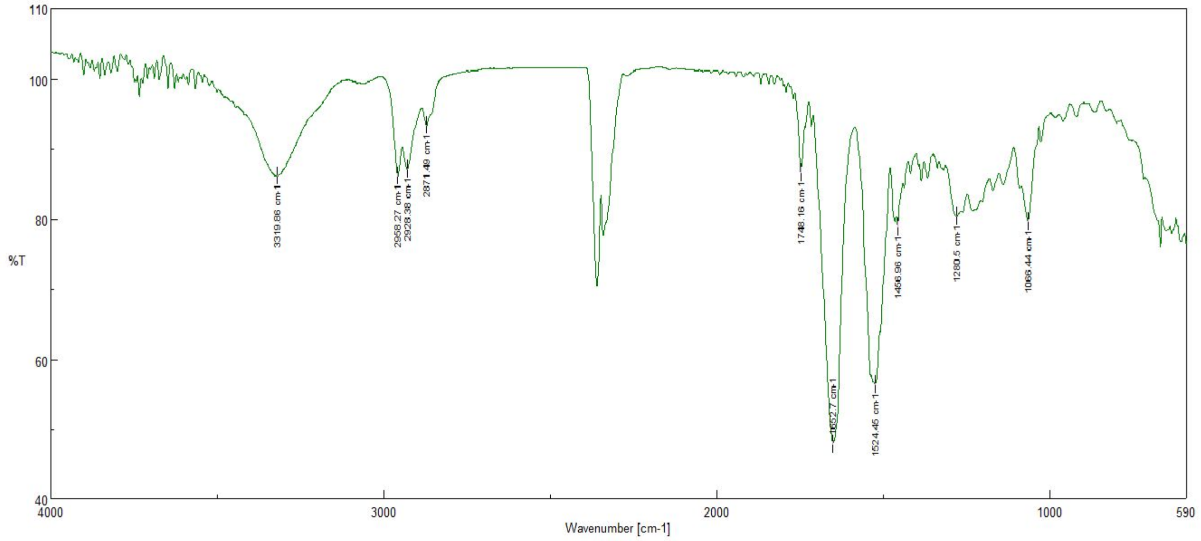
110
100
80
%T
60
40
4000
3000
2000
1000
590
Wavenumber [cm-1]
3319.86 cm-1
2958.27 cm-1
2928.38 cm-1
2871.49 cm-1
1748.16 cm-1
1652.7 cm-1
1524.45 cm-1
1456.96 cm-1
1280.5 cm-1
1066.44 cm-1

Table S1. ^{1}H and ^{13}C NMR assignments of pseudodesmin A (**3**) in d_3-CH_3CN in comparison with published values.

Residue	Atom	Pseudodesmin A (this study)[a]		Pseudodesmin A (lit. data)[b]		Δ(δ_H)	Δ(δ_C)
		δ_H [ppm]	δ_C [ppm]	δ_H [ppm]	δ_C [ppm]		
HDA	CO-1		175.21		175.27		-0.06
	CH_2-2	2.34/2.43	44.76	2.34/2.43	44.81	±0.00	-0.05
	CH-3	3.98	69.52	3.97	69.53	+0.01	-0.01
	CH_2-4	1.47	38.19	1.47	38.27	±0.00	-0.08
	CH_2-5	1.31/1.41	26.31	1.31/1.41	26.36	±0.00	-0.05
	CH_2-6	1.29	30.25	1.29	30.20	±0.00	+0.05
	CH_2-7	1.29	30.03	1.29	30.20	±0.00	-0.17
	CH_2-8	1.27	32.58	1.27	32.63	±0.00	-0.05
	CH_2-9	1.29	23.39	1.29	23.44	±0.00	-0.05
	CH_2-10	0.88	14.41	0.88	14.44	±0.00	-0.03
Leu1	NH	7.78 d 5.6		7.73 d 5.7		+0.05	
	CO		175.28		175.34		-0.06
	CH-α	3.87	53.89	3.87	53.92	±0.00	-0.03
	CH_2-β	1.65/1.74	39.36	1.65/1.74	39.46	±0.00	-0.10
	CH-γ	1.68	25.39	1.67	25.46	±0.00	-0.07
	CH_3-δ	0.90	22.14	0.90	22.16	±0.00	-0.02
	CH_3-ε	0.94	23.17	0.94	23.21	±0.00	-0.04
Gln2	NH	8.76 d 3.6		8.76 d 4.0		±0.00	
	CO		176.59		176.63		-0.04
	CH-α	3.99 m	57.52	3.98	57.58	+0.01	-0.06
	CH_2-β	2.03 m	26.28	2.02	26.35	+0.01	-0.07
	CH_2-γ	2.38 m	31.92	2.38	32.01	±0.00	-0.09
	CO-δ		176.18 w		176.15		+0.03
	NH_2	5.88 s / 6.38 s		5.81 s / 6.36 s		+0.07 / +0.02	
Thr3	NH	8.13 d 7.2		7.45 d 7.5		+0.70	
	CO		174.30		174.33		-0.03
	CH-α	4.00 m	61.71	3.99	61.77	+0.01	-0.06
	CH-β	5.32 dq	70.29	5.33	70.33	-0.01	-0.04
	CH_3-γ	1.30 m	18.53	1.30	18.58	±0.00	-0.05
Val4	NH	7.34 d 6.3		7.33 d 6.3		+0.01	
	CO		174.59		174.63		-0.04
	CH-α	3.49	65.02	3.49	65.08	±0.00	-0.06
	CH-β	2.18	29.96	2.17	30.02	+0.01	-0.06
	CH_3-γ	0.92	19.50	0.92	19.56	±0.00	-0.06
	CH_3-ε	0.95	21.06	0.95	21.12	±0.00	-0.06
Leu5	NH	7.73 d 3.8		7.72 d 4.3		+0.01	
	CO		173.58		173.62		-0.04
	CH-α	3.98 m	55.69	3.97	55.74	+0.01	-0.05
	CH_2-β	1.51/1.67	40.64	1.51/1.66	40.71	±0.00 / +0.01	-0.07
	CH-γ	1.77	25.48	1.77	25.53	±0.00	-0.05
	CH_3-δ	0.86	23.31	0.87	23.36	-0.01	-0.05
	CH_3-ε	0.86	21.29	0.88	21.34	-0.02	-0.05
Ser6	NH	7.13 d 8.6		7.11 d 8.5		+0.02	
	CO		172.00		171.93		+0.07
	CH-α	4.33	56.39	4.32	56.45	+0.01	-0.06
	CH_2-β	3.81/4.16	64.73	3.81/4.15	64.80	±0.00 / +0.01	-0.07
Leu7	NH	7.12 d 5.4		7.10 d 6.5		+0.02	
	CO		173.88		173.80		+0.08
	CH-α	4.14	54.90	4.13	54.90	+0.01	±0.00
	CH_2-β	1.58/1.88	42.04	1.58/1.88	42.11	±0.00 / ±0.00	-0.07
	CH-γ	1.89	25.48	1.89	25.55	±0.00	-0.07
	CH_3-δ	0.90	21.37	0.90	21.42	±0.00	-0.05
	CH_3-ε	0.99	23.47	0.99	23.53	±0.00	-0.06
Ser8	NH	7.96 d 8.8		7.94 d 9.1		+0.02	
	CO		171.83		171.86		-0.03
	CH-α	4.45	56.98	4.43	57.02	+0.02	-0.04
	CH_2-β	3.67/3.86	63.18	3.66/3.86	63.25	+0.01 / ±0.00	-0.07
Ile9	NH	6.69 d 10.1		6.68 d 10.7		+0.01	
	CO		170.04		170.12		-0.08
	CH-α	4.56	57.14	4.56	57.17	±0.00	-0.03
	CH-β	1.97	36.88	1.97	36.97	±0.00	-0.09
	CH_2-γ	n.o./1.15	25.24	0.97/1.15	25.31	n.o. / ±0.00	-0.07
	CH_3-δ	0.86	12.30	0.86	12.36	±0.00	-0.06
	CH_3-ε	0.82	16.23	0.82	16.28	±0.00	-0.05

[a]400/100 MHz, 298 K, referenced to the residual solvent signal of CD_3CN at δ_H/δ_C = 1.94/1.32; [b]700/175 MHz, 298 K, referenced to TMS, values taken from Tetrahedron 65 (2009) 4173–4181; w = weak resonance; n.o. = not observed

Figure S22. 400 MHz ^{1}H NMR spectrum of **3** in d_3-CH_3CN.

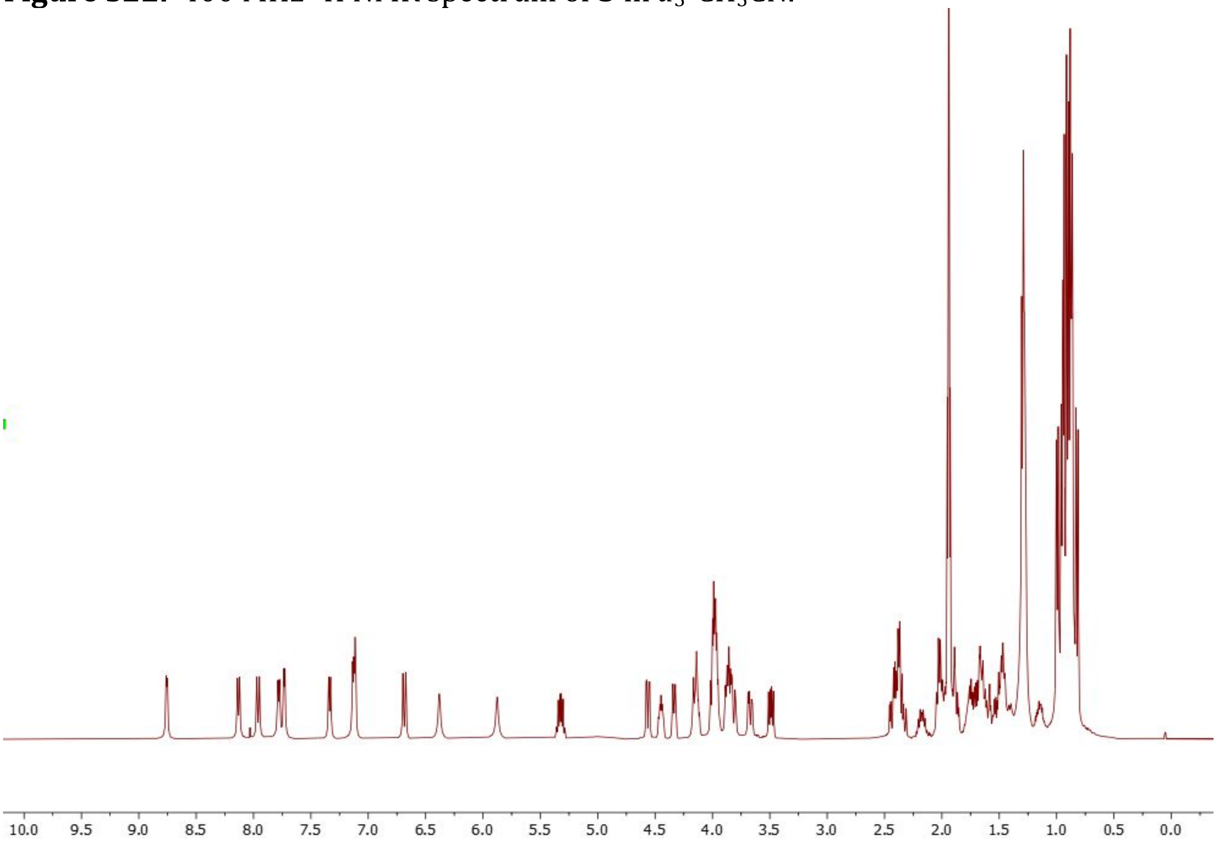

Figure S23. 100 MHz ^{13}C NMR spectrum of **3** in d_3-CH_3CN.

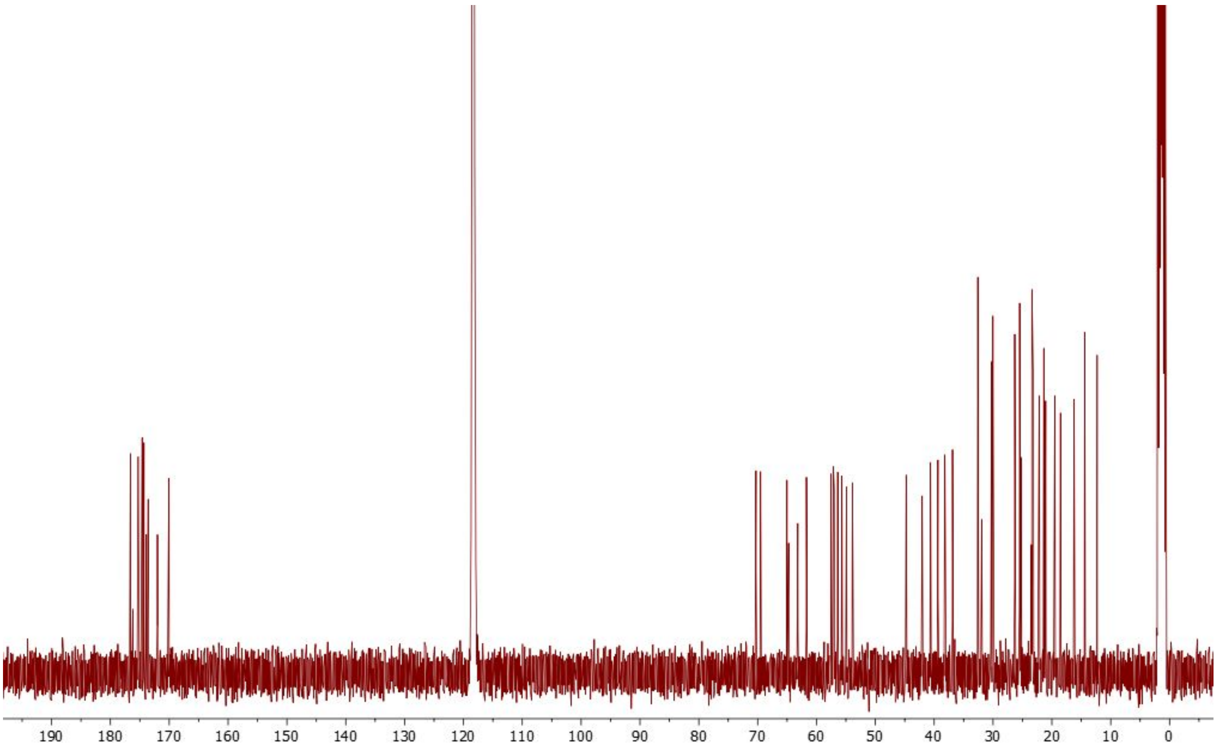

Figure S24. 400 MHz ^1H-^1H-TOCSY NMR spectrum of **3** in d_3-CH_3CN. Vertical lines in the insert are showing, color-code-assisted the amino acid-based spin systems given in **3**.

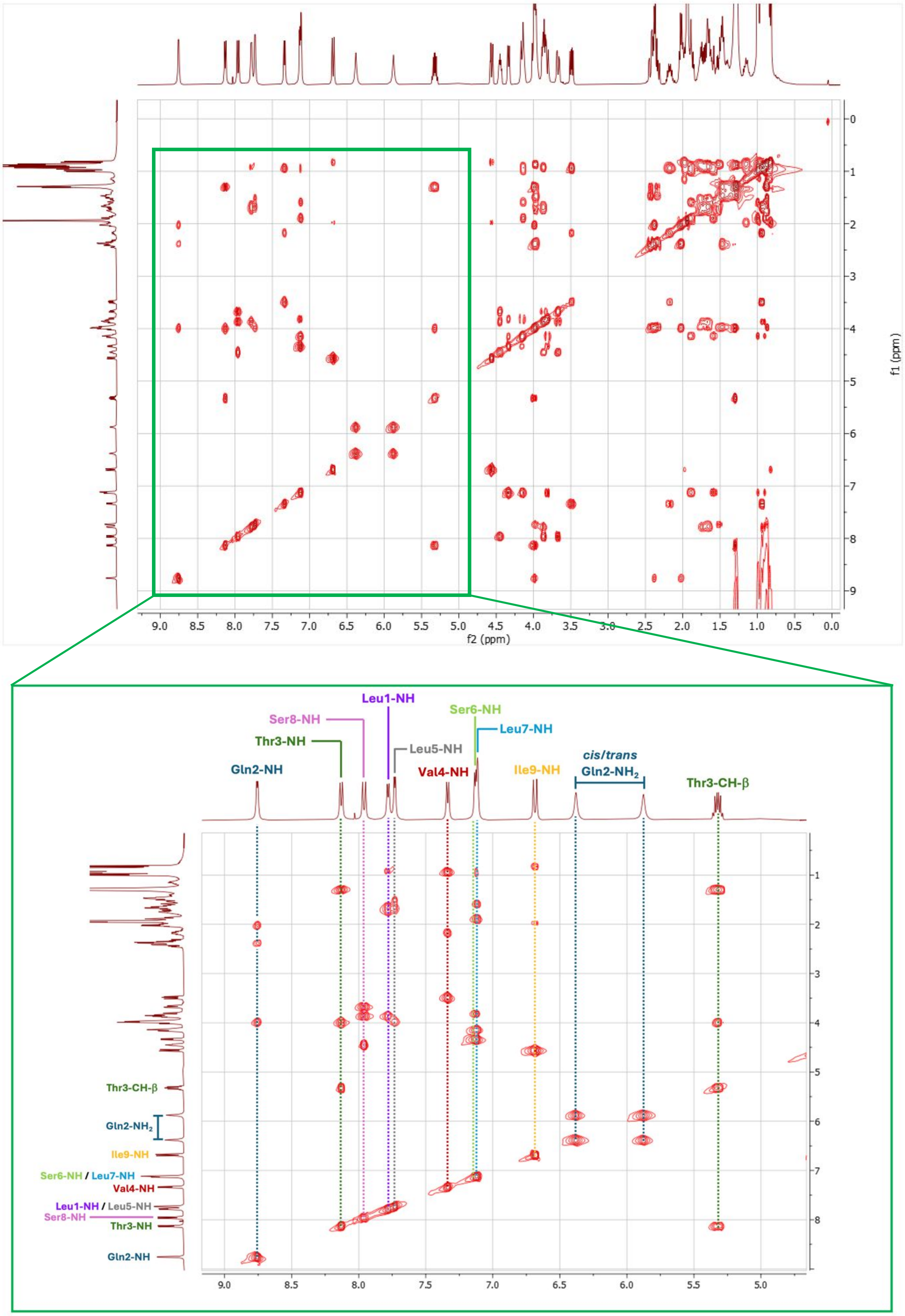

Figure S25. 400 MHz ^{1}H-^{13}C-HSQC-TOCSY spectrum of **3** in d_3-CH_3CN.

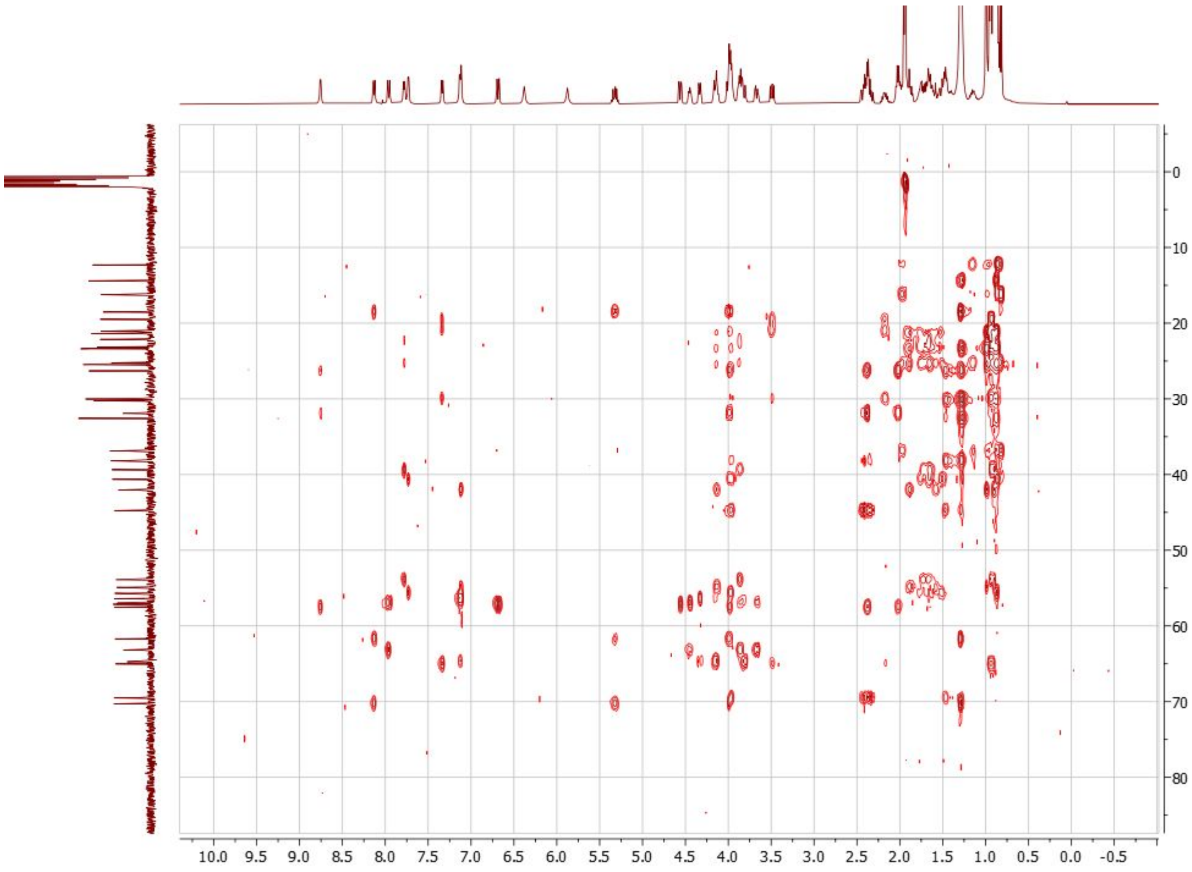

Figure S26. Chiral LC-MS analysis of the lipid-portion of **3.** Note, the shift toward shorter retentions times of *S*- and *R*-3-OH-decanoic acid compared to Figure S18 A and D is explainable by a higher column temperature, that was applied during this analysis (as outlined in the experimental section)

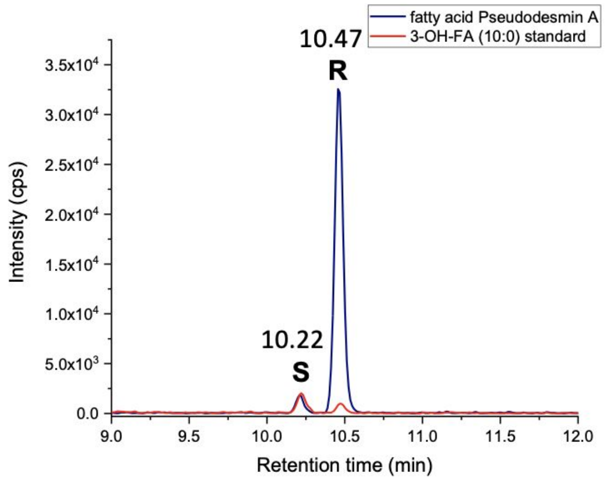

Fatty acid	Config.	Retention time t_R / min	Peak area
**3-OH-capric acid (10:0)**	(S)	10.22	6982
	(R)	10.47	129360
			Ratio (S:R) 1:18.5

Figure S27. Picture of crystalline pseudodesmin A (**3**)

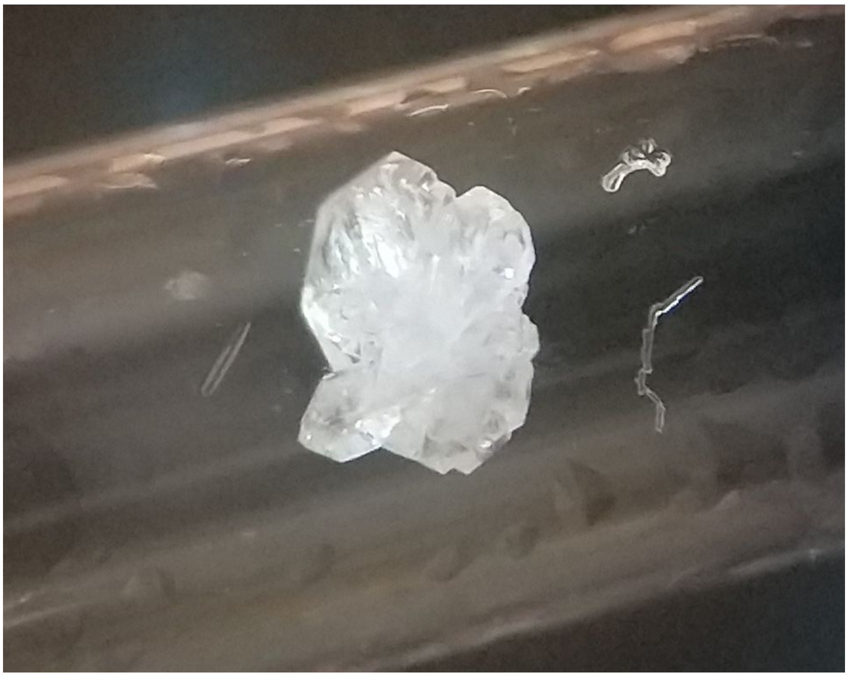

Figure S28. Molecular crystal packing of **3**.

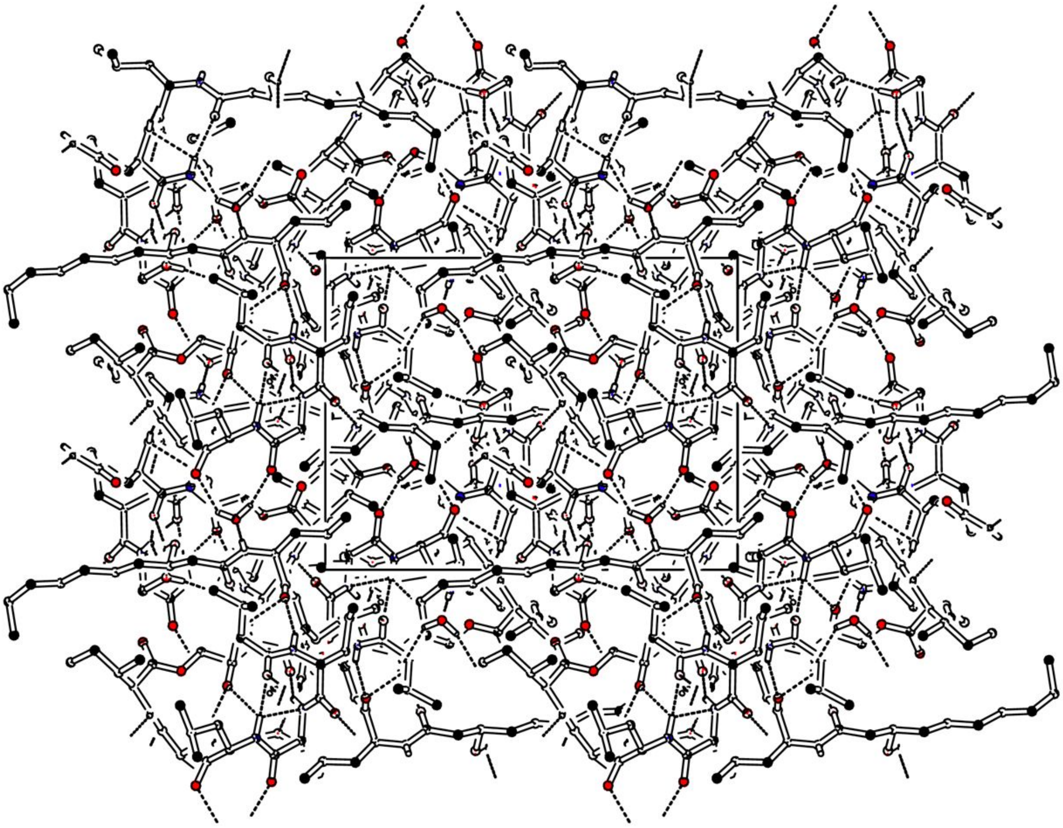

Figure S29. The asymmetric unit of **3**.

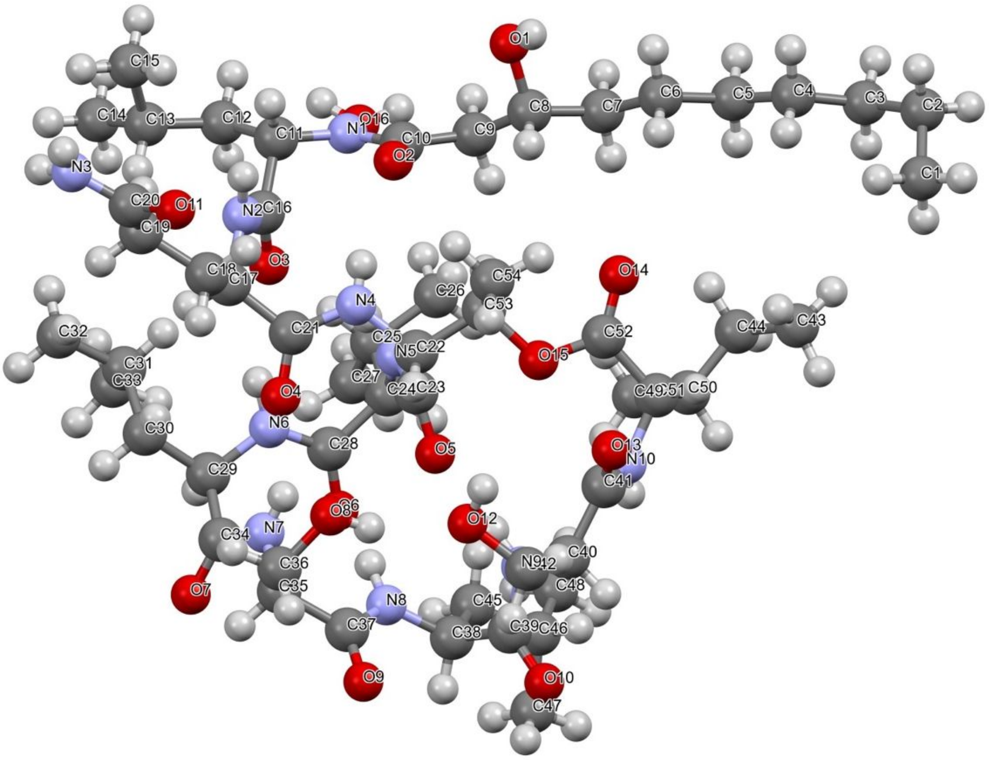

Table S2. Crystal data and structure refinement for pseudodesmin A (**3**).

Identification code	CCDC 2380284
Empirical formula	$C_{54}H_{98}N_{10}O_{16}$
moiety formula	$C_{54}H_{96}N_{10}$ O_{15}, H_2O
Formula weight	1143.42
Temperature	120(2) K
Wavelength, radiation type	1.54178Å, CuKα
Crystal system	Orthorhombic
Space group name, number	P $2_12_12_1$, (19)
Unit cell dimensions	a = 14.2065(3) Å
	b = 18.7763(6) Å
	c = 24.5388(5) Å
Volume	6545.6(3) $Å^3$
Number of reflections	77641
and range used for lattice parameters	2.96° <=θ<= 69.77°
Z	4
Density (calculated)	1.160 Mg/m^3
Absorption coefficient	0.704 mm^{-1}
Absorption correction	None
F(000)	2480
Crystal size, colour and form	0.140 x 0.250 x 0.320 mm^3, colorless block
Theta range for data collection	2.963 to 69.497°.
Index ranges	-17<=h<=16, -22<=k<=19, -29<=l<=29
Number of reflections:	
collected	63509
independent	12065 [R(int) = 0.0326]
observed [I>2sigma(I)]	10160
Completeness to theta = 67.7°	99.8 %
Refinement method	Full-matrix least-squares on F^2
Data / restraints / parameters	12065 / 28 / 769
Goodness-of-fit on F^2	1.024
Final R indices [I>2sigma(I)]	R1 = 0.0511, wR2 = 0.1420
R indices (all data)	R1 = 0.0594, wR2 = 0.1538
Flack parameter	0.05(7)
Largest diff. peak and hole	0.568 and -0.444 $eÅ^{-3}$

Table S3. Atomic coordinates and equivalent isotropic displacement parameters (Å^2) for pseudodesmin A (**3**). U(eq) is defined as one third of the trace of the orthogonalized U_{ij} tensor.

Atom	x	y	z	U(eq)
O1	0.41975(17)	0.63531(15)	0.51078(10)	0.0383(6)
O2	0.54476(17)	0.76809(14)	0.54136(10)	0.0375(6)
O3	0.58861(16)	0.90163(14)	0.63814(9)	0.0338(5)
O4	0.82629(17)	0.91475(15)	0.59694(10)	0.0375(6)
O5	0.87258(16)	0.76276(15)	0.67356(10)	0.0381(6)
O6	0.8373(2)	0.85938(17)	0.78409(11)	0.0495(7)
O7	0.96070(17)	1.02363(15)	0.75603(11)	0.0414(6)
O8	1.02452(18)	0.88533(16)	0.58650(10)	0.0432(6)
O9	1.17904(19)	0.86959(19)	0.69758(13)	0.0576(8)
O10	1.19204(18)	0.68613(17)	0.72383(12)	0.0489(7)
O12	1.0790(2)	0.75033(16)	0.56957(12)	0.0487(7)
O13	1.0089(2)	0.59509(18)	0.55643(10)	0.0486(7)
O14	0.7328(2)	0.55790(18)	0.57257(13)	0.0567(8)
O15	0.82434(17)	0.65039(14)	0.59490(10)	0.0354(5)
N1	0.4384(2)	0.80483(17)	0.60294(13)	0.0353(7)
N2	0.59112(19)	0.92769(16)	0.54830(11)	0.0310(6)
N4	0.7332(2)	0.82728(17)	0.56477(11)	0.0312(6)
N5	0.71695(19)	0.78587(17)	0.67500(11)	0.0316(6)
N6	0.7584(2)	0.92099(17)	0.71986(12)	0.0365(7)
N7	0.9458(2)	0.94582(19)	0.68613(12)	0.0363(7)
N8	1.0389(2)	0.83138(19)	0.72962(13)	0.0401(7)
N9	1.0576(2)	0.69433(19)	0.67513(13)	0.0388(7)
N10	0.9553(2)	0.57542(17)	0.64114(13)	0.0374(7)
C1	0.7096(10)	0.2450(9)	0.5034(6)	0.181(5)
C2	0.6021(10)	0.2321(8)	0.5094(6)	0.173(5)
C3	0.5467(8)	0.2858(6)	0.5456(4)	0.131(3)
C4	0.5429(6)	0.3582(4)	0.5225(3)	0.0917(19)
C5	0.5018(5)	0.4163(3)	0.5596(2)	0.0667(14)
C6	0.5075(3)	0.4905(2)	0.53456(17)	0.0463(9)
C7	0.4805(3)	0.5478(2)	0.57514(16)	0.0424(9)
C8	0.4874(2)	0.6229(2)	0.55234(15)	0.0363(8)
C9	0.4705(3)	0.6779(2)	0.59682(14)	0.0387(8)
C10	0.4871(2)	0.7538(2)	0.57796(14)	0.0341(8)
C11	0.4477(2)	0.8785(2)	0.58588(14)	0.0341(8)
C12	0.3825(2)	0.9270(2)	0.61939(16)	0.0404(8)
C13	0.3854(3)	1.0052(2)	0.60245(16)	0.0450(9)
C14	0.3315(4)	1.0500(3)	0.6436(2)	0.0604(12)
C15	0.3467(5)	1.0162(3)	0.5457(2)	0.0717(15)
C16	0.5496(2)	0.90321(19)	0.59325(13)	0.0310(7)
C17	0.6888(2)	0.9522(2)	0.55094(14)	0.0339(7)
C18	0.7219(3)	0.9772(2)	0.49454(15)	0.0395(8)
C19	0.6652(3)	1.0384(2)	0.47288(17)	0.0488(10)
C20	0.6779(3)	1.0495(3)	0.41292(18)	0.0541(11)
O11	0.7198(3)	1.0080(2)	0.38370(14)	0.0728(11)
N3	0.6343(5)	1.1050(4)	0.39313(19)	0.116(3)
C21	0.7545(2)	0.8960(2)	0.57305(13)	0.0323(7)

C22	0.7953(2)	0.7723(2)	0.58652(13)	0.0330(7)
C23	0.7989(2)	0.7738(2)	0.64873(13)	0.0319(7)
C24	0.7182(2)	0.7961(2)	0.73389(14)	0.0365(8)
C25	0.6165(3)	0.8007(2)	0.75616(15)	0.0432(9)
C26	0.5665(3)	0.7300(3)	0.74865(18)	0.0525(11)
C27	0.6178(4)	0.8229(4)	0.81583(18)	0.0659(14)
C28	0.7761(2)	0.8618(2)	0.74809(15)	0.0392(8)
C29	0.8036(3)	0.9886(2)	0.73393(16)	0.0392(8)
C30	0.7611(3)	1.0482(2)	0.69958(17)	0.0417(9)
C31	0.6541(3)	1.0581(3)	0.7050(2)	0.0557(12)
C32	0.6247(4)	1.1213(3)	0.6706(2)	0.0702(15)
C33	0.6231(3)	1.0675(3)	0.7647(2)	0.0612(13)
C34	0.9108(3)	0.9872(2)	0.72658(14)	0.0359(8)
C35	1.0465(2)	0.9412(2)	0.67577(15)	0.0380(8)
C36	1.0675(3)	0.9422(2)	0.61522(15)	0.0418(9)
C37	1.0929(3)	0.8770(2)	0.70261(15)	0.0405(8)
C38	1.0762(3)	0.7685(2)	0.75662(15)	0.0440(9)
C39	1.1144(3)	0.7130(2)	0.71676(15)	0.0404(9)
C40	1.0933(3)	0.6498(2)	0.63076(15)	0.0400(8)
C41	1.0158(3)	0.6045(2)	0.60600(14)	0.0384(8)
C42	1.1418(3)	0.6964(3)	0.58814(18)	0.0486(10)
C43	0.7965(9)	0.3833(4)	0.6239(5)	0.147(4)
C44	0.7587(6)	0.4412(4)	0.6620(3)	0.104(3)
C45	0.9982(3)	0.7395(3)	0.79359(17)	0.0532(11)
C46	1.0227(4)	0.6750(3)	0.82683(19)	0.0584(12)
C47	1.0994(4)	0.6900(3)	0.8692(2)	0.0721(15)
C48	0.9351(4)	0.6483(3)	0.8551(2)	0.0730(15)
C49	0.7885(4)	0.5503(5)	0.7135(2)	0.093(2)
C50	0.8296(4)	0.4990(3)	0.6743(2)	0.0637(13)
C51	0.8733(3)	0.5354(2)	0.62421(16)	0.0423(9)
C52	0.8024(3)	0.5813(2)	0.59398(15)	0.0393(8)
C53	0.7637(2)	0.6994(2)	0.56570(14)	0.0350(8)
C54	0.7782(3)	0.6916(2)	0.50480(15)	0.0471(10)
O16	0.3374(2)	0.78289(19)	0.70099(13)	0.0517(7)

Table S4. Anisotropic displacement parameters (Å^2) for pseudodesmin A (**3**). The anisotropic displacement factor exponent takes the form: $-2\pi^2[h^2 a^{*2}U_{11} + ... + 2 h k a^* b^* U_{12}]$

Atom	U_{11}	U_{22}	U_{33}	U_{23}	U_{13}	U_{12}
O1	0.0331(13)	0.0524(16)	0.0295(12)	-0.0030(11)	-0.0048(10)	0.0002(11)
O2	0.0328(13)	0.0440(14)	0.0356(13)	-0.0024(11)	0.0002(10)	-0.0035(11)
O3	0.0298(11)	0.0452(14)	0.0265(12)	-0.0025(10)	-0.0010(9)	0.0003(10)
O4	0.0280(12)	0.0523(16)	0.0322(13)	-0.0007(11)	-0.0027(10)	-0.0032(11)
O5	0.0289(12)	0.0555(17)	0.0298(12)	0.0001(11)	-0.0038(10)	0.0044(11)
O6	0.0472(15)	0.0659(19)	0.0355(14)	-0.0073(13)	-0.0119(12)	0.0001(14)
O7	0.0339(13)	0.0521(16)	0.0383(13)	-0.0108(12)	-0.0075(11)	-0.0012(12)
O8	0.0368(13)	0.0564(18)	0.0363(14)	-0.0086(12)	-0.0047(10)	0.0091(12)
O9	0.0289(14)	0.078(2)	0.0660(19)	0.0219(17)	0.0047(13)	0.0082(14)
O10	0.0334(14)	0.0556(18)	0.0578(17)	0.0204(14)	-0.0092(12)	0.0019(13)
O12	0.0670(19)	0.0432(16)	0.0358(14)	0.0031(12)	0.0040(13)	0.0074(14)
O13	0.0436(15)	0.071(2)	0.0309(14)	0.0049(13)	0.0074(11)	0.0127(14)
O14	0.0548(18)	0.0521(18)	0.0632(19)	-0.0014(15)	-0.0172(15)	-0.0107(15)
O15	0.0332(12)	0.0401(14)	0.0328(12)	0.0005(10)	-0.0023(10)	0.0045(10)
N1	0.0282(14)	0.0439(18)	0.0337(16)	0.0013(13)	0.0027(12)	-0.0037(13)
N2	0.0268(13)	0.0380(16)	0.0281(14)	-0.0013(12)	-0.0022(11)	-0.0020(12)
N4	0.0226(13)	0.0419(17)	0.0290(14)	0.0004(12)	0.0000(11)	0.0003(12)
N5	0.0272(14)	0.0435(17)	0.0243(13)	-0.0002(12)	0.0005(11)	-0.0002(12)
N6	0.0302(15)	0.0450(18)	0.0342(15)	-0.0080(13)	-0.0007(12)	-0.0016(13)
N7	0.0261(14)	0.0477(19)	0.0352(16)	-0.0111(13)	-0.0029(12)	0.0025(13)
N8	0.0285(15)	0.054(2)	0.0379(16)	-0.0007(14)	-0.0011(12)	0.0019(14)
N9	0.0302(15)	0.0496(19)	0.0365(16)	0.0061(14)	0.0018(12)	0.0074(14)
N10	0.0417(17)	0.0429(18)	0.0275(15)	0.0058(13)	0.0018(13)	0.0025(14)
C1	0.182(6)	0.179(6)	0.183(6)	-0.005(3)	-0.001(3)	-0.002(3)
C2	0.173(5)	0.172(5)	0.173(5)	-0.0016(14)	0.0005(14)	0.0011(14)
C3	0.133(3)	0.130(3)	0.131(3)	-0.0013(14)	0.0015(14)	0.0008(14)
C4	0.096(2)	0.089(2)	0.090(2)	-0.0020(13)	-0.0001(13)	0.0012(13)
C5	0.101(4)	0.044(3)	0.055(3)	-0.005(2)	-0.002(3)	-0.001(3)
C6	0.049(2)	0.047(2)	0.043(2)	-0.0007(17)	0.0020(18)	-0.0015(18)
C7	0.046(2)	0.044(2)	0.0372(19)	-0.0024(16)	-0.0060(16)	-0.0036(18)
C8	0.0298(17)	0.044(2)	0.0348(18)	-0.0001(15)	-0.0036(14)	-0.0028(15)
C9	0.0427(19)	0.045(2)	0.0285(17)	-0.0030(15)	0.0005(14)	-0.0028(17)
C10	0.0259(16)	0.045(2)	0.0314(17)	-0.0018(15)	-0.0045(13)	-0.0011(15)
C11	0.0271(16)	0.041(2)	0.0341(18)	-0.0023(15)	0.0003(13)	0.0000(15)
C12	0.0264(17)	0.054(2)	0.041(2)	-0.0023(17)	0.0059(14)	0.0011(16)
C13	0.045(2)	0.045(2)	0.045(2)	-0.0053(17)	0.0064(17)	0.0057(18)
C14	0.057(3)	0.058(3)	0.067(3)	-0.013(2)	0.013(2)	0.010(2)
C15	0.096(4)	0.062(3)	0.057(3)	-0.001(2)	0.000(3)	0.030(3)
C16	0.0261(16)	0.0352(18)	0.0317(17)	-0.0026(14)	0.0005(13)	0.0029(14)
C17	0.0278(17)	0.041(2)	0.0329(18)	-0.0031(14)	-0.0010(14)	-0.0046(15)
C18	0.0326(18)	0.049(2)	0.0371(19)	0.0038(16)	0.0009(14)	-0.0060(16)
C19	0.051(2)	0.050(3)	0.044(2)	0.0041(19)	0.0000(18)	0.0002(19)
C20	0.056(2)	0.058(3)	0.048(2)	0.006(2)	0.007(2)	0.008(2)
O11	0.088(3)	0.082(3)	0.0479(18)	0.0000(17)	0.0085(18)	0.030(2)
N3	0.170(6)	0.128(5)	0.050(3)	0.031(3)	0.034(3)	0.096(5)
C21	0.0264(16)	0.047(2)	0.0235(15)	-0.0014(14)	0.0029(13)	-0.0021(15)
C22	0.0247(15)	0.044(2)	0.0302(17)	0.0011(14)	0.0023(12)	0.0026(14)
C23	0.0288(16)	0.0371(19)	0.0297(16)	-0.0008(14)	0.0002(13)	-0.0002(14)

C24	0.0332(17)	0.051(2)	0.0252(16)	0.0002(15)	0.0015(13)	0.0007(16)
C25	0.0372(19)	0.063(3)	0.0290(18)	-0.0001(17)	0.0072(14)	-0.0026(18)
C26	0.039(2)	0.070(3)	0.048(2)	0.003(2)	0.0079(18)	-0.011(2)
C27	0.062(3)	0.096(4)	0.040(2)	-0.014(2)	0.019(2)	-0.008(3)
C28	0.0294(17)	0.057(2)	0.0308(17)	-0.0078(16)	0.0037(14)	-0.0020(16)
C29	0.0315(18)	0.047(2)	0.039(2)	-0.0134(16)	-0.0011(15)	0.0002(16)
C30	0.0347(19)	0.041(2)	0.049(2)	-0.0140(17)	-0.0054(16)	0.0054(16)
C31	0.034(2)	0.052(3)	0.081(3)	-0.026(2)	-0.012(2)	0.0046(18)
C32	0.050(3)	0.073(4)	0.088(4)	-0.028(3)	-0.017(2)	0.026(2)
C33	0.038(2)	0.058(3)	0.088(3)	-0.019(3)	0.014(2)	-0.002(2)
C34	0.0330(18)	0.041(2)	0.0341(18)	-0.0033(15)	-0.0040(14)	0.0024(15)
C35	0.0267(16)	0.049(2)	0.0383(19)	-0.0065(16)	-0.0037(14)	0.0020(15)
C36	0.0350(19)	0.054(2)	0.0365(19)	-0.0044(17)	-0.0009(15)	0.0051(17)
C37	0.0322(18)	0.054(2)	0.0347(18)	-0.0038(17)	-0.0019(14)	0.0030(17)
C38	0.0323(18)	0.064(3)	0.0361(19)	0.0074(18)	-0.0066(15)	-0.0017(18)
C39	0.0351(19)	0.048(2)	0.0377(19)	0.0166(17)	-0.0020(15)	-0.0034(16)
C40	0.0324(18)	0.050(2)	0.0377(19)	0.0068(16)	0.0069(15)	0.0087(17)
C41	0.0377(19)	0.045(2)	0.0327(19)	0.0062(15)	0.0049(14)	0.0147(16)
C42	0.047(2)	0.052(3)	0.047(2)	0.0051(19)	0.0131(18)	0.0063(19)
C43	0.214(11)	0.051(4)	0.177(9)	0.004(5)	-0.079(9)	-0.037(5)
C44	0.113(5)	0.112(6)	0.087(4)	0.043(4)	-0.043(4)	-0.053(5)
C45	0.043(2)	0.079(3)	0.038(2)	0.006(2)	-0.0021(17)	-0.002(2)
C46	0.073(3)	0.057(3)	0.046(2)	0.009(2)	0.001(2)	0.009(2)
C47	0.084(4)	0.083(4)	0.050(3)	0.020(3)	-0.021(3)	-0.007(3)
C48	0.085(4)	0.071(3)	0.063(3)	0.019(3)	0.008(3)	-0.009(3)
C49	0.056(3)	0.183(8)	0.040(3)	-0.006(3)	0.009(2)	-0.032(4)
C50	0.056(3)	0.072(3)	0.063(3)	0.030(3)	-0.008(2)	-0.014(2)
C51	0.045(2)	0.042(2)	0.040(2)	0.0005(16)	0.0006(16)	-0.0004(17)
C52	0.044(2)	0.043(2)	0.0315(18)	-0.0039(15)	0.0020(15)	-0.0041(17)
C53	0.0291(16)	0.046(2)	0.0301(17)	-0.0011(15)	-0.0007(13)	0.0041(15)
C54	0.058(2)	0.052(2)	0.0315(19)	-0.0046(16)	-0.0015(17)	0.018(2)
O16	0.0355(15)	0.061(2)	0.0588(17)	0.0102(16)	0.0123(12)	0.0043(13)

Spectral Data for Pseudodesmin B (4)

Figure S30. HR-ESI-MS spectrum of **4** (positive mode).

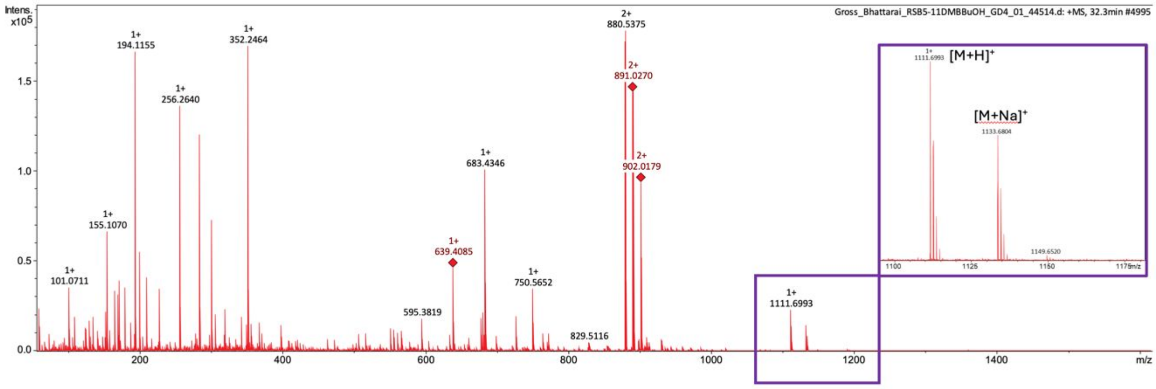

Table S5. ^{1}H and ^{13}C NMR assignments of pseudodesmin B (**4**) in d_3-CH_3CN in comparison with published values.

Residue	Atom	Pseudodesmin B (this study)[a]		Pseudodesmin B (lit. data)[b]		Δ(δH)	Δ(δC)
		δ_H [ppm]	δ_C [ppm]	δ_H [ppm]	δ_C [ppm]		
HDA	CO-1		175.42		175.14		+0.28
	CH_2-2	2.38/2.43	44.71	2.35/2.43	44.76	+0.03/±0.00	-0.05
	CH-3	3.97	69.66	3.96	69.64	+0.01	+0.02
	CH_2-4	1.48	38.27	1.48	38.29	±0.00	-0.02
	CH_2-5	1.31/1.41	26.35	1.31/1.40	26.36	±0.00 / +0.01	-0.01
	CH_2-6	1.28	30.25	1.28	30.12	±0.00	+0.13
	CH_2-7	1.28	30.02	1.28	30.12	±0.00	-0.10
	CH_2-8	1.28	32.58	1.26	32.59	+0.02	-0.01
	CH_2-9	1.28	23.39	1.29	23.44	-0.01	-0.05
	CH_2-10	0.89	14.40	0.88	14.43	+0.01	-0.03
Leu1	NH	8.07 brs		7.94		+0.13	
	CO		175.42		175.40		+0.02
	CH-α	3.89	53.66	3.89	53.76	±0.00	-0.10
	CH_2-β	1.68/1.75	39.32	1.66/1.74	39.42	+0.02 / +0.01	-0.10
	CH-γ	1.67	25.40	1.67	25.45	±0.00	-0.05
	CH_3-δ	0.90	22.11	0.90	22.15	±0.00	-0.04
	CH_3-ε	0.94	23.22	0.93	23.25	+0.01	-0.03
Gln2	NH	8.80 brs		8.78		±0.02	
	CO		176.69		176.68		+0.01
	CH-α	3.98 m	57.60	3.97	57.62	+0.01	-0.02
	CH_2-β	2.02 m	26.58	2.01	26.56	+0.01	+0.02
	CH_2-γ	2.38 m	31.22 w	2.37	32.14	+0.01	+0.08
	CO-δ		n.o.		176.09		+0.03
	NH_2	5.87 s / 6.44 s		5.82 s / 6.42 s		+0.05 / +0.02	
Thr3	NH	8.20 d 6.4		8.20 d 7.1		±0.00	
	CO		174.32		174.34		-0.02
	CH-α	3.99 m	61.80	3.99	61.84	±0.00	-0.04
	CH-β	5.32 dq	70.26	5.33	70.29	-0.01	-0.03
	CH_3-γ	1.31 m	18.61	1.30	18.64	+0.01	-0.03
Val4	NH	7.43 d 6.1		7.41 d 6.3		+0.02	
	CO		174.68		174.70		-0.02
	CH-α	3.48 dd 6.1, 10.6	65.09	3.48	65.10	±0.00	-0.01
	CH-β	2.18	29.97	2.16	30.02	+0.02	-0.05
	CH_3-γ	0.91	19.43	0.90	19.47	+0.01	-0.04
	CH_3-ε	0.96	21.22	0.95	21.27	+0.01	-0.05
Leu5	NH	7.80 d 3.7		7.77 d 4.0		+0.03	
	CO		173.66		173.68		-0.02
	CH-α	3.97 m	55.70	3.97	55.75	±0.00	-0.05
	CH_2-β	1.51/1.67	40.61	1.50/1.67	40.67	+0.01 / ±0.00	-0.06
	CH-γ	1.78	25.48	1.77	25.54	+0.01	-0.06
	CH_3-δ	0.86	23.32	0.86	23.36	±0.00	-0.04
	CH_3-ε	0.88	21.28	0.87	21.32	+0.01	-0.04
Ser6	NH	7.15 d 8.2		7.14 d 8.3		+0.01	
	CO		172.03		172.03		±0.00
	CH-α	4.33 brd 8.2	56.42	4.32	56.47	+0.01	-0.05
	CH_2-β	3.82/4.15	64.65	3.81/4.15	64.71	+0.01 / ±0.00	-0.06
	OH	5.01 brs		4.99		+0.02	
Leu7	NH	7.13 d 5.3		7.11 d 6.2		+0.02	
	CO		173.90		173.88		+0.02
	CH-α	4.15	54.86	4.14	54.86	+0.01	±0.00
	CH_2-β	1.62/1.91	41.83	1.61/1.89	41.90	+0.01 / +0.02	-0.07
	CH-γ	1.89	25.52	1.88	25.56	+0.01	-0.04
	CH_3-δ	0.91	21.43	0.90	21.45	+0.01	-0.02
	CH_3-ε	0.99 d 6.2	23.39	0.98	23.42	+0.01	-0.03
Ser8	NH	7.97 d 8.8		7.95 d 9.0		+0.02	
	CO		172.03		172.02		+0.01
	CH-α	4.45	57.04	4.44	57.10	+0.01	-0.06
	CH_2-β	3.67/3.86	63.23	3.66/3.86	63.29	+0.01 / ±0.00	-0.06
Val9	NH	6.71 d 10.0		6.71 d 10.0		±0.00	
	CO		170.05		170.15		-0.10
	CH-α	4.52 dd 3.3, 10.0	57.04	4.52	57.07	±0.00	-0.03
	CH-β	2.23	30.13	2.23	30.18	±0.00	-0.05
	CH_3-γ	0.73 d 6.8	17.17	0.72	17.21	+0.01	-0.04
	CH_3-δ	0.83 d 6.8	19.46	0.82	19.49	+0.01	-0.03

[a]400/100 MHz, 298 K, referenced to the residual solvent signal of CD_3CN at δ_H/δ_C = 1.94/1.32; [b]700/175 MHz, 298 K, referenced to TMS, values taken from Tetrahedron 65 (2009) 4173–4181; w = weak resonance; n.o. = not observed - this resonance was not observed in d_3-CH_3CN, but in a data set, recorded in d_3-MeOH.

Figure S31. 400 MHz ^{1}H NMR spectrum of **4** in d_3-CH_3CN.

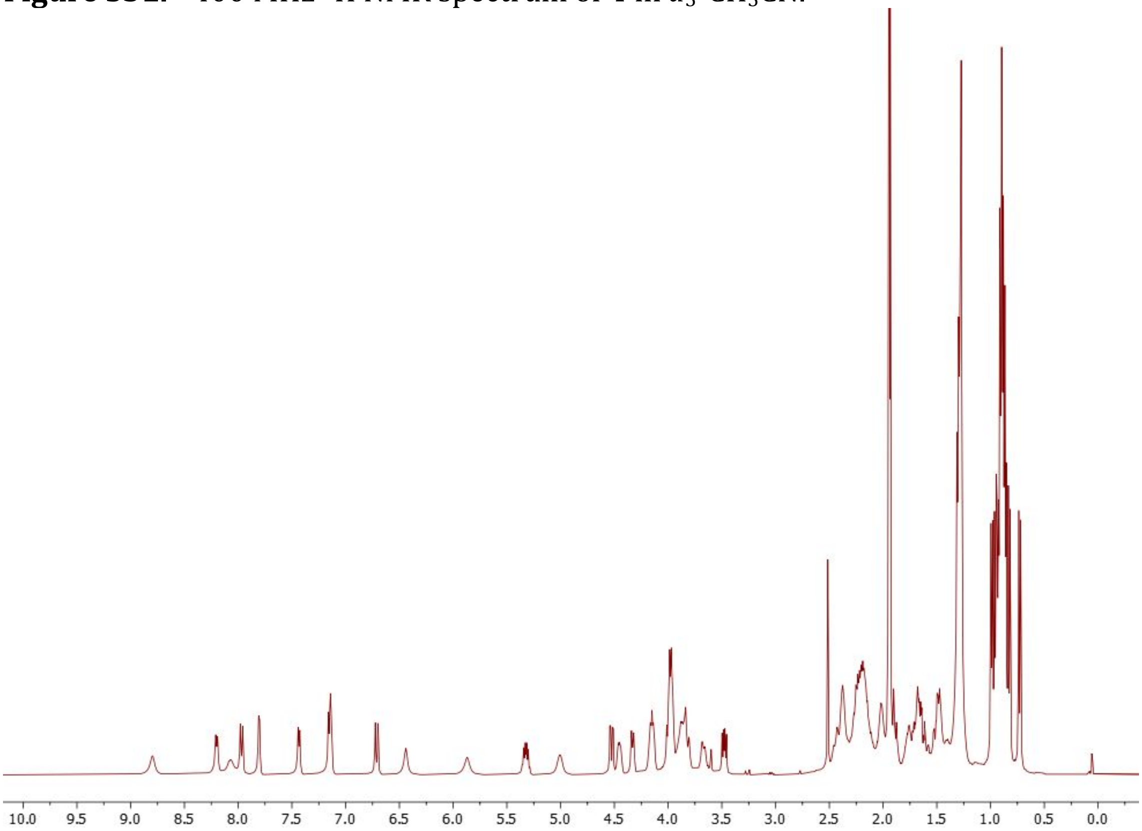

Figure S32. 400 MHz ^{13}C NMR spectrum of **4** in d_3-CH_3CN.

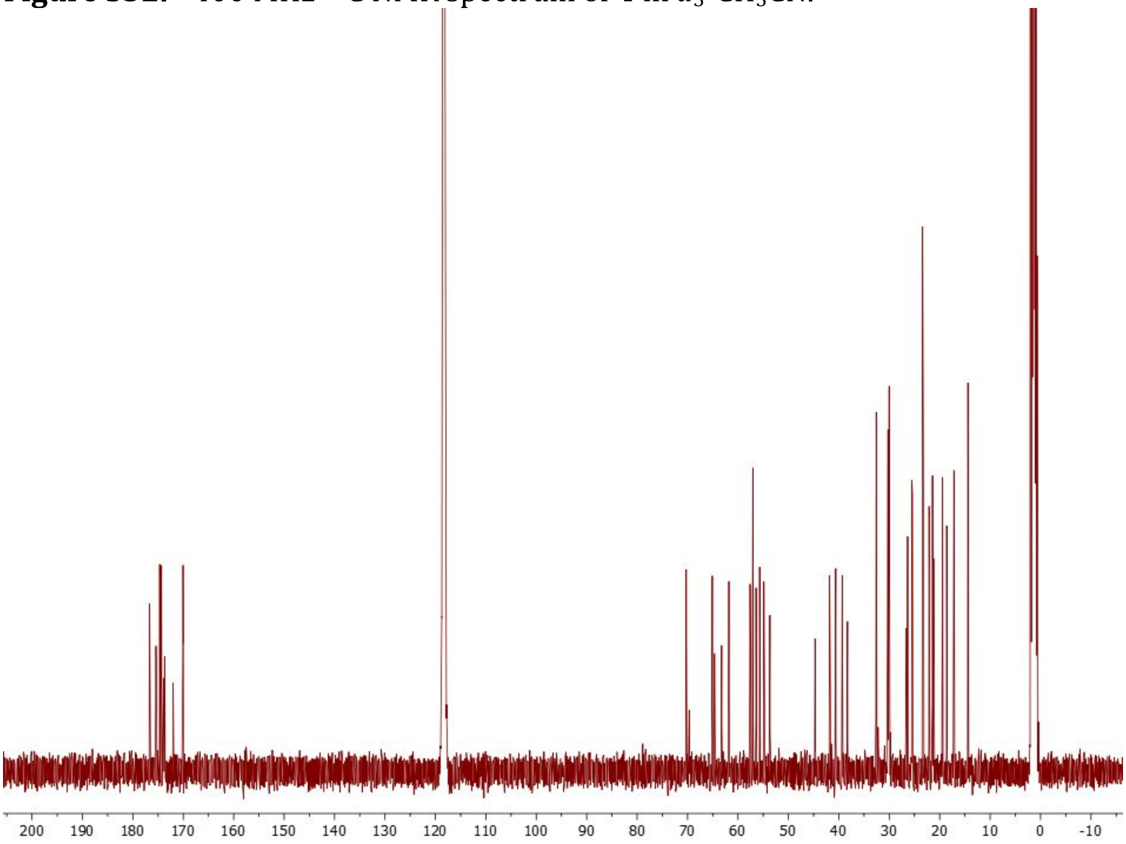

Figure S33. 400 MHz multiplicity edited ^1H-^{13}C-HSQC NMR spectrum of **4** in d_3-CH_3CN. CH_2-groups are indicated by blue crosspeaks, while CH and CH_3 groups occur as red crosspeaks.

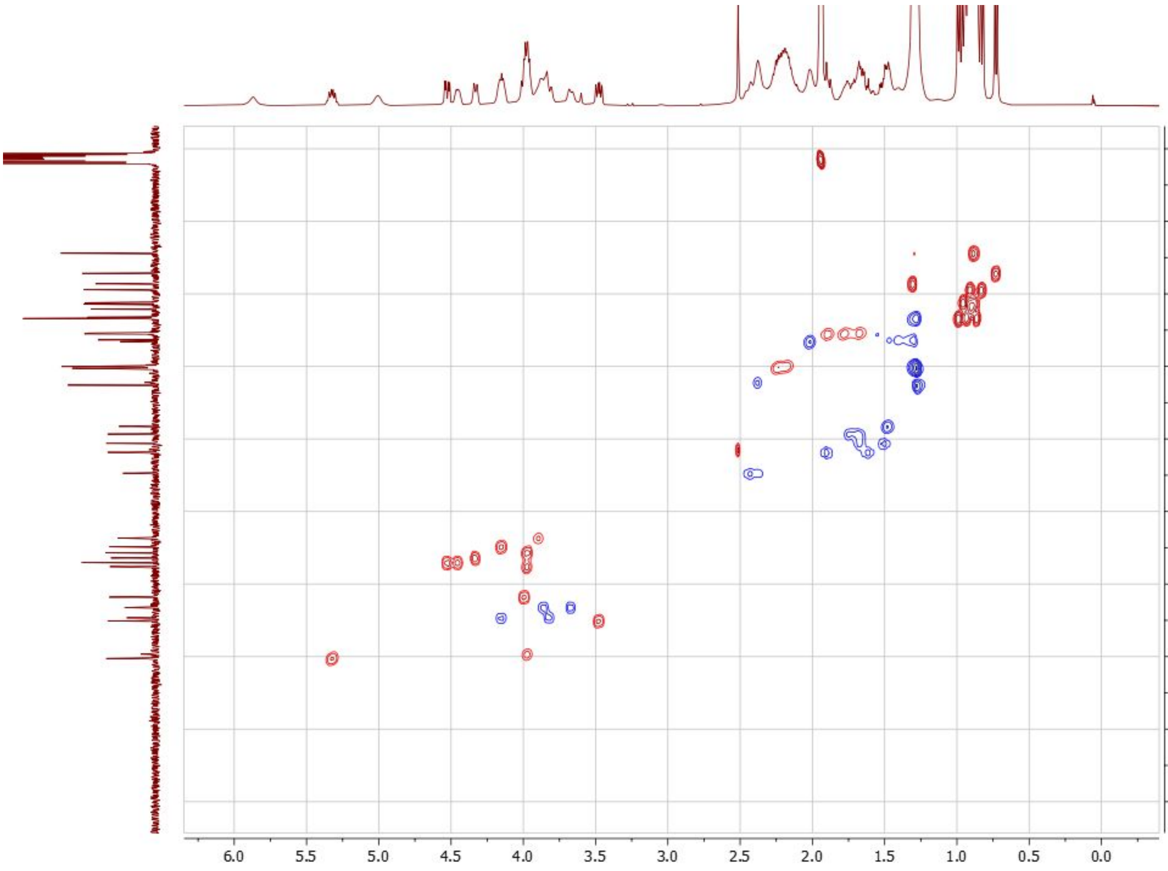

Spectral Data for Salamandamide B (5)

Figure S34. HR-ESI-MS spectrum of **5** (positive mode).

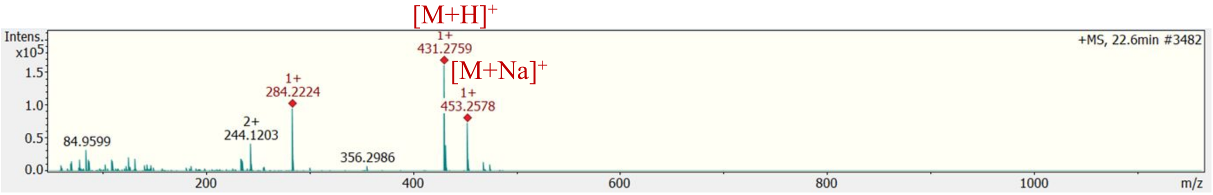

Figure S35. HR-ESI-TOF-MS/MS spectrum of **5**.

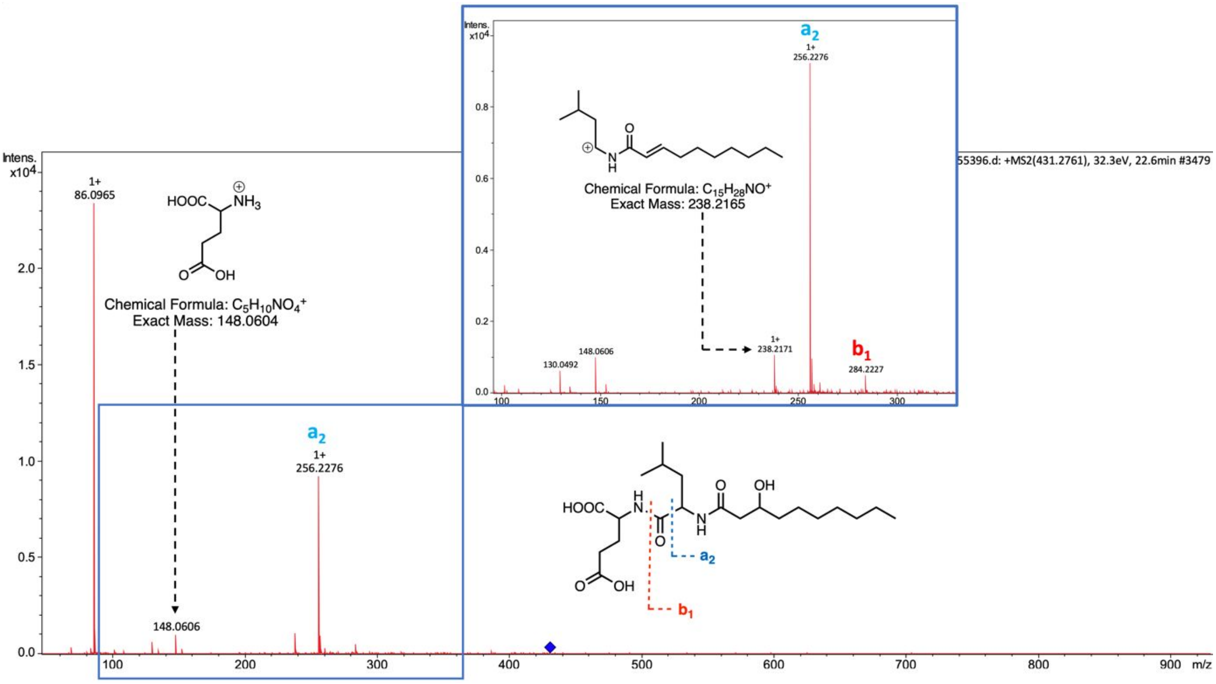

Figure S36. 700 MHz ^{1}H NMR spectrum of **5** in d_6-DMSO. Impurities are marked with asterisks.

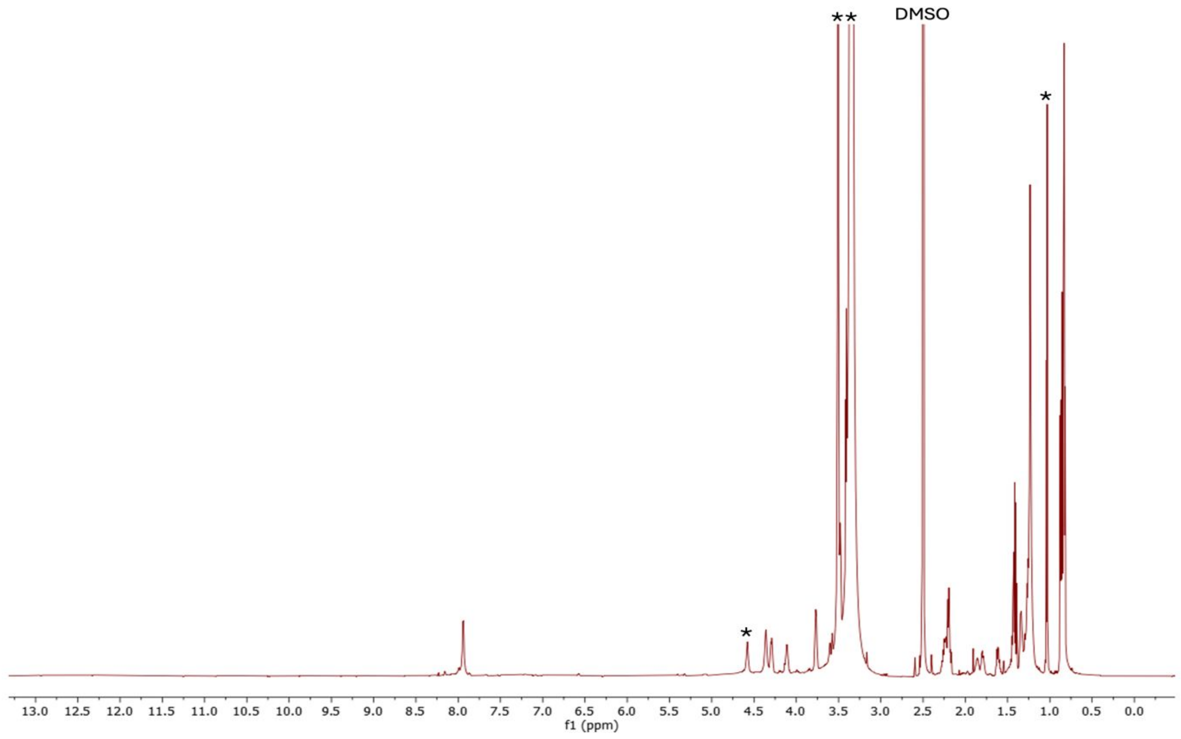

Figure S37. 175 MHz ^{13}C NMR spectrum of **5** in d_6-DMSO. Impurities are marked with asterisks.

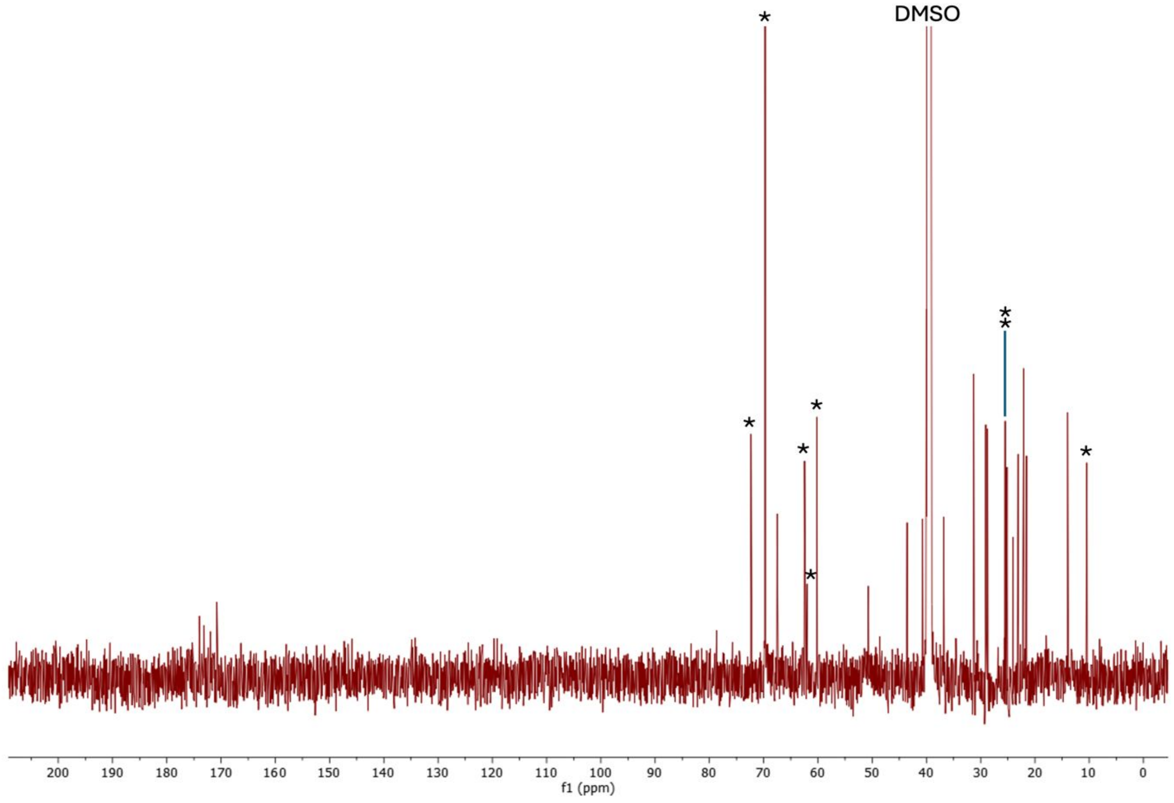

Figure S38. 700 MHz multiplicity edited ^{1}H-^{13}C-HSQC NMR spectrum of **5** in d_6-DMSO. CH_2-groups are indicated by blue crosspeaks, while CH and CH_3 groups occur as red crosspeaks.

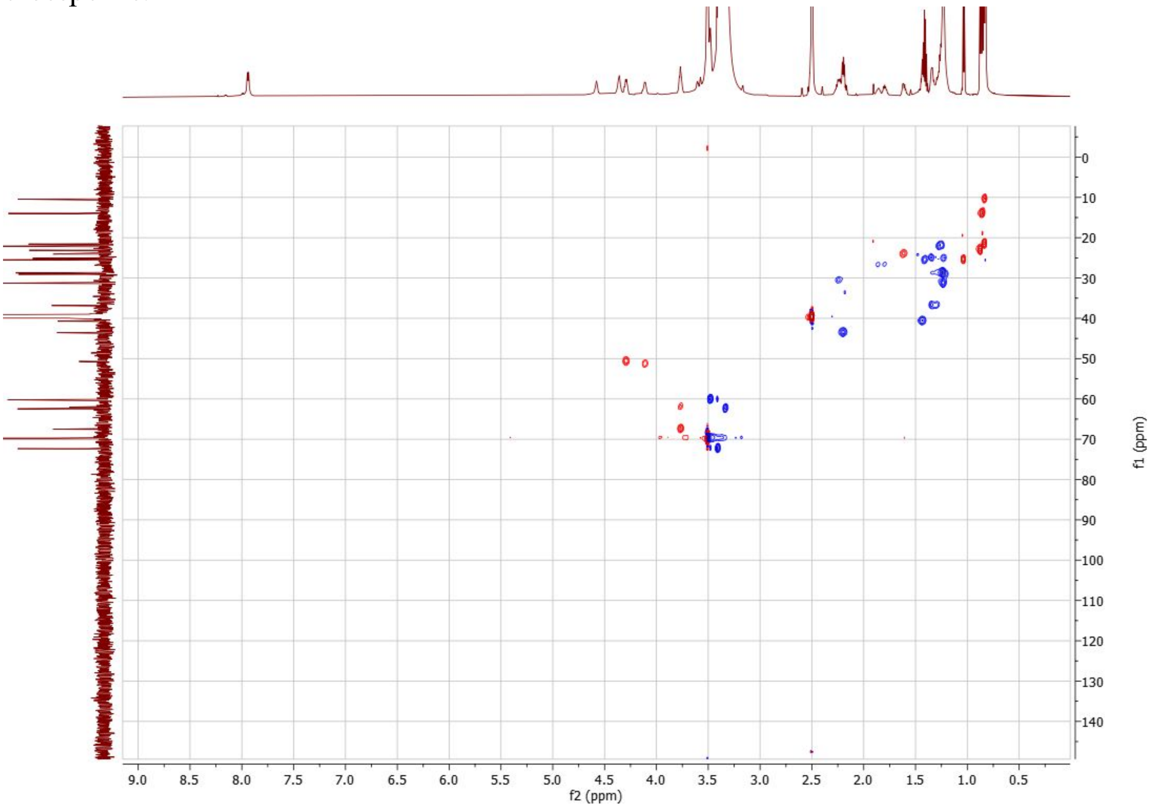

Figure S39. 700 MHz ^{1}H-^{1}H-COSY NMR spectrum of **5** in d_6-DMSO. Bold lines in the insert visualize the observed correlations.

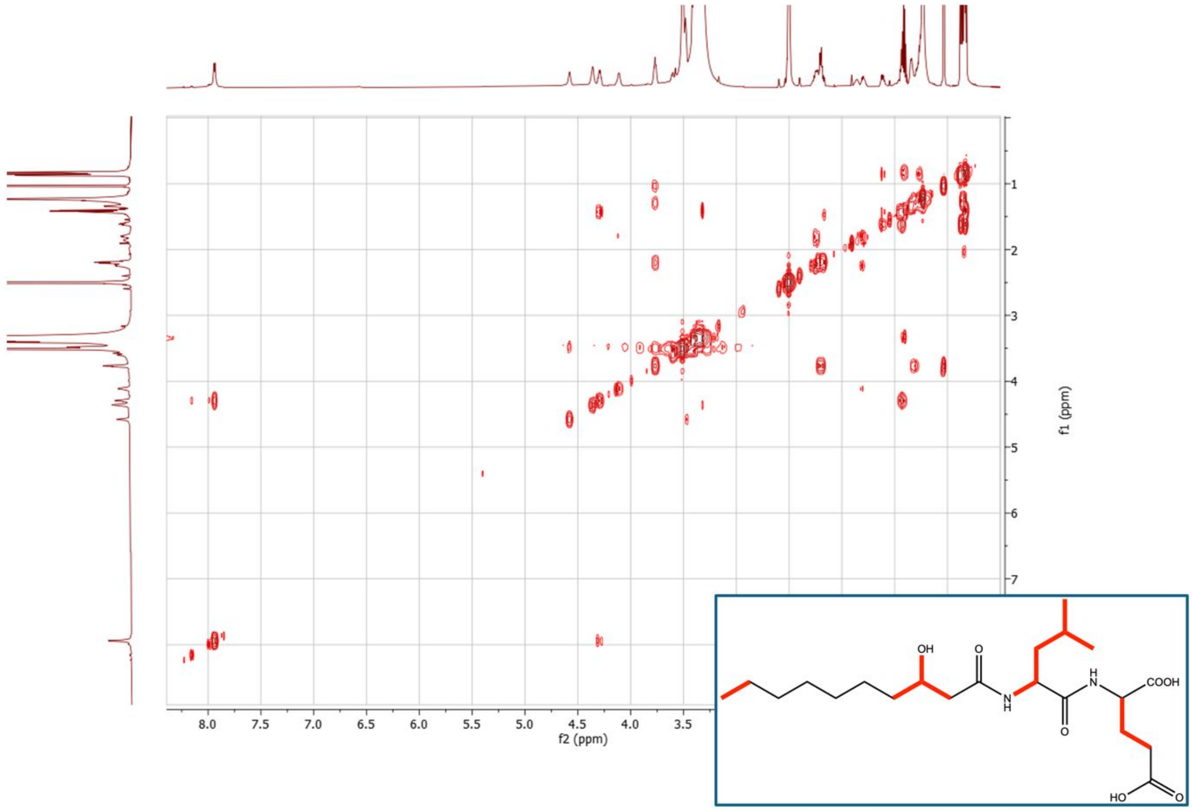

Figure S40. 700 MHz ^{1}H-^{13}C-HSQC-TOCSY spectrum of **5** in d_6-DMSO. Horizontal and vertical lines are showing, color-code-assisted the three spin systems given in **5**.

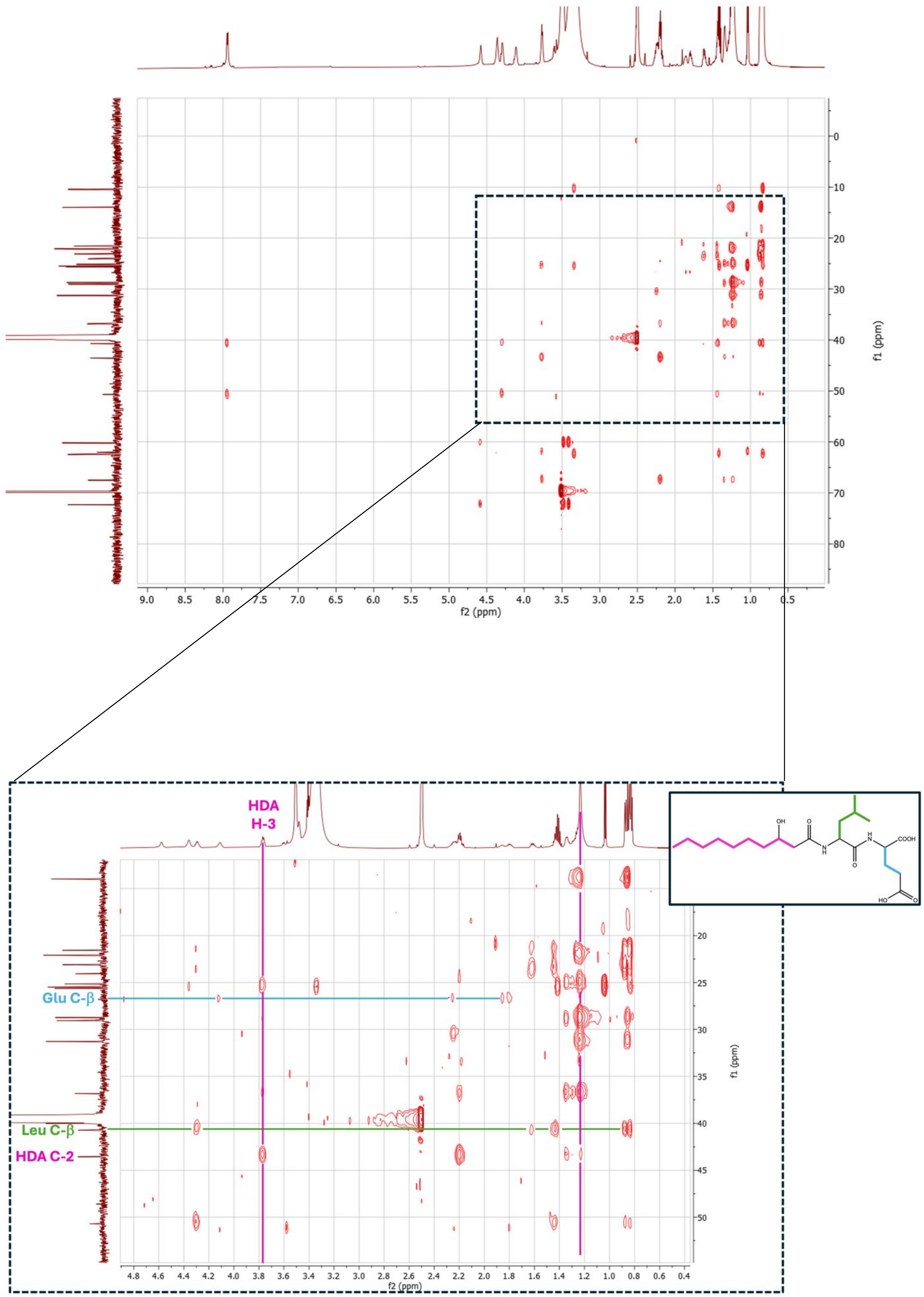

Figure S41. 700 MHz 1D selective gradient ^{1}H-^{1}H-TOCSY spectra of **5** in d_6-DMSO.
Top: irradiated at Leu-H$_\alpha$
Bottom: irradiated at Glu-H$_\alpha$

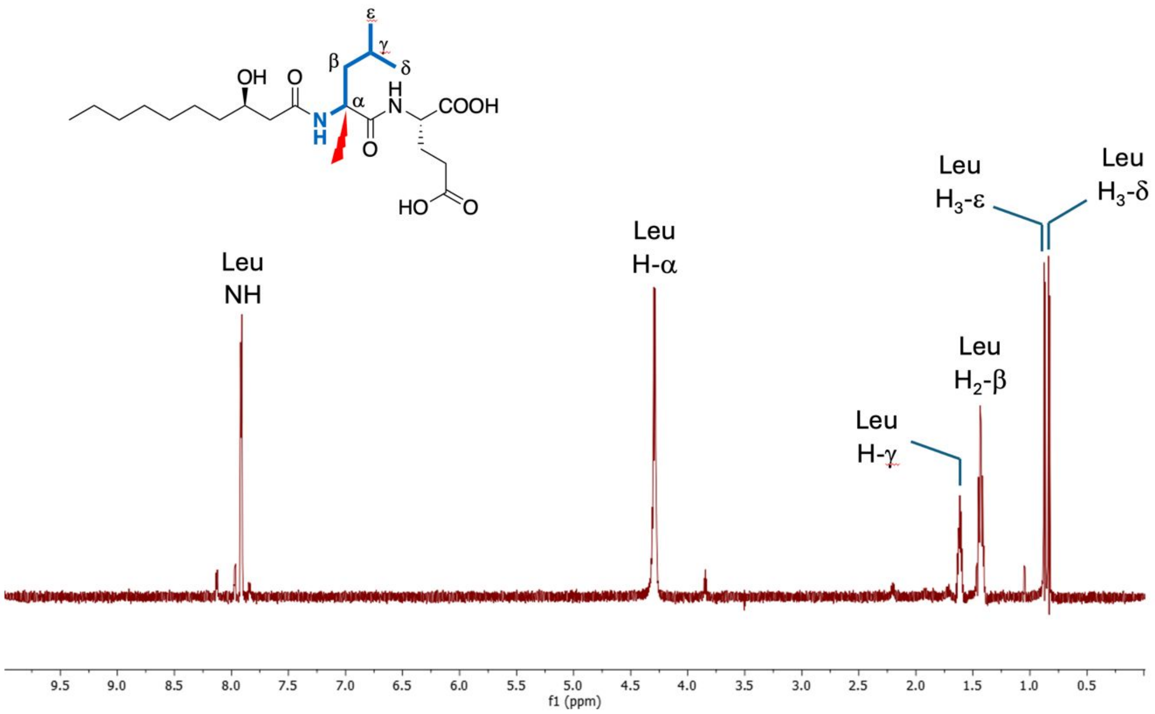

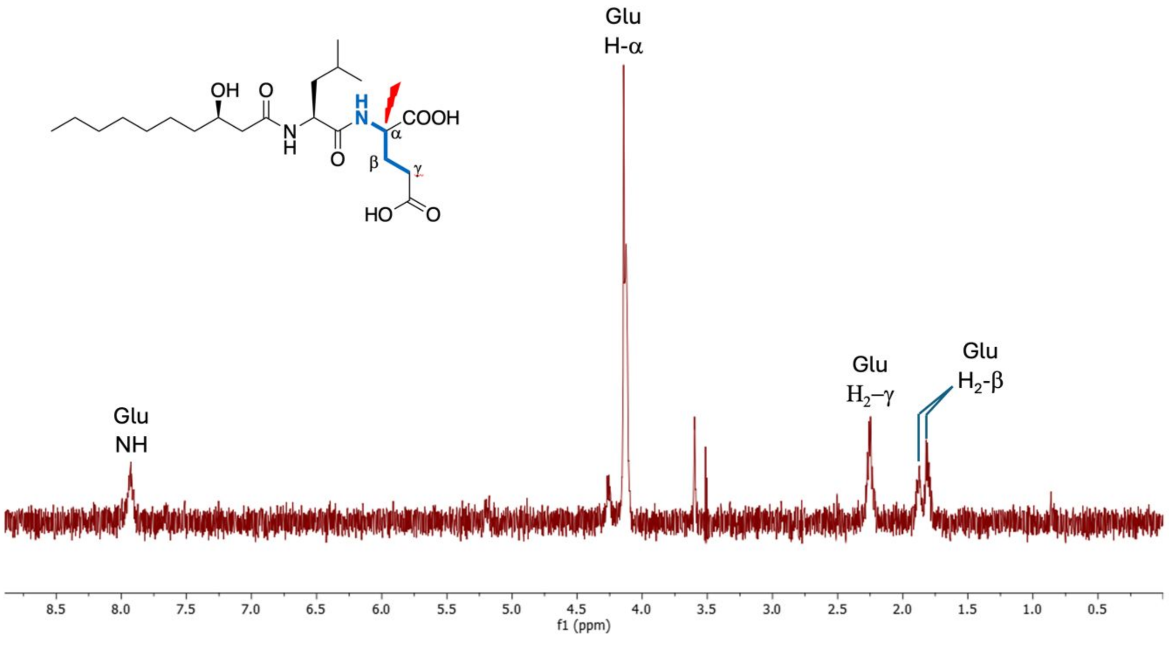

Figure S42. 700 MHz ^{1}H-^{13}C-HMBC NMR spectrum of **5** in d_6-DMSO.

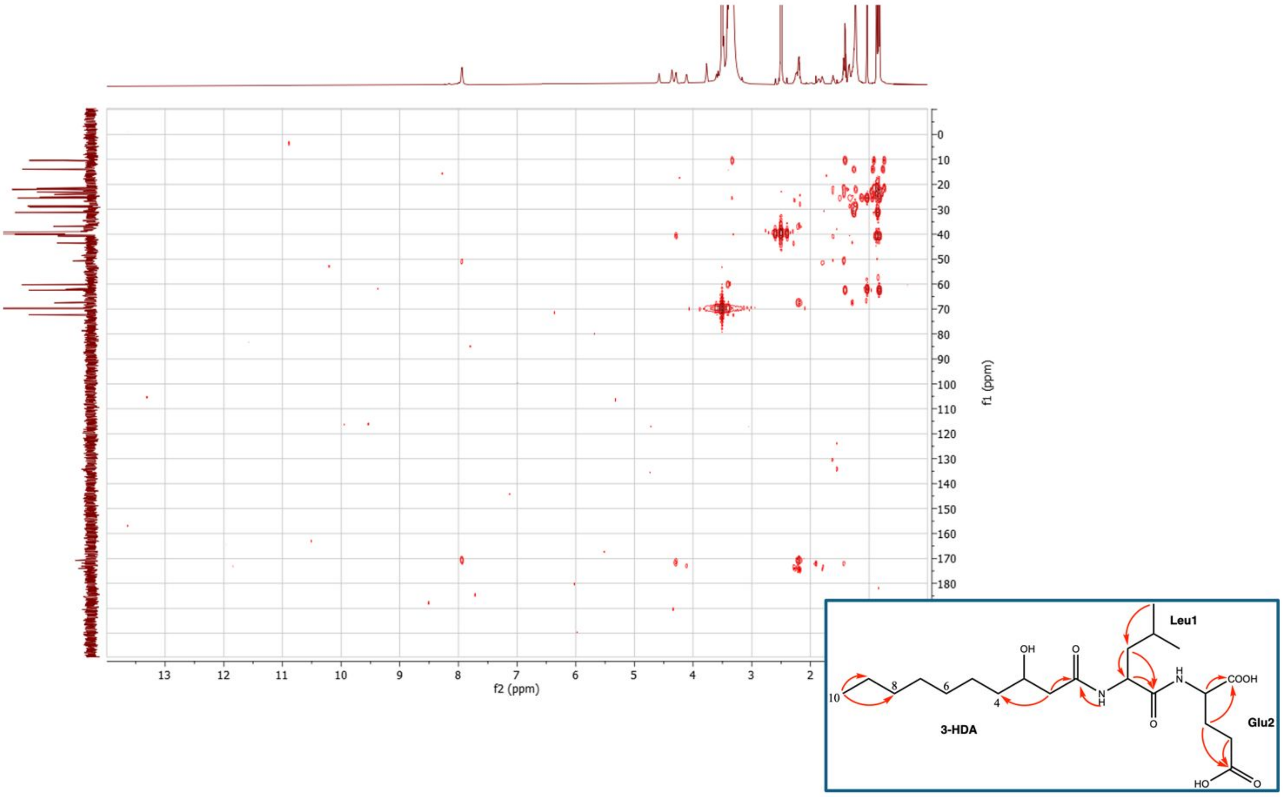

Figure S43. 700 MHz ^{1}H-^{1}H-NOESY NMR spectrum of **5** in d_6-DMSO.

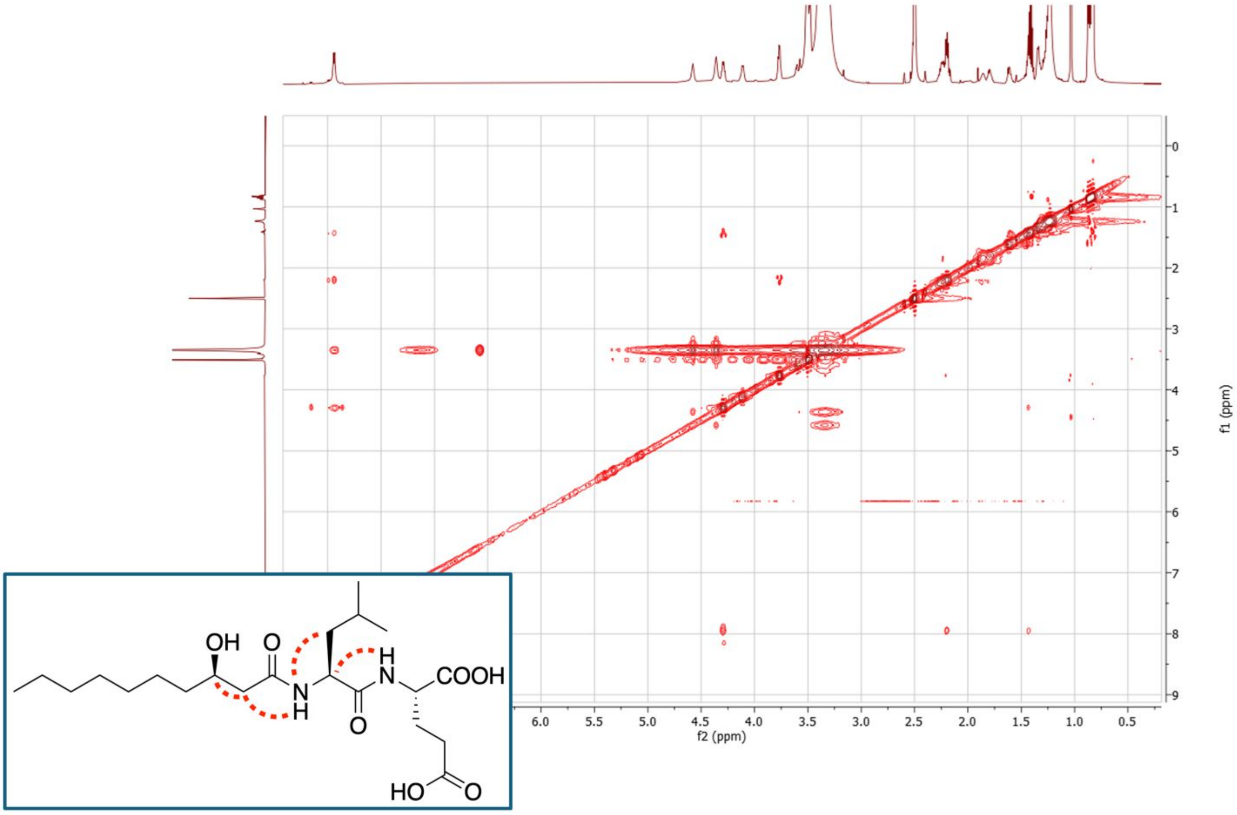

Figure S44. 700 MHz ^1H-^{15}N-HSQC NMR spectrum of **5** in d_6-DMSO.

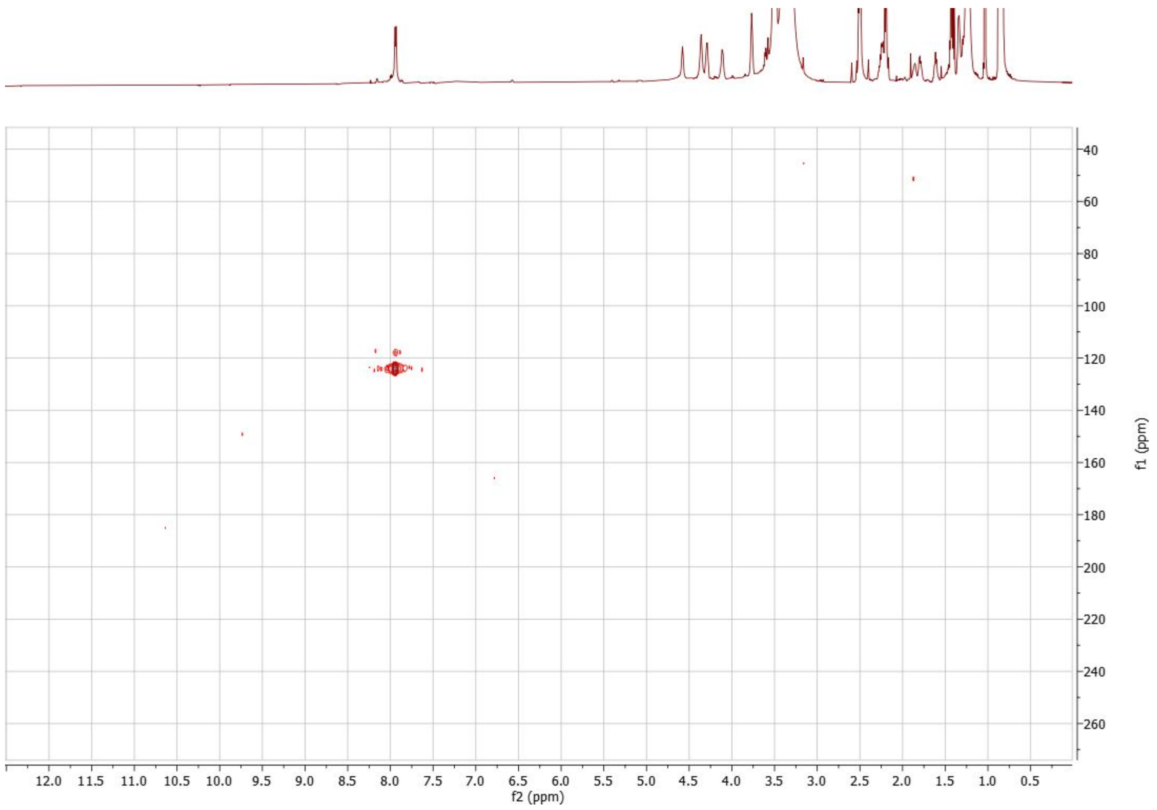

Figure S45. Comparative LC/MS analysis of the *visc* mutants. Ion extracted (m/z 431.3 to 431.6) chromatogram (color code of the mass traces: Δ*viscA* , Δ*viscB* , Δ*viscC*).

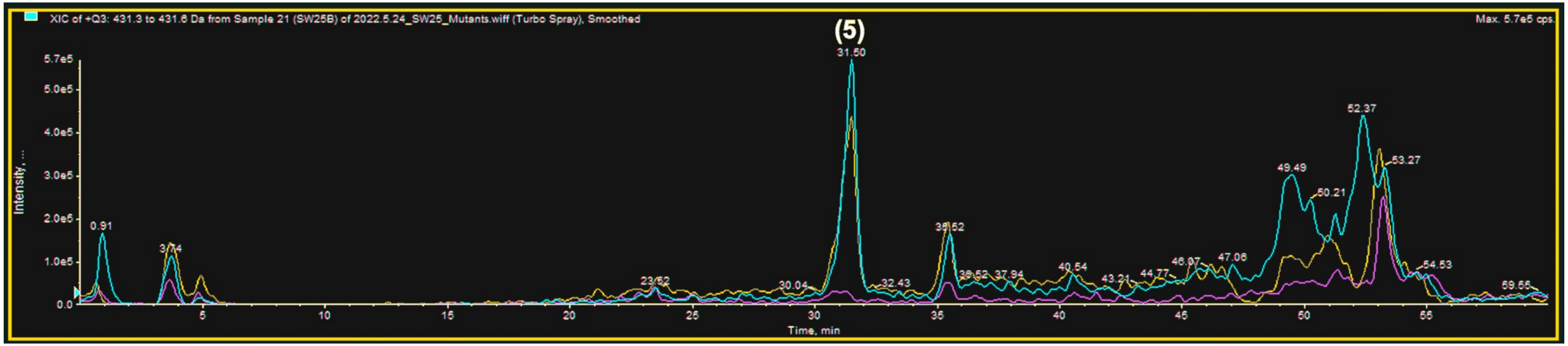

Biological Assays

Table S6. Results of the Protease Inhibition Assays.

Protease	Salamandamide A (**2**)	Residual activity
SARS-CoV-2 main protease (M^{pro})[a]	50 µM	99%
Human cathepsin L[b]	50 µM	98%
Human leukocyte elastase[c]	50 µM	100%

[a]Duplicate measurements on a Fluostar Optima plate reader, λ_{ex} 360 nm, λ_{em} 460 nm, pH 7.2, 37 °C, substrate Boc-Abu-Tle-Leu-Gln-AMC, [S] = 50 µM (= 1.03 K_m), 4% DMSO. The reaction was followed for 10 min.

[b]Duplicate measurements on a Varian Cary Bio 50 spectrophotometer, λ_{em} 405 nm, pH 6.0, 37 °C, substrate Z-Phe-Arg-pNA, [S] = 100 µM (= 5.88 K_m), 2% DMSO. The reaction was followed for 60 min.

[c]Duplicate measurements on a Fluostar Optima plate reader, λ_{ex} 360 nm, λ_{em} 460 nm, pH 7.8, 37 °C, substrate MeoSuc-Ala-Ala-Pro-Val-AMC, [S] = 400 µM (= 4.66 K_m), 6% DMSO. The reaction was followed for 10 min.

Figure S46. Effect of lipopeptides 2 and 3 on SARS-CoV-2 infection. Caco-2 cells were infected with SARS-CoV-2-mNG and treated with compounds **2** and **3** at two concentrations, and RDV as positive control. **A**) The graph on the left shows the infection rate calculated as n° of infected cells/total number of cells. The graph on the right shows the total number of cells stained by Hoechst (n=1). **B**) Representative fluorescence microscopy images of mock and SARS-CoV-2-mNG (mNG: mNeonGreen) infected, as well as infected and treated Caco-2 cells taken 48 h post-infection. Cellular nuclei are shown as Hoechst-positive cells.

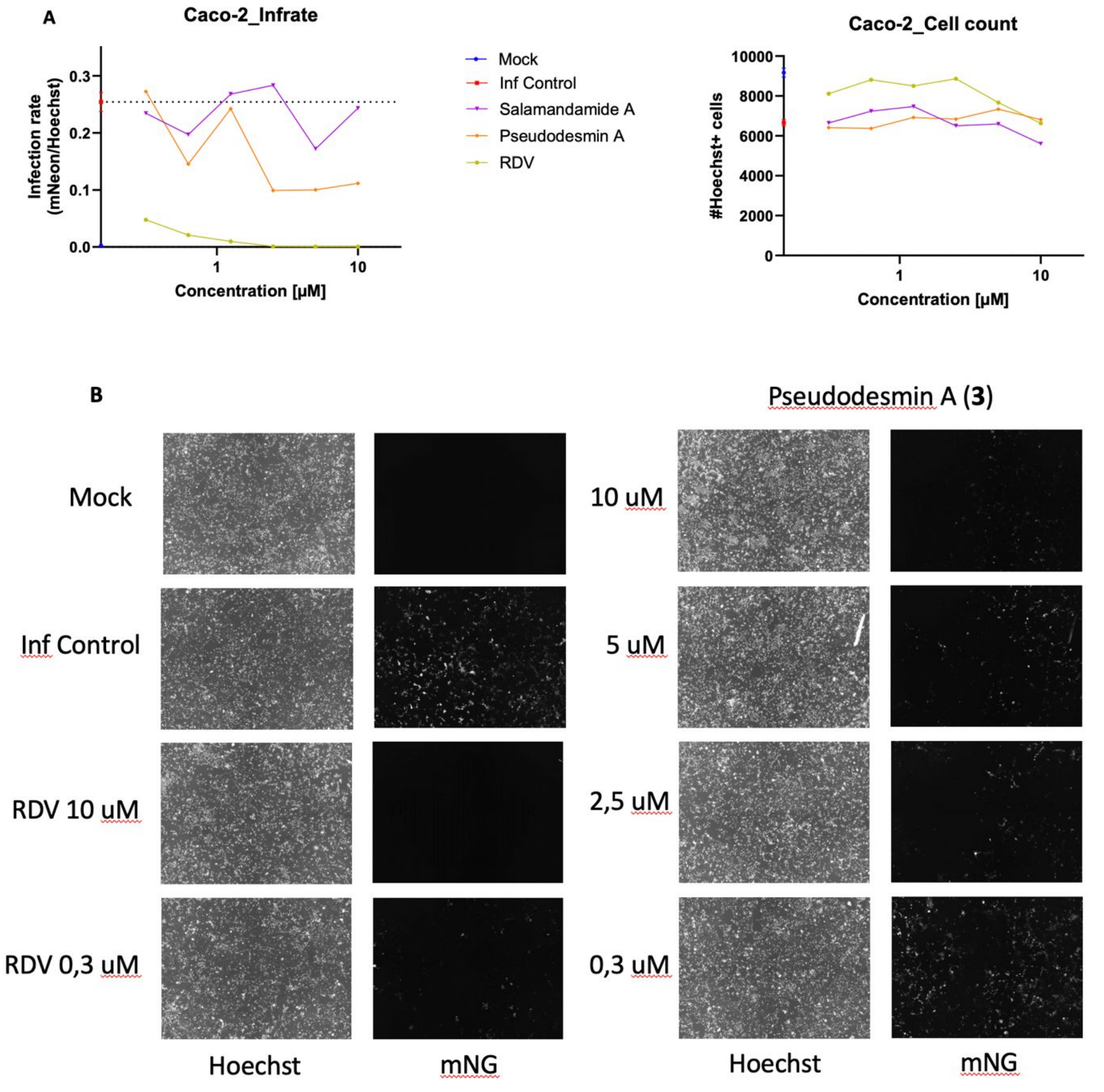

Figure S47. Effect of lipopeptides 2 and 3 on SARS-CoV-2 infection upon Pre-treatment of Caco-2 cells. Caco-2 cells were treated with compounds **2** and **3** at two concentrations, and remdesivir (RDV) as positive control. 2:30 hours after treatment, cells were infected with green fluorescent virus SARS-CoV-2-mNG (mNG: mNeonGreen). **A**) The graph on the left shows the infection rate calculated as n° of infected cells/total number of cells. The graph on the right shows the total number of cells stained by Hoechst. The table below reports the EC_{50} value for the active molecules (n=3; +/- SEM). **B**) Representative fluorescence microscopy images of mock and SARS-CoV-2 infected, as well as infected and treated Caco-2 cells taken 48 h post-infection. Cellular nuclei are shown as Hoechst-positive cells.

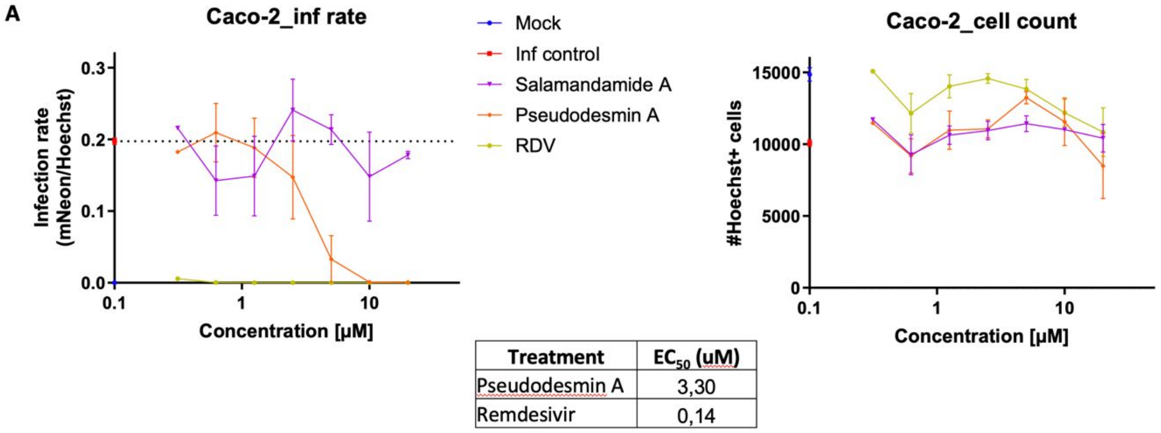

Treatment	EC_{50} (uM)
Pseudodesmin A	3,30
Remdesivir	0,14

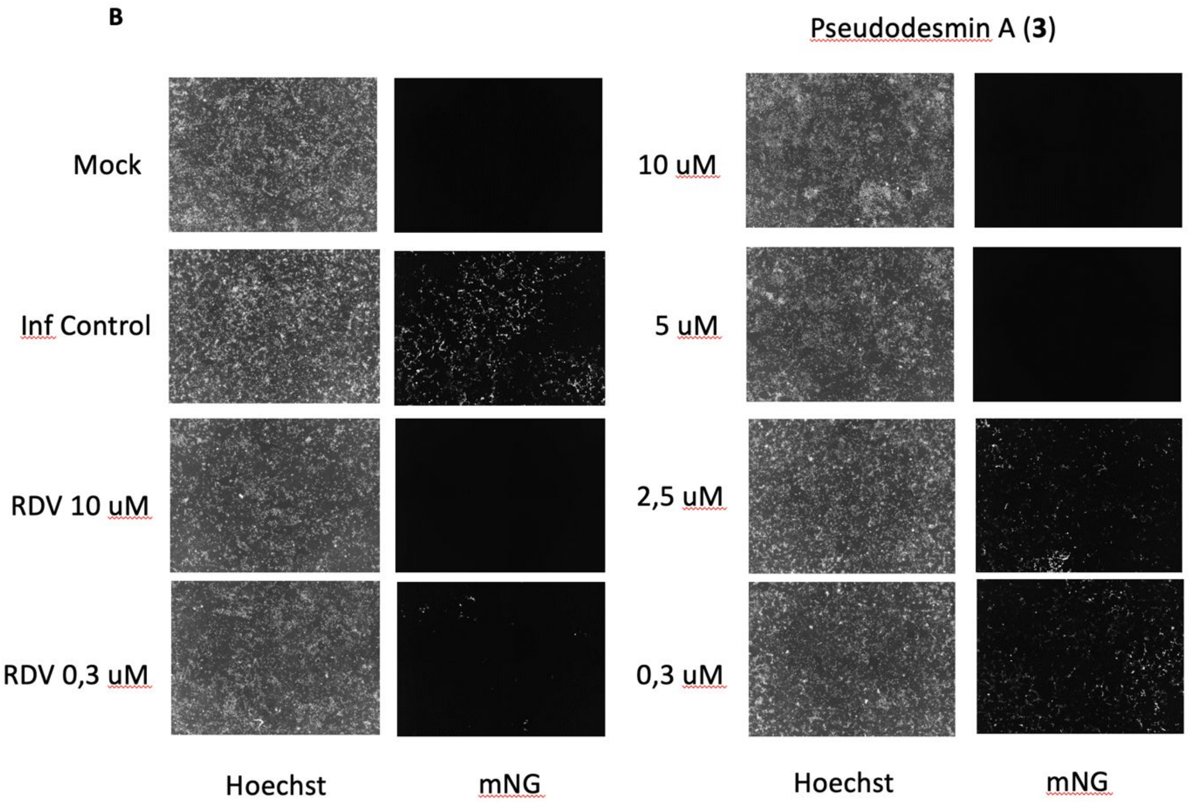

Table S7. Results of the antimicrobial and cytotoxicity assays

Organism	(2) Salamandamide A
Antibacterial Assays	MIC [µg/mL]
Bacillus subtilis 168	>32
Staphylococcus aureus ATCC29213	>32
Enterococcus faecalis ATCC29212	>32
Enterococcus faecium BM4147-1	>32
Enterococcus faecium BM4147 (VRE)	>32
Enterococcus faecium 6011 (VRE)	>32
Enterococcus faecium 115.2	>32
Enterococcus faecium K209.5	>32
Enterococcus faecium U200 (VRE)	>32
Escherichia coli ATCC25922	>32
Escherichia coli C600 Tn10 Δ*lpxC* Δ*tolC*	>32
Klebsiella pneumoniae ATCC12657	>32
Enterobacter aerogenes ATCC13048	>32
Pseudomonas aeruginosa ATCC27853	>32
Acinetobacter baumannii 09987	>32
Neisseria gonorrhoeae ATCC19424	>32
Neisseria gonorrhoeae S1441	>32
Antifungal Assays	MIC [µg/mL]
Candida albicans ATCC90028	>32
Candida albicans Tü01	>32
Candida albicans Tü02	>32
Candida albicans Tü03	>32
Candida glabrata Tü04	>32
Candida tropicalis Tü05	>32
Cytotoxicity Assay	IC_{50} [µg/mL]
HeLa cell line	>32

VRE, vancomycin resistant *Enterococcus*
